# Chromosome-level genome assemblies of two littorinid marine snails indicate genetic basis of intertidal adaptation and ancient karyotype evolved from bilaterian ancestors

**DOI:** 10.1093/gigascience/giae072

**Published:** 2024-09-25

**Authors:** Yan-Shu Wang, Meng-Yu Li, Yu-Long Li, Yu-Qiang Li, Dong-Xiu Xue, Jin-Xian Liu

**Affiliations:** CAS Key Laboratory of Marine Ecology and Environmental Sciences, Institute of Oceanology, Chinese Academy of Sciences, Qingdao 266071, China; Laboratory for Marine Ecology and Environmental Science, Qingdao Marine Science and Technology Center, Qingdao 266237, China; University of Chinese Academy of Sciences, Beijing 100049, China; CAS Key Laboratory of Marine Ecology and Environmental Sciences, Institute of Oceanology, Chinese Academy of Sciences, Qingdao 266071, China; Laboratory for Marine Ecology and Environmental Science, Qingdao Marine Science and Technology Center, Qingdao 266237, China; University of Chinese Academy of Sciences, Beijing 100049, China; CAS Key Laboratory of Marine Ecology and Environmental Sciences, Institute of Oceanology, Chinese Academy of Sciences, Qingdao 266071, China; Laboratory for Marine Ecology and Environmental Science, Qingdao Marine Science and Technology Center, Qingdao 266237, China; CAS Key Laboratory of Marine Ecology and Environmental Sciences, Institute of Oceanology, Chinese Academy of Sciences, Qingdao 266071, China; Laboratory for Marine Ecology and Environmental Science, Qingdao Marine Science and Technology Center, Qingdao 266237, China; University of Chinese Academy of Sciences, Beijing 100049, China; CAS Key Laboratory of Marine Ecology and Environmental Sciences, Institute of Oceanology, Chinese Academy of Sciences, Qingdao 266071, China; Laboratory for Marine Ecology and Environmental Science, Qingdao Marine Science and Technology Center, Qingdao 266237, China; CAS Key Laboratory of Marine Ecology and Environmental Sciences, Institute of Oceanology, Chinese Academy of Sciences, Qingdao 266071, China; Laboratory for Marine Ecology and Environmental Science, Qingdao Marine Science and Technology Center, Qingdao 266237, China

**Keywords:** littorinid, chromosomal assembly, intertidal adaptation, karyotype evolution

## Abstract

Living in the intertidal environment, littorinid snails are excellent models for understanding genetic mechanisms underlying adaptation to harsh fluctuating environments. Furthermore, the karyotypes of littorinid snails, with the same chromosome number as the presumed bilaterian ancestor, make them valuable for investigating karyotype evolution from the bilaterian ancestor to mollusks. Here, we generated high-quality, chromosome-scale genome assemblies for 2 littorinid marine snails, *Littorina brevicula* (927.94 Mb) and *Littoraria sinensis* (882.51 Mb), with contig N50 of 3.43 Mb and 2.31 Mb, respectively. Comparative genomic analyses identified 92 expanded gene families and 85 positively selected genes as potential candidates possibly associated with intertidal adaptation in the littorinid lineage, which were functionally enriched in stimulus responses, innate immunity, and apoptosis process regulation and might be involved in cellular homeostasis maintenance in stressful intertidal environments. Genome macrosynteny analyses indicated that 4 fissions and 4 fusions led to the evolution from the 17 presumed bilaterian ancestral chromosomes to the 17 littorinid chromosomes, implying that the littorinid snails have a highly conserved karyotype with the bilaterian ancestor. Based on the most parsimonious reconstruction of the common ancestral karyotype of scallops and littorinid snails, 3 chromosomal fissions and 1 chromosomal fusion from the bilaterian ancient linkage groups were shared by the bivalve scallop and gastropoda littorinid snails, indicating that the chromosome-scale ancient gene linkages were generally preserved in the mollusk genomes for over 500 million years. The highly conserved karyotype makes the littorinid snail genomes valuable resources for understanding early bilaterian evolution and biology.

## Introduction

Globally, widespread long-term environmental fluctuations result in constant changes to biotic and abiotic conditions (such as climate, nutrition loading, and habitat fragmentation), which act at different spatial scales and can profoundly impact the structure, function, and processes of ecosystems [1–3]. Living organisms that persist in fluctuating environments evolve the ability to tolerate physiological disturbances through a variety of physiological and behavioral responses that allow organisms to maintain homeostasis [4]. To better understand how they survive in and adapt to fluctuating environments, it is crucial to elucidate the mechanistic basis at a genetic level [4, 5].

Interfacing land and sea, rocky intertidal shores are the most common littoral habitats throughout the world [6]. Strongly influenced by both aquatic and terrestrial climatic regimes, the rocky intertidal zone is subject to steep environmental gradients, especially thermal and desiccation stresses that occur at low tide [1, 7, 8], which makes it a natural laboratory for examining relationships between abiotic stresses, biotic interaction, and ecological patterns in nature [9–11]. Species in intertidal habitats must adapt to 2 completely distinct environments because of the daily rhythm of the tides: submersion in the aquatic environment at high tide and emerging into the aerial environment at low tide [12]. From low to high shore levels, environmental pressures become more severe and last longer [7, 13].

The periwinkles or littorinids in the family Littorinidae (Children, 1834) are typical gastropoda organisms inhabiting intertidal environments, which contain at least 18 genera and 200 species [14, 15]. Given their wide distribution and high abundance in rocky intertidal shores with steep environmental gradients, littorinid snails have been established as a model system for studying adaptation, evolution, and speciation [16, 17]. Like those successful and well-known modern model species, the biology, taxonomy, phylogeny, and ecology of littorinid snails have been extensively studied [18–20], establishing a solid foundation for deeper investigation into speciation, sexual selection, and adaption to environmental change [21, 22]. *Littorina brevicula* (Philippi, 1844) (NCBI:txid45748; marinespecies.org:taxname:367,853) and *Littoraria sinensis* (Philippi, 1847) (NCBI:txid684704; marinespecies.org:taxname:446,915) are 2 common littorinid snails widely distributed in the rocky intertidal zone of the northwestern Pacific and are 2 of the most conspicuous and abundant gastropods in their habitats [23–25]. Regularly exposed to aquatic and desiccative environments due to daily rhythm of the tides, these 2 high-shore species are under the greatest abiotic stresses such as hyperthermy, desiccation, and hypoxia [6], while biotic stresses from pathogens like bacteria and viruses may also be severe due to herbivory of littorinid snails [26]. Understanding how these littorinid snails adapt to the fluctuating intertidal environments, especially thermal stresses, may be fundamental for understanding how species are likely to respond to climate change [21, 27]. Previous studies have discussed the mechanisms by which littorinid snails adapt to environmental challenges, for example, the tolerance limit of low and high temperatures of different littorinid populations and the molecular basis of intertidal adaption from both physiological and genetic aspects [12, 13, 27–29]. High-quality genomes are the base to facilitate littorinid snails to achieve their maximum potential as true ecological and evolutionary models [16]. However, there is only 1 publicly available high-quality chromosome-level littorinid genome for *Littorina saxatilis* [30]. By using PacBio CLR reads and Hi-C data, De Jode et al. [30] assembled a chromosome-level *L. saxatilis* genome spanning 1.35 Gb with a scaffold N50 of 67 Mb, which is much improved than the initial draft genome [31]. More high-quality genomes are still urgently needed for the evolutionary and ecological studies of littorinid snails.

Understanding how the enigmatic urbilateria, the last common ancestor of all bilaterians, was constructed is one of the key questions for evolutionary biology. Gastropods are among the oldest known bilaterians to appear in fossil records, and the earliest undisputed gastropods date from the Late Cambrian Period, around 500 million years ago [32]. The first unambiguous bilaterian fossil is Kimberella, dating to 555 million years ago, which shows remarkable resemblance to a mollusk [33]. Reconstructing the genome of the urbilaterian ancestors will shed light on our understanding of early bilaterian ancestors and their evolution [34]. Analysis of the evolution of karyotypes has been conducted extensively for bilaterian, metazoan, vertebrate, and so on [34–36]. Cytogenetic analyses and karyotype characterization confirm that the diploid chromosome number of 2n = 34 is common in littorinid snails [15, 37–39], which is the same with the presumed number of the ancient linkage groups (ALGs) of bilaterian ancestor [34], suggesting that the littorinid karyotype may represent the ancient karyotype of bilaterian ancestor to some extent. However, the evolutionary relationships between the 17 littorinid snail chromosomes and the 17 presumed ALGs of bilaterian ancestor are unclear, and the equal chromosome numbers do not necessarily imply 1:1 chromosomal homology. The 19 chromosomes of a bivalve mollusk, the scallop *Patinopecten yessoensis*, were confirmed to be highly conserved with the 17 bilaterian ALGs [34, 36, 40]. Macrosynteny analysis between *P. yessoensis* and littorinid genomes could provide insights into the karyotype evolution from the bilaterian ancestor to mollusks and evolution of early bilaterian ancestors.

In the present study, we assembled high-quality chromosome-level genomes for 2 littorinid snails, *Littorina brevicula* and *Littoraria sinensis*. Comparative genomic analyses were performed to investigate genetic mechanisms potentially associated with adaptation to intertidal harsh and fluctuating environments, which is one of many possible drivers of gene family and sequence evolution in the littorinid lineage. Macrosynteny analysis was also conducted to uncover karyotype evolution from the bilaterian ancestor to mollusks.

## Materials and Methods

### Sampling, genomic DNA extraction, and sequencing

Live specimens of *L. brevicula* and *L. sinensis* were collected from the rocky intertidal shore of Huiquan Bay in Qingdao (36°3′26″N,120°20′27″E) in 2019 and 2021, respectively, for genomic and transcriptomic sequencing. For genomic sequencing of *L. brevicula*, genomic DNA was extracted using the E.Z.N.A. Mollusc DNA Kit from foot muscle tissue of a single individual. Genomic DNA was sheared by a g-TUBE device (cat. 520,079; Covaris) and then repaired and purified for further PacBio CLR library preparation according to the manufacturer’s protocol (Pacific Biosciences). DNA fragments centered at ∼15 kb were extracted using BluePippin Systems from Sage Science. Sequencing was performed on the PacBio Sequel Ⅱ System (RRID:SCR_017990) with the Sequel Sequencing Kit 3.0 following the manufacturer’s instructions. Only subreads ≥5,000 bp were included for genome assembly.

For *L. sinensis*, genomic DNA from foot muscle tissue of a single snail was extracted using a Genomic-tip 100 G (QIANGEN) kit and sheared and size-selected with the aforementioned procedure for the SMRT library. ONT libraries were constructed with these selected fragments using the Ligation Sequencing 1D Kit (Oxford Nanopore, p/n SQK-LSK109) according to the manufacturer’s instructions. Sequencing was performed on the PromethION (ONT) platform. In order to polish the assembly, genomic DNA was extracted from the foot muscle tissue of the same individual using the E.Z.N.A. Mollusc DNA Kit, and short-read sequencing of a library with an insert length of ∼350 bp was performed on the DNBSEQ-T7 system (RRID:SCR_017981).

Genomic DNA was extracted from foot muscle tissue of another individual for both species using E.Z.N.A. Mollusc DNA Kit. Hi-C fragment libraries were constructed with insert size ranging from 300 to 700 bp and sequenced on the HiSeq X Ten (RRID:SCR_016385) and DNBSEQ-T7 system for *L. brevicula* and *L. sinensis*, respectively. Quality control was performed by HiC-Pro v2.8.1 (RRID:SCR_017643) [41]. All the sequencing was performed by the Biomarker Technologies Corporation.

Total RNA was extracted from foot muscle tissues of *L. brevicula* and *L. sinensis* using the TRIzol Kit (ThermoFisher Scientific Inc) for transcriptome sequencing. For the full-length transcriptome sequencing of *L. brevicula*, second-strand cDNA was synthesized using the SMARTer PCR cDNA Synthesis Kit. After PCR amplification, quality control, and purification, the products were then subjected to the construction of SMRTbell Template library using SMRTBell Template Prep kit, which was sequenced on the PacBio Sequel II platform. For the next-generation transcriptome sequencing of *L. brevicula* and *L. sinensis*, the library was inspected by Qsep-400 method after second-strand cDNA synthesis and PCR amplification, and the Hieff NGS Ultima Dual-mode mRNA Library Prep Kit was used for library construction. The libraries were then sequenced on the HiSeq X Ten and DNBSEQ-T7 system for *L. brevicula* and *L. sinensis*, respectively.

### Genome assembly and scaffolding

To assemble the genome of *L. brevicula*, subreads from PacBio sequencing were assembled using Wtdbg2 v2.5 (RRID:SCR_017225) [42] with the following parameters: “-x sq -g 1 g -X 100 -AS2 –node-len 2048 –aln-dovetail 20,480.” The resulting contigs were polished by GCpp v2.0.2 using PacBio data. Hi-C data were used to anchor contigs onto chromosomes using Juicer v1.6 (RRID:SCR_017226) [43] and 3D-DNA (RRID:SCR_017227) [44]. The chromosomal-level genome assembly was further adjusted using Juicebox v1.11.08 (RRID:SCR_021172) [45] and gap-filled with TGS-GapCloser v1.2.0 [46] and then polished again with GCpp v2.02.

The ONT long reads of *L. sinensis* were assembled using NextDenovo v2.4.0 (RRID:SCR_025033) with the following parameters: “read_cutoff = 1k, genome_size = 1g.” The assembly was first polished using PEPPER v0.1 (RRID:SCR_000431) with ONT long reads. Then, DNBSEQ-T7 short reads were aligned to the contigs, and single base errors were corrected by FREEBAYES v1.3.4 (RRID:SCR_010761) and PILON v1.2.3 [47]. The genome contigs were scaffolded into chromosomes with Hi-C reads using ALLHiC v0.9.8 (RRID:SCR_022750) [48]. The chromosomal-level genome assembly was further adjusted using Juicebox v1.11.08 [45] and gap-filled with TGS-GapCloser v1.2.0 (RRID:SCR_017633) [46] and then polished again with DNBSEQ-T7 reads.

To assess the genome quality, the completeness of the 2 genomes was assessed by BUSCO v5.2.1 (RRID:SCR_015008) [49] using the metazoan (metazoa_odb10) database, which contains 954 highly conserved single-copy core genes.

### Genome annotation

The repeat library was constructed by RepeatModeler v2.0.1 (RRID:SCR_015027) [50] and EDTA v2.0.1 (RRID:SCR_022063) [51] while RepeatMasker v4.1.2 (RRID:SCR_012954) [52] was used to identify and mask repetitive elements. Based on the repeat-masked genomes, protein-coding genes were predicted using a combination of 3 approaches: transcriptome-based, *de novo*, and homologue-based methods. First, transcripts from the foot muscle tissue of the 2 snails were assembled for transcriptome-based annotation. Illumina short reads of both littorinid snails and full-length PacBio Iso-Seq reads for *L. brevicula* were assembled using Trinity v2.11.0 (RRID:SCR_013048) [53] and ISOSEQ v3 (RRID:SCR_025481), respectively, and then mapped to the reference genome using MINIMAP2 v2.17 (RRID:SCR_018550) [54]. PASAPIPELINE v2.4.1 [55], STRINGTIE v2.2.1 [56], and TRANSDECODER v5.5.0 (RRID:SCR_017647) were used to predict candidate protein-coding regions. Second, *de novo* gene prediction was performed using AUGUSTUS 3.4.0 (RRID:SCR_008417) [57], BRAKER v2.1.6 (RRID:SCR_018964) [58], and GENEMARK v4.69 (RRID:SCR_011930) [59]. Third, METAEUK [60] was used for homologous gene annotation with protein sequences of the following 8 species: *Lottia gigantea, Haliotis discus hannai, Elysia chlorotica, Biomphalaria glabrata, Aplysia californica, Pomacea canaliculata, Octopus bimaculoides*, and *Octopus minor* (Supplementary Table S1). Finally, the results from the 3 approaches were integrated using EVidenceModeler v2.0.0 (RRID:SCR_014659) [61] and Funannotate v1.8.15 (RRID:SCR_023039). For the prediction of gene function, the predicted protein-coding genes were aligned to the databases of UniProt (RRID:SCR_002380) [62], Pfam-A [63], EggNOG [64], MEROPS [65], CAZYme [66], BUSCO [49], and InterProScan [67].

### Gene family, phylogenetic analysis, and divergence time estimation

Protein-coding sequences of *Argopecten purpuratus, Biomphalaria glabrata, Chlamys farreri, Chrysomallon squamiferum, Haliotis laevigata, Haliotis rubra, Nautilus pompilius, Patinopecten yessoensis, L. brevicula, L. sinensis*, and *Capitella teleta* (outgroup) (Supplementary Table S1) were aligned using DIAMOND v2.0.14.152 [68] with a cutoff e-value of 1e-5 and compared using OrthoFinder v2.5.5 (RRID:SCR_017118) [69] to construct gene families.

To infer the phylogenetic relationships, 829 single-copy gene families from all 11 species were extracted to perform multiple alignments using MAFFT v7.429 (RRID:SCR_011811) [70] with default parameter settings. After being transformed back to coding DNA and refined by using Gblocks v0.91b (RRID:SCR_015945) [71], all of the alignments were combined into a supergene. The phylogenetic tree was constructed based on the maximum likelihood method in IQ-TREE v1.6.12 (RRID:SCR_017254) [72] with the GTR+F+I+G4 model. Clade support was assessed using the bootstrapping algorithm with 1,000 replicates. The divergence time between each clade was estimated with MCMCTree in PAML v4.9 (RRID:SCR_014932) [73]. Three time calibration points were used to estimate the divergence times in the phylogenetic tree: the divergence time between *C. teleta* and molluscan species (534.3–654.0 million years ago [Mya]) [74], the divergence time between *N. pompilius* and Bivalvia and Gastropoda (527.6–619.1 Mya) [74], and the divergence time between *C. farreri* and *P. yessoensis* (46.1–71.7 Mya) [75].

### Expansion and contraction of gene families

The CAFE v5 tool (RRID:SCR_005983) [76] was used to examine gene family expansion and contraction with parameter “-p -k 1.” Based on a stochastic birth and death model with the lambda option [77], the size difference of each gene family was checked along each lineage of the phylogenetic tree. A probabilistic graphic model was applied to calculate the probability of transitions in gene family size from parent to child nodes. The corresponding *P* values were calculated for each lineage based on conditional likelihood. Gene families with a *P* value ≤0.05 were considered significantly expanded/contracted and were further subjected to Gene Ontology (GO) functional enrichment analyses using the topGO R package [78].

### Identification of positively selected genes

To identify genes under positive selection in the common ancestor of 2 littorinid snails (foreground branch), 4 submerged molluscan species (*A. purpuratus, C. farreri, P. yessoensis*, and *H. laevigata*) were used as background branches. These 4 species inhabit relatively stable sea bottoms and are vulnerable to environmental fluctuation. Single-copy orthologous gene families were extracted, and an unrooted phylogenetic tree was constructed using the methods mentioned above, based on which CODMEL of PAML package v4.9 [73] was used to identify genes under positive selection in the foreground branch using the branch-site model. FDR (False discovery rate) correction was performed on the results, and genes were identified as positively selected according to the adjusted *P* value (*P* < 0.01) and containing amino acid sites with a Bayesian and empirical Bayes approach higher than 99%.

### Macrosynteny analyses

Chromosome-scale synteny analyses were performed pairwisely for *L. brevicula, L. sinensis*, and *P. yessoensis*. Protein sequences of single-copy gene families were aligned to each other using DIAMOND v2.0.14.152 with parameter “-k1.” The macrosynteny analyses were conducted using the MCScanX (RRID:SCR_022067) [79] package with defaulting parameters. The results were then visualized into dot plot figures using the VGSC Java package.

## Results

### Genome assembly and annotation for 2 littorinid snails

The PacBio CLR sequencing and ONT sequencing generated a total of 197.66 Gb (∼212-fold coverage) and 16.58 Gb (∼20-fold coverage) clean data for *L. brevicula* and *L. sinensis*, respectively. The accuracy of ONT long reads was estimated with Phred quality scores (Q20 = 74.96% and Q30 = 74.95%). The DNBSEQ-T7 system generated 145 G (∼176-fold coverage) clean short reads for the assembly polish of *L. sinensis*. To construct chromosome-level genome assemblies, 129.31 Gb (∼138×) and 114.67 Gb (∼123×) clean Hi-C reads were obtained for *L. brevicula* and *L. sinensis*, with 92.21% and 99.98% assembled sequences of *L. brevicula* and *L. sinensis* anchored onto 17 pseudochromosomes (Supplementary Table S2), which is consistent with previous karyotype analysis [15]. The full-length transcriptome sequencing on the PacBio platform generated ∼49.5 G clean data for *L. brevicula* while the transcriptome sequencing on the HiSeq X Ten and DNBSEQ-T7 system generated ∼9.5 G and ∼13.9 G clean data for *L. brevicula* and *L. sinensis*, respectively. Finally, chromosome-level genome assemblies spanning 927.94 Mb for *L. brevicula* and 822.51 Mb for *L. sinensis* were obtained, with contig N50 of 3.43 Mb and 2.31 Mb (Table 1). The BUSCO results indicated high genome assembly completeness, with 888 (93.1%) and 894 (93.8%) out of 954 metazoan single-copy core genes present in the genome assemblies of *L. brevicula* and *L. sinensis* (Supplementary Table S3).

**Table 1:** Summary of statistics for the *L. brevicula* and *L. sinensis* genome assembly

	*L. brevicula*	*L. sinensis*
Genome scaffold total	3,132	27
Genome contig total	3,702	935
Genome scaffold sequence total	928.20 Mb	822.61 Mb
Genome contig sequence total	927.93 Mb (0.029% gap)	822.51 Mb (0.011% gap)
Genome contig N50	3.43 Mb	2.31 Mb
Genome scaffold N50	48.134 Mb	32.91 Mb

Repetitive elements composed 47.25% (438.52 Mb) and 41.09% (337.97 Mb) of the genome for *L. brevicula* and *L. sinensis*, respectively (Table 2). A total of 29,335 and 25,386 genes were predicted for *L. brevicula* and *L. sinensis*. The gene number, gene length, coding sequence (CDS) number, and lengths of CDS, introns, and exons are described in Supplementary Tables S5 and S6. A total of 25,495 (86.91%) and 23,238 (91.54%) genes were functionally annotated for *L. brevicula* and *L. sinensis*.

**Table 2:** Classification of the repetitive elements in *L. brevicula* and *L. sinensis* genome assembly

	Count		Length (bp)		% of genome	
Type	*L. brevicula*	*L. sinensis*	*L. brevicula*	*L. sinensis*	*L. brevicula*	*L. sinensis*
DNA transposons	1,590,204	684,216	204,405,206	98,112,746	22.02	11.93
Retroelements	346,793	302,792	82,326,206	77,246,672	8.87	9.39
Other	1,049,612	1,286,201	91,614,177	119,303,375	9.87	14.5
Unknown	306,488	183,893	60,252,340	43,309,094	6.49	5.27
Total	3,293,097	2,457,102	438,527,929	337,971,887	47.25	41.09

### Gene family, phylogenetic, and divergence analyses

A total of 267,386 (89.99%) genes were assigned to 29,488 orthologous groups, of which 5,017 were shared among all 11 species and 2,950 were specific to the 2 littorinid snails (Supplementary Table S7). Functional annotation and GO enrichment analysis showed that these littorinid-specific gene families were involved in 142 GO terms relevant to metabolic processes, antioxidant responses, innate immunity, and so on.

**Figure 1:**
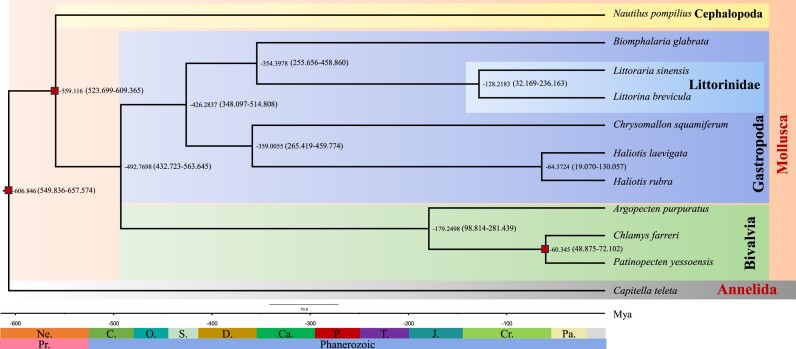
Maximum likelihood phylogenetic tree constructed by MCMCTree with divergence time estimated among species. Numbers next to the nodes represent the estimated divergence time (million years ago [Ma]). Divergences used for the recalibration of time estimation are indicated with red squares. The credibility intervals with 95% HPD (Highest Posterior Density) of the divergence time are shown in the parentheses.

Based on 829 single-copy gene families, the species tree for 10 mollusks was constructed using *C. teleta* as the outgroup (Fig. 1). The divergence time between *L. brevicula* and *L. sinensis* was estimated to be ∼128.2 million years, suggesting a deep divergence between the 2 littorinid snails, yet they shared highly conserved macrosynteny (see below).

### Expanded gene families and positively selected genes

A total of 92 significantly expanded gene families (involving 897 genes) and 15 contracted gene families (involving 11 genes) were identified for the common littorinid ancestor of *L. brevicula* and *L. sinensis*. The significantly expanded gene families were mainly involved in innate immunity, metabolic processes, stimulus responses, antioxidant responses, and so on. For example, carbohydrate hydrolase and triglyceride-related gene families related to energy metabolism, gene families encoding cytochrome P450 (CYP450) and glutathione S-transferases (GSTs) with known functions in constituting the xenobiotic detoxification system of mollusks [80], and defense gene sets like HEPN domain-containing proteins and Sacsin, which contained Hsp90-like domains and recruited Hsp70 [81], were found to be expanded in the littorinid lineage and might facilitate adaptation to harsh intertidal environments. Moreover, gene families encoding multiple pattern recognition receptors (PRRs) expanded the most among all the 92 littorinid expanded gene families, which contained C-type lectin-related proteins (CREPs), fibrinogen-related proteins (FREPs), scavenger receptor cysteine-rich proteins (SRCRs), G-protein coupled receptors (GPCRs), and so on. These PRRs constructed the innate immune system of littorinid snails and might play important roles in pathogen defense.

A total of 501 positively selected genes were identified (*P* ≤ 0.01) for the common ancestor of *L. brevicula* and *L. sinensis*. The functions of these genes were annotated using databases mentioned earlier and further confirmed using the GeneCards database [82]. Compared to the littorinid-specific and expanded gene families, functions of the positively selected genes were more unified, which were mainly associated with nucleotide or protein binding.

### Evolution of littorinid chromosomes from the ancient bilaterian ancestor

**Figure 2:**
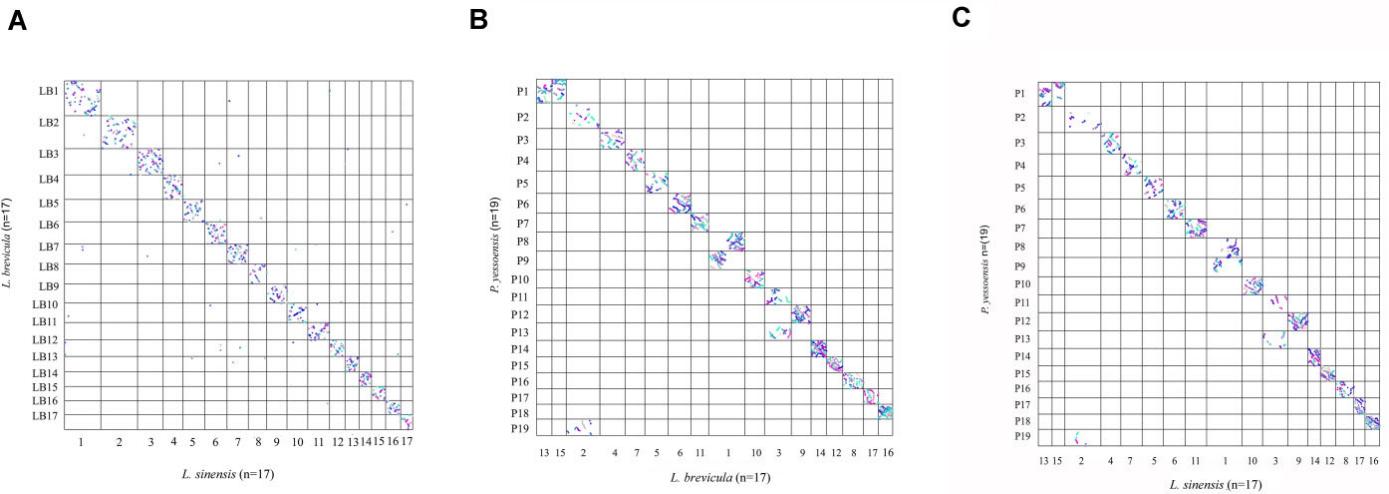
Dot plot of genome macrosynteny between littorinids and *P. yessoensis* chromosomes. Each dot represents a common single-copy gene.

Genome macrosynteny analyses independent of intrachromosomal rearrangements were performed pairwisely among the 2 littorinid snails and the scallop *P. yessoensis* using orthologous single-copy genes. The results showed a near-perfect correspondence between chromosomes of *L. brevicula* and *L. sinensis* with few interchromosomal rearrangements (Fig. 2A). Meanwhile, the correspondence between littorinids and the scallop indicated that *P. yessoensis* chromosomes PY8 and PY9 were homologous to littorinid chromosome L1, PY2 and PY19 were homologous to L2, PY11 and PY13 were homologous to L3, and PY1 was homologous to L13 and L15, resulting in the difference of chromosome numbers between littorinid snails (*n* = 17) and *P. yessoensis* (*n* = 19) (Fig. 2B, C).

## Discussion

As candidate ecological and evolutionary models, high-quality genomes are urgently needed for littorinid snails. Considering the higher accuracy of the PacBio platform than the ONT platform, long-read sequencing of the 2 littorinid snails was performed by using the PacBio platform at the beginning. However, PacBio sequencing for *L. sinensis* failed possibly due to mucopolysaccharides that might block the zero-model waveguides (ZMWs). Genomic library construction and sequencing of *L. sinensis* was then performed by multiple flow cells of the ONT platform from which adequate sequencing data were finally generated. Here, we generated chromosome-scale genome assemblies for 2 littorinid marine snails. Assessment and comparation with other published molluscan genomes showed a high level of continuity and completeness but a moderate level of size and repetitive elements for the 2 littorinid genomes (Supplementary Table S4), which ensure the accuracy of comparative genomic analyses in our study and provide qualified genomic materials for further molecular ecology and evolution researches.

The fossil record of littorinid snails is incomplete because of poor conditions for preservation on intertidal rocky shores [14, 83, 84], which leads to difficulty in time calibration for the divergence time estimation. In the present study, the estimated divergence time of these 2 littorinid snails was about 128.22 My. According to Williams et al. [14], the estimated age of Littorininae is at least Lower Cretaceous (115–190 Mya). Therefore, the split of the genera *Littorina* and *Littoraria* might have happened not long after the origin of Littorininae. However, the estimated divergence time between the 2 littorinid snails was larger than that in Reid et al. [83] (90–95 Mya) based on combined phylogenetic analysis of 28S rRNA, 12S rRNA, and cytochrome oxidase c subunit I genes. The accuracy of phylogenetic divergence time estimation based on the whole-genome data was supposed to be higher than fragmented sequences of several loci. However, it has also been suggested that there is methodological bias toward overestimation of time based on molecular divergence [85]. So, more littorinid reference genomes are still needed for accurate divergence time estimation among different littorinid genera.

The intertidal rocky shores are characterized with multiple biotic and abiotic environmental stresses that affect the cellular homeostasis of living organisms. Comparative genomic analysis indicated possible genetic adaptation strategies of littorinid snails to the intertidal environment. The expanded energy metabolism gene families, which may facilitate organisms generating ATP to compensate for extra energy demands, are known as key factors in establishing limits of environmental stress tolerance [86]. Genes like CYP450 and GSTs might help littorinid snails to withstand pollutants by detecting and binding with organic and inorganic toxicants [80]. The expanded genes associated with innate immunity might play a key role in adaptation to severe biotic stresses (e.g., virus, bacteria, and parasites) by recognizing and eliminating pathogen through phagocytosis [87]. A total of 85 positively selected genes were identified (Supplementary Table S8) as potential candidates for intertidal adaptation and almost half of which were related to nucleotide and protein-binding processes and involved in damaged DNA/RNA/protein repairment or degradation. These results suggested that maintenance of cellular homeostasis and repair of damaged nucleotides and proteins might be essential to hinder cell apoptosis processes caused by environmental stresses, which could help littorinid snails to adapt to or even thrive in the harsh intertidal environment. However, changes on the littorinid branch might have been driven by factors other than adaptation to the intertidal environment, considering the long branch of the littorinid lineage on the phylogenetic tree. Besides, annotations based on the public database sometimes failed to provide solid and direct evidence for the gene function. Therefore, to elucidate the genetic mechanism for intertidal adaptation of littorinid snails, more molluscan genomes closely related to littorinid snails are needed for comparison, together with functional assay experiments of these candidate genes/gene families.

**Figure 3:**
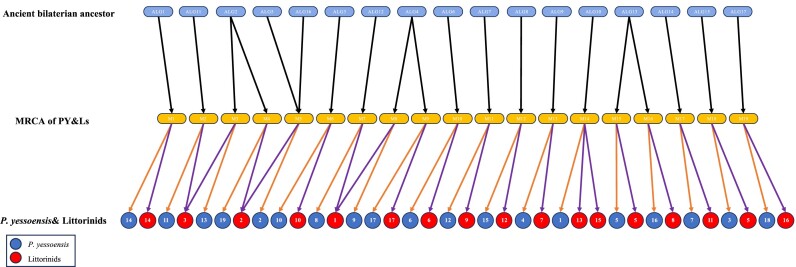
Chromosome macrosynteny of the presumed ancient bilaterian ancestor (ALG), the most parsimonious karyotype reconstruction of the common ancestor of *P. yessoensis* and the littorinids (MRCA of PY&Ls), *P. yessoensis*, and littorinid linkage groups. Chromosomes of *P. yessoensis* are shown by blue circles while those of littorinid snails are shown by red circles.

Previous macrosynteny analyses revealed that *P. yessoensis* possessed a highly conserved 19-chromosome karyotype similar to that of bilaterian ancestors [36, 40] and the 19 scallop chromosomes evolved from the 17 presumed ALGs of bilaterian ancestors through 3 chromosomal fissions (ALG13 to PY5 and PY16, ALG4 to PY9 and PY17, ALG2 to PY13 and PY19) and 1 fusion (ALG5 and ALG16 to PY2). Therefore, the evolutionary trajectory from the 17 ALGs of bilaterian ancestors to the 17 littorinid chromosomes can be inferred based on the macrosynteny analyses: (i) ALG2 fissioned into ALG2-1 and ALG2-2, as well as ALG4 fissioned into ALG4-1 and ALG4-2; (ii) ALG2-1 fused with ALG5 and ALG16 into L2, as well as ALG2-2 fused with ALG11 into L3 and ALG4-1 fused with ALG12 into L1; and (iii) ALG13 fissioned into L5 and L8, as well as ALG10 fissioned into L13 and L15, which indicated that the 17 chromosomes of littorinids evolved from the 17 ALGs of bilaterian ancestors through 4 chromosomal fusions and 4 fissions regardless of intrachromosomal rearrangements (Fig. 3).

Although the 17 littorinid chromosomes did not possess a complete “1-to-1” conserved model with the 17 presumed bilaterian ALGs, our analyses revealed that most littorinid chromosomes (9) directly inherited ancient bilaterian gene linkages while the other 8 chromosomes evolved from 4 chromosomal fissions and 4 fusions (Fig. 3). Although different possible ancestral karyotypes for the common ancestor of *P. yessoensis* and littorinids can be reconstructed, the most parsimonious ancestral karyotype was the same as that of *P. yessoensis* (Fig. 3), considering the smallest number of chromosomal mutations from the bilaterian ancestors to *P. yessoensis* and littorinids. Surprisingly, all of the 3 chromosomal fissions and 1 chromosomal fusion between the bilaterian ancestors and *P. yessoensis* were also found between littorinid snails and the bilaterian ancestors, which, based on the most parsimonious ancestral state reconstructed, implied that they might have occurred before the bivalve–gastropod split around 500 million years ago. Overall, the level of chromosome preservation was comparable for the scallop lineage and the littorinid lineage. Considering the sister relationship between Bivalvia and Gastropoda, these results demonstrated that the chromosome-scale ancient gene linkages were generally preserved in the mollusk genomes over 500 million years, which added evidence to the conclusion that slow chromosome evolution was widespread among invertebrates [35]. Wang et al. [40] proposed that the remarkable conservation of ancestral features in scallop genome is probably as a consequence of life on cold and stable deep-ocean bottoms. However, although the littorinid snails live in the harsh and highly fluctuating intertidal environments, they still have high level of chromosome preservation with the bilaterian ancestors, which is similar to that of scallop. The results implied that living environments might not be the key driver of karyotype evolution in mollusks, and other evolutionary or developmental constraints on the evolution of genome organization could exist.

## Supplementary Material

giae072_GIGA-D-24-00090_Original_Submission

giae072_GIGA-D-24-00090_Revision_1

giae072_GIGA-D-24-00090_Revision_2

giae072_Response_to_Reviewer_Comments_Original_Submission

giae072_Response_to_Reviewer_Comments_Revision_1

giae072_Reviewer_1_Report_Original_Submission Roger Butlin -- 4/18/2024 Reviewed

giae072_Reviewer_1_Report_Revision_1 Roger Butlin -- 6/27/2024 Reviewed

giae072_Reviewer_2_Report_Original_Submission Rafael Zardoya, PhD -- 4/21/2024 Reviewed

giae072_Reviewer_2_Report_Revision_1 Rafael Zardoya, PhD -- 7/20/2024 Reviewed

giae072_Supplemental_File

## Data Availability

The sequencing data that support the findings of this study are openly available in the NCBI Sequence Read Archive (SRA) under BioProject accession number PRJNA1032305 (*Littorina brevicula*) and PRJNA1032307 (*Littoraria sinensis*). The genome assembly and annotation data of *L. sinensis* [88] and *L. brevicula* [89] and all additional supporting data have been deposited in the *GigaScience* repository, GigaDB [90].

## Additional Files

**Supplementary Table S1**. Metazoan genome assemblies and gene models used in this study.

**Supplementary Table S2**. Statistics of chromosomal-level assembly of the 2 littorinid snails.

**Supplementary Table S3**. Completeness assessment of the 2 littorinid snails by BUSCO.

**Supplementary Table S4**. Genome size and repetitive elements of 46 mollusks.

**Supplementary Table S5**. Statistics of predicted protein-coding genes in the genome assembly of *L. brevicula*.

**Supplementary Table S6**. Statistics of predicted protein-coding genes in the genome assembly of *L. sinensis*.

**Supplementary Table S7**. Statistics of gene families of 11 species in comparative genomic analysis.

**Supplementary Table S8**. Potential candidate intertidal adaptation-related genes under positive selection.

## Abbreviations

ALG: ancient linkage group; bp: base pairs; BUSCO: Benchmarking Universal Single-Copy Orthologs; FDR: False Discovery Rate; Gb: gigabase pairs; GO: Gene Ontology; HEPN: higher eukaryotic and prokaryotic nucleasedomains; HPD: Highest Posterior Density; HSP: heat shock protein; HiC: high-throughput/resolution chromosome conformation capture; kb: kilobase pairs; Mb: megabase pairs; Mya: million years ago; NCBI: National Center for Biotechnology Information; ONT: Oxford Nanopore Technologies; PacBio: Pacific Biosciences.
