# Supplementary material for: Chromosome-level genome assemblies of two littorinid marine snails indicate genetic basis of intertidal adaptation and ancient karyotype evolved from bilaterian ancestors
Source: Gigascience. 2024 Sep 25;13:giae072. doi: 10.1093/gigascience/giae072 (PMC11423352; doi:10.1093/gigascience/giae072)
Supplement: giae072_GIGA-D-24-00090_Original_Submission [file giae072_giga-d-24-00090_original_submission.pdf]

GigaScience

Chromosome-level genome assemblies of two littorinid marine snails reveal the genetic basis of intertidal adaptation and ancient karyotype evolved from bilaterian ancestors

--Manuscript Draft--

**Manuscript Number:**	GIGA-D-24-00090
**Full Title:**	Chromosome-level genome assemblies of two littorinid marine snails reveal the genetic basis of intertidal adaptation and ancient karyotype evolved from bilaterian ancestors
**Article Type:**	Research
**Funding Information:**	National Natural Science Foundation of China (31970488) — Dr. Jin-xian Liu<br>National Natural Science Foundation of China (31972793) — Dr. Dong-Xiu Xue
**Abstract:**	Understanding the evolutionary adaptation to harsh fluctuating environments and early evolution of bilaterians is of great importance in evolutionary biology. Intertidal mollusks are excellent models for addressing these questions. Here, we generated high-quality, chromosome-scale genome assemblies for two littorinid marine snails, Littorina brevicula (927.94Mb) and Littoraria sinensis (882.51Mb) with contig N50 of 3.43Mb and 2.31Mb, respectively. Comparative genomic analyses revealed strong adaptive genomic signals for evolutionary adaptation to the harsh fluctuating intertidal environments. Gene families involved in activate stimulus responses, metabolic processes, innate immunity, antioxidant response and regulate apoptosis processes played key roles in adaptation to multiple biotic and abiotic stresses in intertidal environments. Positively selected genes associated with damaged protein/nucleotide repair/elimination might play important roles in cellular homeostasis maintenance in stressful intertidal environments. Genome macrosynteny analyses indicated that 4 fissions and 4 fusions led to the evolution from the 17 presumed bilaterian ancestral chromosomes to the 17 littorinid chromosomes, implying that the littorinid snails have a highly conserved karyotype with the bilaterian ancestor. Three chromosomal fissions and 1 chromosomal fusion from the bilaterian ALGs were shared by the bivalve scallop and gastropoda littorinid snails, indicating that the chromosome-scale ancient gene linkages were generally preserved in the mollusk genomes for over 500 million years. We propose that, other than the stability of living environments, other evolutionary or developmental constraints could exist on the evolution of genome organization of early bilaterians. The highly conserved karyotype makes the littorinid snail genomes valuable resources for understanding early bilaterian evolution and biology.
**Corresponding Author:**	Jin-xian Liu<br>Institute of Oceanology Chinese Academy of Sciences<br>Qingdao, CHINA
**Corresponding Author Secondary Information:**	
**Corresponding Author's Institution:**	Institute of Oceanology Chinese Academy of Sciences
**Corresponding Author's Secondary Institution:**	
**First Author:**	Yan-Shu Wang
**First Author Secondary Information:**	
**Order of Authors:**	Yan-Shu Wang
	Meng-Yu Li
	Yu-Long Li
	Yu-Qiang Li
	Dong-Xiu Xue
	Jin-xian Liu

Powered by Editorial Manager® and ProduXion Manager® from Aries Systems Corporation

Order of Authors Secondary Information:	
Additional Information:	
Question	Response
Are you submitting this manuscript to a special series or article collection?	No
**Experimental design and statistics**<br><br>Full details of the experimental design and statistical methods used should be given in the Methods section, as detailed in our Minimum Standards Reporting Checklist. Information essential to interpreting the data presented should be made available in the figure legends.<br><br>Have you included all the information requested in your manuscript?	Yes
**Resources**<br><br>A description of all resources used, including antibodies, cell lines, animals and software tools, with enough information to allow them to be uniquely identified, should be included in the Methods section. Authors are strongly encouraged to cite Research Resource Identifiers (RRIDs) for antibodies, model organisms and tools, where possible.<br><br>Have you included the information requested as detailed in our Minimum Standards Reporting Checklist?	Yes
**Availability of data and materials**<br><br>All datasets and code on which the conclusions of the paper rely must be either included in your submission or deposited in publicly available repositories (where available and ethically appropriate), referencing such data using a unique identifier in the references and in the "Availability of Data and Materials" section of your manuscript.	Yes

Have you have met the above requirement as detailed in our Minimum Standards Reporting Checklist?	

Manuscript Click here to access/download;Manuscript;renamed_232bf.docx

Chromosome-level genome assemblies of two littorinid marine snails reveal the genetic basis of intertidal adaptation and ancient karyotype evolved from bilaterian ancestors

Yan-Shu Wang[a,b,c,1], Meng-Yu Li[a,b,c,1], Yu-Long Li[a,b,1], Yu-Qiang Li [a,b,c], Dong-Xiu Xue[a,b], Jin-Xian Liu[a,b,2]

[a] CAS Key Laboratory of Marine Ecology and Environmental Sciences, Institute of Oceanology, Chinese Academy of Sciences, Qingdao 266071, China; [b] Laboratory for Marine Ecology and Environmental Science, Qingdao Marine Science and Technology Center, Qingdao 266237, China; [c] University of Chinese Academy of Sciences, Beijing 100049, China.

[1] These authors contributed equally to this work.

[2] To whom correspondence should be addressed.

Email: Jin-Xian Liu jinxianliu@gmail.com

Abstract

Understanding the evolutionary adaptation to harsh fluctuating environments and early evolution of bilaterians is of great importance in evolutionary biology. Intertidal mollusks are excellent models for addressing these questions. Here, we generated high-quality, chromosome-scale genome assemblies for two littorinid marine snails, *Littorina brevicula* (927.94Mb) and *Littoraria sinensis* (882.51Mb) with contig N50 of 3.43Mb and 2.31Mb, respectively. Comparative genomic analyses revealed strong adaptive genomic signals for evolutionary adaptation to the harsh fluctuating intertidal environments. Gene families involved in activate stimulus responses, metabolic processes, innate immunity, antioxidant response and regulate apoptosis processes played key roles in adaptation to multiple biotic and abiotic stresses in intertidal environments. Positively selected genes associated with damaged protein/nucleotide repair/elimination might play important roles in cellular homeostasis maintenance in stressful intertidal environments. Genome macrosynteny analyses indicated that 4 fissions and 4 fusions led to the evolution from the 17 presumed bilaterian ancestral chromosomes to the 17 littorinid chromosomes, implying that the littorinid snails have a highly conserved karyotype with the bilaterian ancestor. Three chromosomal fissions and 1 chromosomal fusion from the bilaterian ALGs were shared by the bivalve scallop and gastropoda littorinid snails, indicating that the chromosome-scale ancient gene linkages were generally preserved in the mollusk genomes for over 500 million years. We propose that, other than the stability of living environments, other evolutionary or developmental constraints could exist on the evolution of genome organization of early bilaterians. The highly conserved karyotype makes the littorinid snail genomes valuable

resources for understanding early bilaterian evolution and biology.

Keywords: littorinid, chromosomal assembly, intertidal adaptation, karyotype evolution

Introduction

Globally widespread long-term environmental fluctuations result in constant changes to biotic and abiotic conditions (such as climate, nutrition loading, and habitat fragmentation), which act at different spatial scales and can profoundly impact the structure, function, and process of ecosystems [1-3]. Living organisms that persist in fluctuating environments acquire the ability to tolerate physiological disturbances through a variety of physiological and behavioral responses that allow organisms to maintain homeostasis [4]. The genetic mechanisms that facilitate the capacity for adaptation to fluctuating environments, which are crucial for understanding or predicting how species will respond to constant global changes, remain mostly unknown.

Interfacing the land and the sea, rocky intertidal shores are the most common littoral habitats throughout the world [5]. Strongly influenced by both aquatic and terrestrial climatic regimes, the rocky intertidal zone is subject to steep environmental gradients, especially thermal and desiccation stresses that occur at low tide [1, 6, 7], which makes it a natural laboratory for examining relationships between abiotic stresses, biotic interaction and ecological patterns in nature [8-10]. Species in intertidal habitats must adapt to two completely distinct environments because of the daily rhyme of the tides: submersion in the aquatic environment at high tide and emerging into the aerial environment at low tide [11]. From low to high shore levels,

environmental pressures become more severe and last longer [6, 12].

Intertidal invertebrates are ectotherms of marine origins but are well adapted to terrestrial conditions during each low tide [7]. With 7% of all species on Earth, Mollusca is the second-largest animal phylum [13], which comprises many species of evolutionary, ecological, and economic interest [14-16]. Like coastal marine invertebrates, most aquatic mollusks have a pelagic larval phase but a sedentary adult phase [17], which means that they spend most of their lives within limited areas and cannot avoid environmental stresses by changing habitats. Gastropods are the most diverse and highly successful class of mollusks, occupying arguably the widest range of habitats of all metazoan taxa, ranging from deep-sea vents to the alpine region and from deserts to polar regions [18, 19]. Their capacity to survive in harsh environments indicates that they have evolved unique adaptations to multiple stresses, and some species become excellent models for researching rapid response and adaptation to environmental changes. One of the most typical models is the marine gastropod mollusks in the family Littorinidae (Children, 1834), the periwinkles or littorinids, which contains at least 18 genera and 200 species [20, 21]. Given their wide distribution and high abundance in rocky intertidal shores with steep environmental gradients, littorinid snails have been established as a model system for studying adaptation, evolution, and speciation [22, 23]. Like those successful and well-known modern model species, the biology, taxonomy, phylogeny, and ecology of littorinid snails have been extensively studied [24-26], establishing a solid foundation for deeper investigation in speciation, sexual selection and adaption to environmental change [27]. *Littorina brevicula* (Philippi, 1844) and *Littoraria sinensis* (Philippi, 1847) are two common littorinid snails widely distributed in the rocky intertidal zone of the northwestern Pacific and

are two of the most conspicuous and abundant gastropods in their habitats [28-31]. Regularly exposed to aquatic and desiccative environments due to the daily rhyme of the tides, these two high-shore species are under the greatest abiotic stresses such as hyperthermy, desiccation, and hypoxia, while biotic stresses from pathogens like bacteria and viruses are also severe [17]. Understanding how these littorinid snails respond to fluctuating intertidal environments is fundamental for understanding how species are likely to respond to climate change. Previous studies have discussed the mechanisms by which littorinid snails adapt to environmental challenges, for example, the tolerance limit of low and high temperature of different littorinid populations and the molecular basis of intertidal adaption from both physiological and genetic aspects [11, 12, 32-34]. However, to facilitate littorinid snails to achieve their maximum potential as true ecological and evolutionary models, high-quality genomes are urgently needed [22], which is devoid currently. In addition, with an accessible and accurate reference genome, comparative genomics combined with phylogenomic approaches may provide information about lineage-specific adaptations in an evolutionary context [35].

High-throughput sequencing revolution has rapidly improved accuracy meanwhile reducing the cost and leading to the popularization of whole-genome sequencing and reference genome assembly for non-model species [36, 37]. Despite the critical role that mollusks play in economy, ecology, and evolution, the available genome resources of Mollusca species is still limited [38]. Molluscan long-read genome sequencing and assembly are challenging because high-throughput library preparation and sequencing depend on DNA with high molecular weight, quantity, and purity [39, 40]. As for littorinid snails, the obstacles to the extraction of high molecular weight DNA with high purity have always been the main cause of the lack of

high-quality genome assemblies. On one hand, the major challenge is that high content of mucopolysaccharides that tend to be co-purified with DNA will inhibit downstream DNA library preparations [41, 42]. On the other hand, higher amounts and purity genomic DNA is needed for littorinid genome assembly whereas some littorinid snails are too small to extract adequate amounts of DNA from a single individual [38]. An accessible, accurate and qualified reference genome is the foundation to understand the genomic basis and molecular mechanisms of adaptation in a model species for evolution and ecology, yet no high-quality reference genome for littorinid snails has been successfully sequenced and assembled [27]. For the assembly of the draft genome of *Littorina saxatilis*, short-read sequencing alone was unable to produce an accurate assembly for such a large, heterozygous and highly repetitive genome. A combination of several Illumina sequencing datasets with insert sizes ranging from ~110bp to ~5,900bp was used for contig assembly and initial scaffolding of *L. saxatilis* genome, which was further gap-filled and extended using PacBio long reads. The resulting draft *L. saxatilis* genome spans ~1.35Gb with a contig N50 of 44.28Kb without genome annotation, which was much improved from earlier efforts yet still has some way to go [27, 43]. Therefore, a high-quality genome with credible annotation information for littorinid snails is extremely urgent for evolutionary and ecological studies.

Understanding how the enigmatic urbilateria, the last common ancestor of all bilaterians, was constructed is one of the key questions of the Evo-Devo field. Gastropods are amongst the oldest known bilaterians to appear in fossil records and the earliest undisputed gastropods date from the Late Cambrian Period, around 500 million years ago [44]. Many gastropods alive today are almost unchanged from their ancestors over several hundred million years. The first

unambiguous bilaterian fossil is Kimberella, dating to 555 million years ago, which shows remarkable resemblance to a mollusk [45]. Thus, the gastropod mollusks may provide an excellent model for the study of evolution for early bilaterians ancestor. Reconstructing the genome of the urbilaterian ancestors will shed light on our understanding of early bilaterian ancestors and their evolution [46]. The karyotype, especially chromosome number, has always been a basic genetic characteristic of a taxon and used as an informative phylogenetic indicator [47, 48]. Different karyotypes within phylogenies reflect karyotype evolution, which has been thought to be a major driver of speciation and divergent adaptation of species clades [49-51]. Analysis of the evolution of karyotypes has been conducted extensively for bilaterian, metazoan, vertebrate, etc. [46, 52, 53] Cytogenetic analyses and karyotype characterization confirm that the diploid chromosome number of 2n = 34 is common in littorinid snails [21, 54-56], which is the same with the presumed number of the ancient linkage groups (ALGs) of bilaterian ancestor [46], suggesting that the littorinid karyotype may represent the ancient karyotype of bilaterian ancestor to some extent. However, the evolutionary relationships between the 17 littorinid snail chromosomes and the 17 presumed ALGs of bilaterian ancestor are unclear, and the equal chromosome numbers do not necessarily imply 1:1 chromosomal homology. The 19 chromosomes of a bivalve mollusk, the scallop *Patinopecten yessoensis* were confirmed to be highly conserved with the 17 bilaterian ALGs [46, 53, 57]. Macrosynteny analysis between *Patinopecten yessoensis* and littorinid genomes should give insights into the karyotype evolution from the bilaterian ancestor to mollusks, and evolution of early bilaterian ancestors.

In the present study, we assembled high-quality chromosome-level genomes for two littorinid snails, *Littorina brevicula* and *Littoraria sinensis*. Comparative genomic analyses

were performed to uncover genetic mechanisms associated with adaptation to intertidal harsh and fluctuating environments and karyotype evolution. We found gene families related to stimulus responses, metabolic processes, innate immunity, and antioxidant response might be associated with the adaptation to severe fluctuating intertidal environments. Positively selected genes associated with damaged protein/nucleotide repair/elimination might play key roles in cellular homeostasis maintenance. A highly conserved bilaterian ancestor-like karyotypes were found in both littorinid snails, suggesting that the littorinid snails have a karyotype close to that of the bilaterian ancestor. Thus, the gastropod mollusks may provide an excellent model for studying the evolution of early bilaterians ancestor. These two high-quality and well-annotated reference genomes, serving as the first chromosome-level whole-genome resources of littorinids, can also provide the basis for further ecological and evolutionary research of this important group.

Material and Methods

Sampling, genomic DNA extraction and sequencing

Littorina brevicula and *Littoraria sinensis* individuals were collected from the rocky intertidal shore of Huiquan Bay in Qingdao (36°3′26″N,120°20′27″E) in 2019 and 2021 respectively. We tried different DNA extraction and genome sequencing strategies for the two snails to generate qualified data for the third-generation genome sequencing. Genomic DNA was first extracted from snail foot muscle tissues by the standard phenol chloroform extracting

method only to find that adaptors failed to be added to DNA fragments. Whole genomic DNA was then extracted by using CTAB method and several DNA extraction kits and qualified genomic DNA was finally obtained using the E.Z.N.A Mollusc DNA Kit (OMEGA bio-tek) and Genomic-tip 100G (QIANGEN) kit. DNA library conduction and sequencing of the two littorinid snails was both performed on the PacBio platform firstly considering the higher accuracy compared with the ONT platform while data generated from DNA sequencing of *L. sinensis* was scarce probably caused by mucopolysaccharides which might block the zero-model waveguides (ZMWs). DNA library conduction and sequencing of *L. sinensis* was then performed by multiple flow cells of the ONT platform from which adequate sequencing data was finally generated. Details of genome sequencing of the two littorinid snails was described as follows.

For the genomic sequencing of *L. brevicula*, genomic DNA was extracted using the E.Z.N.A Mollusc DNA Kit for genome assembly and correction. Genomic DNA of a Qingdao *L. brevicula* individual was sheared by a g-TUBE device (Catalog No. 520079, Covaris, MA) and then repaired and purified for further single-molecular real-time (SMRT) library preparation according to the manufacturer's protocol (Pacific Biosciences, CA). DNA fragments centered at ~15kb were extracted using BluePippin Systems from Sage Science. Sequencing was performed on the PacBio Sequel Ⅱ System with the Sequel Sequencing Kit 3.0 chemistry following the manufacturer's instructions. Only subreads ≥ 5000bp were included for genome assembly.

For *L. sinensis*, genomic DNA was extracted using the E.Z.N.A Mollusc DNA Kit for the next generation short reads sequencing. A pair-end sequencing library with an insertion length

of 350bp was constructed and sequenced on the DNBSEQ-T7 system. Genomic DNA from the same individual was extracted using a Genomic-tip 100G (QIANGEN) kit and sheared and size-selected with the aforementioned procedure for the SMRT library. ONT libraries were constructed with these selected fragments using the Ligation Sequencing 1D Kit (Oxford Nanopore, Oxford, UK, p/n SQK-LSK109) according to the manufacturer's instructions. Sequencing was performed on the PromethION (ONT) platform.

Genomic DNA was extracted from foot muscle tissues of another individual for both species using E.Z.N.A Mollusc DNA Kit. Hi-C fragment libraries were constructed with insert size ranging from 300bp to 700bp and sequenced on HiSeq X Ten and DNBSEQ-T7 system for *L. brevicula* and *L. sinensis* respectively. The Hi-C reads accounts for ~138X coverage of the *L. brevicula* genome and ~123X coverage for the *L. sinensis* genome. Quality control was performed by HiC-Pro v2.8.1 [58]. All the sequencing was performed in the Biomarker Technologies Corporation.

Genome assembly and scaffolding

To assemble the genome of *L. brevicula*, the subreads of *L. brevicula* from PacBio sequencing were assembled using Wtdbg2 v2.5 [59] with parameters: "-x sq -g 1g -X 100 -AS2 --node-len 2048 --aln-dovetail 20480". The resulting contigs were polished by GCpp v2.0.2 (https://github.com/PacificBiosciences/gcpp) using PacBio data first. Hi-C data were used to anchor contigs onto chromosomes using Juicer v1.6 [60] and 3d-DNA [61]. The chromosomal level genome assembly was further adjusted using Juicebox v1.11.08 [62] and gap-filled with TGS-GapCloser v1.2.0 [63] and then polished again with GCpp v2.02.

The ONT long reads of *L. sinensis* were assembled using NextDenovo v2.4.0 (https://github.com/Nextomics/NextDenovo) with parameters: “read_cutoff = 1k, genome_size = 1g”. The assembly was first polished using PEPPER v0.1 (https://github.com/kishwarshafin/pepper/tree/r0.1/models) with ONT long reads, and then DNBSEQ-T7 short reads were aligned to the contigs and single base errors were corrected by FREEBAYES v1.3.4 (https://github.com/freebayes/freebayes/releases/tag/v1.3.4) and PILON v1.2.3 [64]. The genome contigs were scaffolded into chromosomes with Hi-C reads using ALLHiC v0.9.8 [65]. The chromosomal level genome assembly was further adjusted using Juicebox v1.11.08 [62] and gap-filled with TGS-GapCloser v1.2.0 [63] and then polished again with DNBSEQ-T7 reads using FREEBAYES v1.3.4 (https://github.com/freebayes/freebayes/releases/tag/v1.3.4) and PILON v1.2.3 [64].

To assess the genome quality, the completeness of these two genome assemblies were estimated. The completeness of the two genomes was assessed by BUSCO v5.2.1 [66] using the metazoan (metazoa_odb10) database which contains 954 highly conserved single-copy core genes.

Genome annotation

The repeat library was constructed by RepeatModeler v2.0.1 [67] and EDTA v2.0.1 [68] while RepeatMasker v4.1.2 [69] was used to identify and mask repetitive elements of the two littorinid genomes. Based on the repeat-masked genomes, protein-coding genes were predicted using a combination of three approaches: transcriptome-based, de novo, and homologue-based

methods. Firstly, transcripts of the two snails were assembled for transcriptome-based annotation. Illumina short reads of two littorinid snails and full-length PacBio Iso-Seq reads for *L. brevicula* were assembled using Trinity v2.11.0 [70] and ISOSEQ v3 (https://github.com/PacificBiosciences/IsoSeq) respectively and then mapped to the reference genome using MINIMAP2 v2.17 [71]. PASAPIPELINE v2.4.1 [72], STRINGTIE v2.2.1 [73], and TRANSDECODER v5.5.0 (http://transdecoder.sourceforge.net) were used to predict candidate protein-coding regions in two littorinid genomes. Secondly, de novo gene prediction was performed using AUGUSTUS 3.4.0 [74], BRAKER v2.1.6 [75], and GENEMARK v4.69 [76] for both snails. Thirdly, METAEUK [77] was used for homologous gene annotation of the two snails with protein sequences of the following 8 species: *Lottia gigantea*, *Haliotis discus hannai*, *Elysia chlorotica*, *Biomphalaria glabrata*, *Aplysia californica*, *Pomacea canaliculate*, *Octopus bimaculoides*, and *Octopus minor* (Table S1). Finally, the results from the three approaches were integrated using EVidenceModeler v2.0.0 [78] and Funannotate v1.8.15 (https://github.com/nextgenusfs/funannotate). For the prediction of gene function, the predicted protein-coding genes were aligned to the databases of UniProt [79], Pfam-A [80], EggNOG [81], MEROPS [82], CAZYme [83], BUSCO [66], and InterProScan [84].

Gene family, phylogenetic analysis, and divergence time estimation

Protein-coding sequences of *Argopecten purpuratus*, *Biomphalaria glabrata*, *Chlamys farreri*, *Chrysomallon squamiferum*, *Haliotis laevigata*, *Haliotis rubra*, *Nautilus pompilius*, *Patinopecten yessoensis*, *Littorina brevicula*, *Littoraria sinensis* and *Capitella teleta* (outgroup) (Table S1) were aligned using DIAMOND v2.0.14.152 [85] with a cutoff e-value of 1e-5 and

compared using OrthoFinder v2.5.5 [86] to construct gene families.

To infer the phylogenetic relationships, 829 single-copy gene families from all 11 species were extracted to perform multiple alignments using MAFFT v7.429 [87] with default parameter settings. After transformed back to coding DNA and refined by using Gblocks v0.91b [88], all of the alignments were combined into a supergene. The phylogenetic tree was constructed based on the maximum likelihood method in IQ-TREE v1.6.12 [89] with the GTR+F+I+G4 model. Clade support was assessed using bootstrapping algorithm with 1,000 replicates. The divergence time between each clade was estimated with MCMCTree in PAML v4.9 [90]. Three time calibrations retrieved from the Timetree database (http://www.timet ree.org/) and other whole-genome phylogenetic analyses [91, 92] were used for correction.

Expansion and contraction of gene families

The CAFE v5 tool [93] was used to examine gene family expansion and contraction with parameter "-p -k 1". Based on a stochastic birth and death model with the lambda option [94], the size difference of each gene family was checked along each lineage on the phylogenetic tree of 11 species. A probabilistic graphic model was applied to calculate the probability of transitions in gene family size from parent to child nodes. The corresponding *p*-values were calculated for each lineage based on conditional likelihood. Gene families with a p-value ≤ 0.05 were considered to be significantly expanded/contracted and were further subjected to GO functional enrichment analyses using the topGO R package [95].

Identification of positively selected genes related to intertidal adaptation of littorinid snails

Four submerged molluscan species (*A. purpuratus*, *C.farreri*, *P. yessoensis,* and *H. laevigate*) were used as background branches to identify genes under positive selection in the common ancestor of two littorinid snails (foreground branch). These four species inhabit relatively stable sea bottoms and are vulnerable to environmental fluctuation, which is the opposite of littorinid snails. Single-copy orthologous gene families were extracted and an unrooted phylogenetic tree was constructed using the methods mentioned above, based on which CODMEL of PAML package v4.9 [90] was used to identify genes under positive selection in the foreground branch using the branch-site model. Genes were identified as positively selected according to the chi-squared test (p < 0.01, df = 1) and containing amino acid sites that were selected with a BEB higher than 99%.

Macrosynteny analyses

Chromosome-scale collinearity analyses were performed pairwisely for *L. brevicula*, *L. sinensis,* and *P. yessoensis*. Protein sequences were aligned to each other using DIAMOND v2.0.14.152 with parameter “-k1” and single-copy gene families were identified as aforementioned. The macrosynteny analyses were conducted using the MCScanX [96] package with defaulting parameters. The collinearity was then visualized into dot plot figures using the VGSC Java package (https://dvb.ac.cn/vgsc2/service/home.php).

Results

Genome assembly and annotation of two littorinid snails

The PacBio Sequel Ⅱ System and PromethION platform generated a total of 197.66Gb (~212-fold coverage) and 16.58Gb (~20-fold coverage) clean data for *L. brevicula* and *L. sinensis*. To further construct chromosome-level genome assemblies, 129.31Gb and 114.67Gb clean Hi-C reads were obtained for *L. brevicula* and *L. sinensis*, with 92.21% and 99.98% assembled sequences of *L. brevicula* and *L. sinensis* were anchored onto 17 pseudochromosomes (Table S2), which is consistent with previous karyotype analysis [21]. Finally, two chromosome-level genome assemblies spanning 927.94Mb for *L. brevicula* and 822.51Mb for *L. sinensis* were generated, with contig N50 of 3.43Mb and 2.31Mb respectively (Table 1). The completeness of two genome assemblies was assessed using BUSCO [66] and the results demonstrated that 888 (93.1%) and 894 (93.7%) out of 954 metazoan single-copy core genes were present in the genome of *L. brevicula* and *L. sinensis* (Table S3).

Repetitive elements composed 47.25% (438.52Mb) and 41.09% (337.97Mb) of the genome of *L. brevicula* and *L. sinensis* respectively (Table 2). A total of 29,335 and 25,386 genes were predicted for *L. brevicula* and *L. sinensis*. The gene number, gene length, coding sequence (CDS) number, as well as lengths of CDS, intron, and exon were described in Table 3&4. A total of 25,495 (86.91 %) and 23,238 (91.54%) genes were functionally annotated based on different gene databases for *L. brevicula* and *L. sinensis*.

Gene family, phylogenetic, and divergence analyses

According to the gene family clustering analysis, a total of 267,386 (89.99%) genes were assigned to 29,488 orthologous groups, of which 5,017 were shared among all 11 species and 2,950 were specific to the two littorinid snails (Table S5). Functional annotation and GO enrichment were performed, which showed that these littorinid-specific gene families were involved in 142 GO terms relevant to metabolic processes, antioxidant responses, and innate immunity, etc (Table S6).

Based on 829 single-copy gene families, we constructed the species tree for 10 mollusks using *Capitella teleta* as the outgroup (Figure 1). Using genome wide data, the divergence time between *L. brevicula* and *L. sinensis* was estimated to be ~128.2 million years, suggesting a deep divergence between the two littorinid snails, yet they shared highly conserved macrosynteny (see below).

Genetic mechanisms of adaptation to the intertidal environment for littorinids

A total of 92 significantly expanded gene families (involving 897 genes) and 15 contracted gene families (involving 11 genes) were identified for the common littorinid ancestor of *L. brevicula* and *L. sinensis*. The significantly expanded gene families were involved in innate immunity, metabolic processes, stimulus responses, antioxidant responses, etc. (Table S7). Gene families encoding multiple pattern recognition receptors (PRRs) expanded the most among all the 92 littorinid expanded gene families, together with gene families encoding

cytochrome P450 and defense gene sets like HEPN domain-containing proteins might facilitate littorinids to respond to biotic and abiotic stresses and adapt to the intertidal environment.

A total of 720 positively selected genes were identified (p-value ≤ 0.01) for the common ancestor of *L. brevicula* and *L. sinensis*. The functions of these genes were annotated using databases mentioned in section 2.3 and were further confirmed using GeneCards database [97]. Based on the functional annotation of the positively selected genes, we identified 156 genes (Table S8) as strong candidate genes for intertidal adaptation, which were involved in metabolic processes, innate immunity, signal transduction, nucleotide and protein binding, cell cycle regulation and apoptosis processes. Almost half of these candidate genes were related to nucleotide and protein binding processes and involved in damaged DNA/RNA/protein repairment or degradation like HSPA12A.

Evolution of littorinid chromosomes from the ancient bilaterian ancestor

Genome macrosynteny analyses which were independent of intra-chromosomal rearrangements were performed pairwisely among the two littorinid snails and the scallop *P. yessoensis* using orthologous single-copy genes. The results showed extensive collinearity between *L. brevicula* and *L. sinensis* with few inter-chromosomal rearrangements (Figure 2a). Meanwhile, the collinearity between littorinids and the scallop indicated that *P. yessoensis* chromosomes PY8 and PY9 were homologous to littorinid chromosome L1; PY2 and PY19 were homologous to L2; PY11 and PY13 were homologous to L3; PY1 was homologous to L13 and L15, resulting in the difference of chromosome numbers between littorinid snails (n =

17) and *P. yessoensis* (n = 19) (Figure 2b,c). Previous macrosynteny analyses revealed that *P. yessoensis* possessed a highly conserved 19-chromosome karyotype similar to that of bilaterian ancestors [53, 57] and the 19 scallop chromosomes evolved from the 17 presumed ancient linkage groups (ALGs) of bilaterian ancestors through 3 chromosomal fissions (ALG13 to PY5 and PY16; ALG4 to PY9 and PY17; ALG2 to PY13 and PY19) and 1 fusion (ALG5 and ALG16 to PY2). Therefore, the evolutionary trajectory from the 17 ALGs of bilaterian ancestors to the 17 littorinid chromosomes can be inferred based on the macrosynteny analyses: 1) ALG2 fissioned into ALG2-1and ALG2-2; ALG4 fissioned into ALG4-1and ALG4-2; 2) ALG2-1 fused with ALG5 and ALG16 into L2; ALG2-2 fused with ALG11 into L3; ALG4-1 fused with ALG12 into L1; 3) ALG13 fissioned into L5 and L8; ALG10 fissioned into L13 and L15, which indicated that the 17 chromosomes of littorinids evolved from the 17 ALGs of bilaterian ancestors through 4 chromosomal fusions and 4 fissions regardless of intrachromosomal rearrangements (Figure 3).

Discussion

As candidate ecological and evolutionary models, high-quality genomes are urgently needed for littorinid snails but have been hindered by technical obstacles. Here, we generated chromosome-scale genome assemblies for two littorinid marine snails. Assessment and comparation with other published molluscan genomes showed high level of continuity and completeness but moderate level of size and repetitive elements for the two littorinid genomes (Table S4), which ensure the accuracy of comparative genomic analyses in our study and

provide qualified genomic materials for further evolutionary molecular researches in the future.

The adaptation to intertidal environments of littorinids depends on gene families regulating multiple biological processes. Littorinid-specific gene families involved in energy metabolic response (GO:0005975, carbohydrate metabolic process; GO:0004553, hydrolase activity, hydrolyzing O-glycosyl compounds; GO:0016798, hydrolase activity, acting on glycosyl bonds) and antioxidant responses (GO:0006749, glutathione metabolic process; GO:0006979, response to oxidative stress; GO:0004601, peroxidase activity) may facilitate littorinid snails to generate ATP production to compensate for extra energy demands and to eliminate excess reactive oxygen species (ROS) under multiple stresses (hyperthermia, hypoxia, and air exposure, etc) [98-100]. The innate immune system in mollusks is of vital importance in pathogen defense [35, 101, 102]. Gene families encoding multiple pattern recognition receptors (PRRs) expanded the most among all the 92 littorinid expanded gene families, which contained C-type lectin-related proteins (CREPs), fibrinogen-related proteins (FREPs), scavenger receptor cysteine-rich proteins (SRCRs), G-protein coupled receptors (GPCRs), etc. Previous studies suggested that these PRRs were involved in adaptations of mollusks to biotic stresses with diverse functions such as immune recognition, signal transduction, and pathogen elimination through phagocytosis [102-106]. In addition, several transcriptomic and GWAS analyses also indicated that these innate immune genes were involved in response to different types of abiotic stresses like high temperature and desiccation [104, 107]. Energy metabolism is the key factor in establishing limits of environmental stress tolerance [108]. The expanded carbohydrate hydrolase and triglyceride-related gene families (GO:0004806, triglyceride lipase activity; GO:0016810, hydrolase activity, acting on carbon-nitrogen (but not peptide) bonds)

may play a key role in metabolic adaptation to intertidal environments with multiple biotic and abiotic stresses. Regarded as indicator species for measuring the impacts of pollution [27], littorinid snails can withstand high levels of pollutants such as heavy metals. Gene families encoding cytochrome P450 (CYP450) and glutathione S-transferases (GSTs) were expanded, which were involved in iron ion binding processes (GO:0005506) and glutathione metabolic process (GO:0006749). These processes have a known function in constituting the xenobiotic detoxification system of mollusks by detecting and binding with organic and inorganic toxicants like pesticide, sewage, and hydrocarbon contamination [109, 110]. In addition, most biotic and abiotic intertidal stressors like hyperthermia, hypoxia, heavy metals, and pathogens may result in excess reactive oxygen species (ROS) in cells and apoptosis [107, 111]. The expanded defense gene sets including genes encoding HEPN domain-containing proteins and Sacsin, which contained Hsp90-like domains and recruited Hsp70 [112, 113], and might play key roles in maintaining protein homeostasis and anti-apoptosis processes in littorinid snails [114].

Compared to the littorinid-specific and expanded gene families, functions of the positively selected genes were more unified. Almost half of the candidate intertidal adaptation positively selected genes like HSPA12A, Lon protease homolog (LONP1), Zinc finger HIT domain-containing protein 2 (ZNHIT2), Dus domain-containing protein (DUS1L) are involved in nucleotide and protein binding processes and have known functions in repairing or degrading damaged DNA/RNA/protein caused by various biotic and abiotic stresses [115-117]. There were also several genes (DNAJB12, AHSA1, etc.) that indirectly facilitate the maintenance of protein homeostasis in severe environments by activating heat shock proteins [118, 119]. In addition, the positively selected hypoxia-inducible factor 1-alpha (HIF1A) might be a key gene

for littorinid snails in adaptation to anoxic conditions caused by high intertidal temperature and air exposure, which may induce tissue hypoxemia and extra ROS and make intertidal organisms switch to anaerobic metabolism [35]. HIF1A was found to be highly expressed under air exposure in oysters [120] and the HIF pathway motivates this transition by regulating glucose catabolism, oxidative phosphorylation, and antioxidative processes, leading to decreased ATP production in mammals [121]. The results suggesting that the maintenance of cellular homeostasis and repairing of damaged nucleotides and proteins were main strategies for littorinid snails to hinder the cell apoptosis processes caused by environmental stresses and facilitate adaptation to the harsh intertidal environment.

Although the 17 littorinid chromosomes did not possess a complete ‘1 to 1’ conserved model with the 17 presumed bilaterian ALGs, our analyses revealed that most littorinid chromosomes (9) directly inherited ancient bilaterian gene linkages while the other 8 chromosomes evolved from 4 chromosomal fissions and 4 fusions (Figure 3). Surprisingly, all of the 3 chromosomal fissions and 1 chromosomal fusion between the bilaterian ancestors and *P. yessoensis* were also found between littorinid snails and the bilaterian ancestors, which implied that they occurred before the bivalve-gastropod split (Figure 3) around 500 million years ago. Overall, the level of chromosome preservation was comparable for the scallop lineage and the littorinid lineage. Considering the sister relationship between Bivalvia and Gastropoda, these results demonstrated that the chromosome-scale ancient gene linkages were generally preserved in the mollusk genomes over 500 million years, which added evidence to the conclusion that slow chromosome evolution was widespread among invertebrates [52]. In addition, *L. brevicula* and *L. sinensis* showed nearly perfect collinearity despite that these two

species diverged over 100 million years ago, also implying the ‘slow-evolving’ feature of littorinid snails. Wang et al. [57] proposed that the remarkable conservation of ancestral features in scallop genome is probably as a consequence of life on cold and stable deep-ocean bottoms. However, although the littorinid snails live in the harsh and highly fluctuating intertidal environments, they still have high level of chromosome preservation with the bilaterian ancestors, which is similar to that of scallop. The results implied that living environments might not be the key driver of karyotype evolution in mollusks, other evolutionary or developmental constraints on the evolution of genome organization could exist.

Conclusions

Understanding the evolutionary adaptation to harsh fluctuating environments and early evolution of bilaterians is of great importance in evolutionary biology, and the advances of comparative genomic analyses accompanying the development of genome sequencing technology facilitate these studies. The intertidal gastropoda mollusks, littorinid snails, provide excellent models for addressing these questions. By overcoming technical challenges, we generated high-quality, chromosome-scale genome assemblies for two wide-spread littorinid marine snails in the northeast Pacific. Comparative genomic analyses revealed clear adaptive genomic signals for evolutionary adaptation to the harsh fluctuating intertidal environments. Gene families associated with stimulus responses, metabolic processes, innate immunity, and antioxidant response were identified to be specific to or expanded in the littorinid lineage, suggesting that these genes might be related to cellular homeostasis system, pathogen

elimination, and anti-apoptosis processes and finally facilitated the adaptation to harsh intertidal environments with multiple biotic and abiotic stresses. Genes relevant to damaged protein/DNA/RNA repair and elimination were positively selected and might play key roles in cellular homeostasis maintenance in stressful environments. Genome macrosynteny analyses indicated that the 17 littorinid chromosomes evolved from the 17 bilaterian ALGs through 4 fissions and 4 fusions, showing their high level of karyotypic conservation with the bilaterian ancestors. Three chromosomal fissions and 1 chromosomal fusion of the bilaterian ALGs were shared by the scallop and littorinid snails, indicating that the chromosome-scale ancient gene linkages were generally preserved in the mollusk genomes over 500 million years. Similar studies, particularly of chromosome level genomes from other basal bilaterians, may lead to the eventual reconstruction of urbilaterian chromosomes, which can greatly improve our understanding of bilaterian evolution.

Acknowledgements

This work was supported by the National Natural Science Foundation of China (Grant Nos. 319700488, 31972793).

Additional Files

Supplementary Table S1. Metazoan genome assemblies and gene models used in this study.

Supplementary Table S2. Statistics of chromosomal level assembly of the two littorinid snails.

Supplementary Table S3. Completeness assessment of the two littorinid snails by BUSCO.

Supplementary Table S4. Genome size and repetitive elements of 46 mollusks.

Supplementary Table S5. Statistics of gene families of 11 species in comparative genomic analysis.

Supplementary Table S6. Gene ontology of the littorinid-specific gene families.

Supplementary Table S7. Gene ontology of the expanded gene families of littorinid ancestor.

Supplementary Table S8. Intertidal adaptation-related genes under positive selection.

Abbreviations

ALG: ancient linkage group; BEB: Bayesian and empirical Bayes approach; bp: base pairs; BUSCO: Benchmarking Universal Single-Copy Orthologs; CTAB: cetyl trimethyl ammonium bromide; Evo-Devo: evolutionary developmental biology; Gb: gigabase pairs; GO: Gene Ontology; HEPN: higher eukaryotic and prokaryotic nucleasedomains; HSP: heat shock protein; HiC: high-throughput/resolution chromosome conformation capture; kb: kilobase pairs; Mb: megabase pairs; Mya: million years ago; NCBI: National Center for Biotechnology Information; ONT: Oxford Nanopore Technologies; Pacbio: Pacific Biosciences.

Author Contributions

J.-X. L. conceived and supervised the study; Y.-S. W., M. -Y. L. performed the research; Y.-S. W., M. -Y. L. and Y.-L. L. analyzed the data; Y.-S. W. and J.-X. L. wrote the manuscript. All authors discussed the results and commented on the manuscript.

Competing Interests

The authors declare that they have no competing interests.

Data Availability

The sequencing data that support the findings of this study are openly available in the NCBI Sequence Read Archive (SRA) under BioProject accession number PRJNA1032305 (*Littorina brevicula*) and PRJNA1032307 (*Littoraria sinensis*). The genome assembly and annotation data of *Littorina brevicula* (https://figshare.com/s/74caa5554fc13b9910bb) and *Littoraria sinensis* (https://figshare.com/s/c0d47d3fb2ed21567698) have been deposited in FigShare.

References

1. Chemello S, Vizzini S and Mazzola A. Regime shifts and alternative stable states in intertidal rocky habitats: State of the art and new trends of research. Estuarine, Coastal and Shelf Science. 2018;214:57-63. doi:10.1016/j.ecss.2018.09.013.
2. Bernhardt JR, O'Connor MI, Sunday JM and Gonzalez A. Life in fluctuating environments. Philos Trans R Soc Lond B Biol Sci. 2020;375 1814:20190454. doi:10.1098/rstb.2019.0454.
3. Vasseur DA and McCann KS. The Impact of Environmental Variability on Ecological Systems. Springer, Dordrecht; 2007.
4. Blewett TA, Binning SA, Weinrauch AM, Ivy CM, Rossi GS, Borowiec BG, et al.

Physiological and behavioural strategies of aquatic animals living in fluctuating environments. J Exp Biol. 2022;225 9 doi:10.1242/jeb.242503.

5. Thompson RC, Crowe TP and Hawkins SJ. Rocky intertidal communities: past environmental changes, present status and predictions for the next 25 years. Environmental Conservation. 2002;29 2:168-91. doi:10.1017/s0376892902000115.

6. Raffaelli D and Hawkins S. Intertidal Ecology. 1 ed.: Springer, Dordrecht; 1996.

7. Helmuth B, Mieszkowska N, Moore P and Hawkins SJ. Living on the Edge of Two Changing Worlds: Forecasting the Responses of Rocky Intertidal Ecosystems to Climate Change. Annual Review of Ecology, Evolution, and Systematics. 2006;37 1:373-404. doi:10.1146/annurev.ecolsys.37.091305.110149.

8. Bertness MD, Leonard GH, Levine JM and Bruno JF. Climate-driven interactions among rocky intertidal organisms caught between a rock and a hot place. Oecologia. 1999;120 3:446-50. doi:10.1007/s004420050877.

9. Connell JH. Community Interactions on Marine Rocky Intertidal Shores. 1972;3 1:169-92. doi:10.1146/annurev.es.03.110172.001125.

10. Somero GN. Thermal Physiology and Vertical Zonation of Intertidal Animals: Optima, Limits, and Costs of Living1. Integrative and Comparative Biology. 2002;42 4:780-9. doi:10.1093/icb/42.4.780 %J Integrative and Comparative Biology.

11. Storey KB, Lant B, Anozie OO and Storey JM. Metabolic mechanisms for anoxia tolerance and freezing survival in the intertidal gastropod, Littorina littorea. Comp Biochem Physiol A Mol Integr Physiol. 2013;165 4:448-59. doi:10.1016/j.cbpa.2013.03.009.

12. Sokolova IM and Portner HO. Physiological adaptations to high intertidal life involve improved water conservation abilities and metabolic rate depression in Littorina saxatilis. Mar Ecol Prog Ser. 2001;224:171-86. doi:DOI 10.3354/meps224171.

13. Appeltans W, Ahyong ST, Anderson G, Angel MV, Artois T, Bailly N, et al. The magnitude of global marine species diversity. Curr Biol. 2012;22 23:2189-202. doi:10.1016/j.cub.2012.09.036.

14. Oliveira ES, Torres DF, Brooks SE and Alves RR. The medicinal animal markets in the metropolitan region of Natal City, northeastern Brazil. Journal of ethnopharmacology. 2010;130 1:54-60. doi:10.1016/j.jep.2010.04.010.

15. Liu C, Ren Y, Li Z, Hu Q, Yin L, Wang H, et al. Giant African snail genomes provide insights into molluscan whole-genome duplication and aquatic-terrestrial transition. Mol Ecol Resour. 2021;21 2:478-94. doi:10.1111/1755-0998.13261.

16. Kohn AJ. EVOLUTION AND ESCALATION - AN ECOLOGICAL HISTORY OF LIFE - VERMEIJ,GJ. Science. 1987;237 4819:1235-6. doi:10.1126/science.237.4819.1235.

17. Cortez T, Amaral RV, Sobral-Souza T and Andrade SCS. Genome-wide assessment elucidates connectivity and the evolutionary history of the highly dispersive marine invertebrate Littoraria flava (Littorinidae: Gastropoda). Biological Journal of the Linnean Society. 2021;133 4:999-1015. doi:10.1093/biolinnean/blab055.

18. Chueca LJ, Schell T and Pfenninger M. De novo genome assembly of the land snail Candidula unifasciata (Mollusca: Gastropoda). G3 (Bethesda). 2021;11 8 doi:10.1093/g3journal/jkab180.

19. Solem A and Bruggen ACv. World-wide snails : biogeographical studies on non-marine Mollusca. In: 1984.

20. Williams ST, Reid DG and Littlewood DT. A molecular phylogeny of the Littorininae (Gastropoda: Littorinidae): unequal evolutionary rates, morphological parallelism, and biogeography of the Southern Ocean. Mol Phylogenet Evol. 2003;28 1:60-86. doi:10.1016/s1055-7903(03)00038-1.

21. Garcia-Souto D, Alonso-Rubido S, Costa D, Eirin-Lopez JM, Rolan-Alvarez E, Faria R, et al. Karyotype Characterization of Nine Periwinkle Species (Gastropoda, Littorinidae). Genes (Basel). 2018;9 11 doi:10.3390/genes9110517.

22. Rolán-Alvarez E, Austin C and Boulding E. The Contribution of the Genus Littorina to the Field of Evolutionary Ecology. Oceanography and marine biology. 2015;53:157-214. doi:10.1201/b18733-6.

23. Ng TPT, Lau SLY, Seuront L, Davies MS, Stafford R, Marshall DJ, et al. Linking behaviour and climate change in intertidal ectotherms: insights from littorinid snails. Journal of Experimental Marine Biology and Ecology. 2017;492:121-31. doi:10.1016/j.jembe.2017.01.023.

24. Johannesson K. What can be learnt from a snail? Evolutionary Applications. 2016;9 1:153-65. doi:10.1111/eva.12277.

25. Johannesson K, Panova M, Kemppainen P, Andre C, Rolan-Alvarez E and Butlin RK. Repeated evolution of reproductive isolation in a marine snail: unveiling mechanisms of speciation. Philosophical Transactions of the Royal Society B-Biological Sciences. 2010;365 1547:1735-47. doi:10.1098/rstb.2009.0256.

26. Johannesson K. Evolution in Littorina: ecology matters. Journal of Sea Research. 2003;49 2:107-17. doi:10.1016/s1385-1101(02)00218-6.

27. Ravinet M. Notes from a snail island: Littorinid evolution and adaptation. Mol Ecol. 2018;27 13:2781-9. doi:10.1111/mec.14730.

28. Li YQ, Li MY, Xing TF and Liu JX. Resolving the origins of invertebrate colonists in the Yangtze River Estuary with molecular markers: Implications for ecological connectivity. Ecol Evol. 2021;11 20:13898-911. doi:10.1002/ece3.8095.

29. Li M, Li Y, Xing T, Li Y and Liu J. Microsatellite marker development and population genetic analysis revealed high connectivity between populations of a periwinkle Littoraria sinensis (Philippi, 1847). Journal of Oceanology and Limnology. 2022;40 3:1097-109. doi:10.1007/s00343-021-1079-9.

30. Okutani T. Marine mollusks in Japan. In: 2000.

31. Reid DG. Systematics and evolution of Littorina. London :: Ray Society, 1996.

32. Chiba S, Iida T, Tomioka A, Azuma N, Kurihara T and Tanaka K. Population divergence in cold tolerance of the intertidal gastropod Littorina brevicula explained by habitat-specific lowest air temperature. Journal of Experimental Marine Biology and Ecology. 2016;481:49-56. doi:10.1016/j.jembe.2016.04.009.

33. Dong YW, Liao ML, Han GD and Somero GN. An integrated, multi-level analysis of thermal effects on intertidal molluscs for understanding species distribution patterns. Biol Rev Camb Philos Soc. 2022;97 2:554-81. doi:10.1111/brv.12811.

34. Stankowski S, Zagrodzka ZB, Garlovsky MD, Pal A, Shipilina D, Castillo DG, et al. The genetic basis of a recent transition to live-bearing in marine snails. 2024;383

6678:114-9. doi:doi:10.1126/science.adi2982.

35. Zhang G, Li L, Meng J, Qi H, Qu T, Xu F, et al. Molecular Basis for Adaptation of Oysters to Stressful Marine Intertidal Environments. Annu Rev Anim Biosci. 2016;4:357-81. doi:10.1146/annurev-animal-022114-110903.

36. Amarasinghe SL, Su S, Dong XY, Zappia L, Ritchie ME and Gouil Q. Opportunities and challenges in long-read sequencing data analysis. Genome Biology. 2020;21 1 doi:10.1186/s13059-020-1935-5.

37. Lang DD, Zhang SL, Ren PP, Liang F, Sun ZY, Meng GL, et al. Comparison of the two up-to-date sequencing technologies for genome assembly: HiFi reads of Pacific Biosciences Sequel II system and ultralong reads of Oxford Nanopore. Gigascience. 2020;9 12 doi:10.1093/gigascience/giaa123.

38. Gomes-dos-Santos A, Lopes-Lima M, Castro LFC and Froufe E. Molluscan genomics: the road so far and the way forward. Hydrobiologia. 2019;847 7:1705-26. doi:10.1007/s10750-019-04111-1.

39. Schultzhaus JN, Taitt CR, Orihuela B, Smerchansky M, Schultzhaus ZS, Rittschof D, et al. Comparison of seven methods for DNA extraction from prosomata of the acorn barnacle, Amphibalanus amphitrite. Analytical Biochemistry. 2019;586 doi:10.1016/j.ab.2019.113441.

40. Mayjonade B, Gouzy J, Donnadieu C, Pouilly N, Marande W, Callot C, et al. Extraction of high-molecular-weight genomic DNA for long-read sequencing of single molecules. Biotechniques. 2017;62 1 doi:10.2144/000114503.

41. Panova M, Aronsson H, Cameron RA, Dahl P, Godhe A, Lind U, et al. DNA Extraction

Protocols for Whole-Genome Sequencing in Marine Organisms. Marine Genomics. 2016. p. 13-44.

42. Winnepenninckx B, Backeljau T and De Wachter R. Extraction of high molecular weight DNA from molluscs. Trends in genetics : TIG. 1993;9 12:407. doi:10.1016/0168-9525(93)90102-n.

43. Westram AM, Rafajlović M, Chaube P, Faria R, Larsson T, Panova M, et al. Clines on the seashore: The genomic architecture underlying rapid divergence in the face of gene flow. Evolution Letters. 2018;2 4:297-309. doi:10.1002/evl3.74.

44. Boardman RS, Cheetham, A. H., and Rowell, A. J. Fossil Invertebrates. Boston: Blackwell Scientific Publications; 1987.

45. Fedonkin M and Waggoner B. Fedonkin, M. A. & Waggoner, B. M. The Late Precambrian fossil Kimberella is a mollusc-like bilaterian organism. Nature 388, 868-871. Nature. 1997;388:868-71. doi:10.1038/42242.

46. Simakov O, Marletaz F, Cho SJ, Edsinger-Gonzales E, Havlak P, Hellsten U, et al. Insights into bilaterian evolution from three spiralian genomes. Nature. 2013;493 7433:526-31. doi:10.1038/nature11696.

47. Guerra M. Chromosome numbers in plant cytotaxonomy: concepts and implications. Cytogenetic and genome research. 2008;120 3-4:339-50. doi:10.1159/000121083.

48. Robinson TJ. M. King., Species Evolution: The Role of Chromosome Change. Systematic Biology. 1995;44 4:578-80. doi:10.1093/sysbio/44.4.578 %J Systematic Biology.

49. Auvinet J, Graça P, Dettai A, Amores A, Postlethwait JH, Detrich HW, et al. Multiple

independent chromosomal fusions accompanied the radiation of the Antarctic teleost genus Trematomus (Notothenioidei:Nototheniidae). BMC Evolutionary Biology. 2020;20 1:39. doi:10.1186/s12862-020-1600-3.

50. Baltisberger M and Hörandl E. Karyotype evolution supports the molecular phylogeny in the genus Ranunculus (Ranunculaceae). Perspectives in Plant Ecology, Evolution and Systematics. 2016;18:1-14. doi:https://doi.org/10.1016/j.ppees.2015.11.001.

51. Buerkle A, Yoshida K and Kitano J. Tempo and mode in karyotype evolution revealed by a probabilistic model incorporating both chromosome number and morphology. PLOS Genetics. 2021;17 4 doi:10.1371/journal.pgen.1009502.

52. Simakov O, Bredeson J, Berkoff K, Marletaz F, Mitros T, Schultz DT, et al. Deeply conserved synteny and the evolution of metazoan chromosomes. Science Advances. 2022;8 5 doi:ARTN eabi5884

10.1126/sciadv.abi5884.

53. Simakov O, Marletaz F, Yue JX, O'Connell B, Jenkins J, Brandt A, et al. Deeply conserved synteny resolves early events in vertebrate evolution. Nat Ecol Evol. 2020;4 6:820-30. doi:10.1038/s41559-020-1156-z.

54. JANSON K. CHROMOSOME NUMBER IN TWO PHENOTYPICALLY DISTINCT POPULATIONS OF LITTORINA SAXATILIS OLIVI, AND IN SPECIMENS OF THE LITTORINA OBTUSATA (L.) SPECIES-COMPLEX. Journal of Molluscan Studies. 1983;49 3:224-7. doi:10.1093/oxfordjournals.mollus.a065716 %J Journal of Molluscan Studies.

55. Libertini A, Trisolini R and Edmands S. A cytogenetic study of the periwinkle Littorina

keenae Rosewater, 1978 (Gastropoda: Littorinidae). Journal of Molluscan Studies. 2004;70 3:299-301. doi:10.1093/mollus/70.3.299 %J Journal of Molluscan Studies.

56. Vitturi R, Libertini A, Panozzo M and Mezzapelle G. KARYOTYPE ANALYSIS AND GENOME SIZE IN 3 MEDITERRANEAN SPECIES OF PERIWINKLES (PROSOBRANCHIA, MESOGASTROPODA). Malacologia. 1995;37 1:123-32.

57. Wang S, Zhang J, Jiao W, Li J, Xun X, Sun Y, et al. Scallop genome provides insights into evolution of bilaterian karyotype and development. Nat Ecol Evol. 2017;1 5:120. doi:10.1038/s41559-017-0120.

58. Servant N, Varoquaux N, Lajoie BR, Viara E, Chen CJ, Vert JP, et al. HiC-Pro: an optimized and flexible pipeline for Hi-C data processing. Genome Biology. 2015;16 doi:10.1186/s13059-015-0831-x.

59. Ruan J and Li H. Fast and accurate long-read assembly with wtdbg2. Nature Methods. 2020;17 2:155-+. doi:10.1038/s41592-019-0669-3.

60. Durand NC, Shamim MS, Machol I, Rao SSP, Huntley MH, Lander ES, et al. Juicer Provides a One-Click System for Analyzing Loop-Resolution Hi-C Experiments. Cell Systems. 2016;3 1:95-8. doi:10.1016/j.cels.2016.07.002.

61. Dudchenko O, Batra SS, Omer AD, Nyquist SK, Hoeger M, Durand NC, et al. De novo assembly of the Aedes aegypti genome using Hi-C yields chromosome-length scaffolds. Science. 2017;356 6333:92-5. doi:10.1126/science.aal3327.

62. Robinson JT, Turner D, Durand NC, Thorvaldsdottir H, Mesirov JP and Aiden EL. Juicebox.js Provides a Cloud-Based Visualization System for Hi-C Data. Cell Systems. 2018;6 2:256-+. doi:10.1016/j.cels.2018.01.001.

63. Xu MY, Guo LD, Gu SQ, Wang O, Zhang R, Peters BA, et al. TGS-GapCloser: A fast and accurate gap closer for large genomes with low coverage of error-prone long reads. Gigascience. 2020;9 9 doi:10.1093/gigascience/giaa094.

64. Walker BJ, Abeel T, Shea T, Priest M, Abouelliel A, Sakthikumar S, et al. Pilon: An Integrated Tool for Comprehensive Microbial Variant Detection and Genome Assembly Improvement. Plos One. 2014;9 11 doi:10.1371/journal.pone.0112963.

65. Zhang X, Zhang S, Zhao Q, Ming R and Tang H. Assembly of allele-aware, chromosomal-scale autopolyploid genomes based on Hi-C data. Nature Plants. 2019;5 8:833-45. doi:10.1038/s41477-019-0487-8.

66. Waterhouse RM, Seppey M, Simão FA, Manni M, Ioannidis P, Klioutchnikov G, et al. BUSCO Applications from Quality Assessments to Gene Prediction and Phylogenomics. Molecular Biology and Evolution. 2017;35 3:543-8. doi:10.1093/molbev/msx319 %J Molecular Biology and Evolution.

67. Price AL, Jones NC and Pevzner PA. De novo identification of repeat families in large genomes. Bioinformatics. 2005;21 suppl_1:i351-i8. doi:10.1093/bioinformatics/bti1018 %J Bioinformatics.

68. Su W, Ou S, Hufford MB and Peterson T. A Tutorial of EDTA: Extensive De Novo TE Annotator. Methods in molecular biology (Clifton, NJ). 2021;2250:55-67. doi:10.1007/978-1-0716-1134-0_4.

69. Chen N. Using RepeatMasker to identify repetitive elements in genomic sequences. Current protocols in bioinformatics. 2004;Chapter 4:Unit 4.10. doi:10.1002/0471250953.bi0410s05.

70. Grabherr MG, Haas BJ, Yassour M, Levin JZ, Thompson DA, Amit I, et al. Full-length transcriptome assembly from RNA-Seq data without a reference genome. Nature Biotechnology. 2011;29 7:644-U130. doi:10.1038/nbt.1883.

71. Li H. Minimap2: pairwise alignment for nucleotide sequences. Bioinformatics. 2018;34 18:3094-100. doi:10.1093/bioinformatics/bty191 %J Bioinformatics.

72. Haas BJ, Salzberg SL, Zhu W, Pertea M, Allen JE, Orvis J, et al. Automated eukaryotic gene structure annotation using EVidenceModeler and the program to assemble spliced alignments. Genome Biology. 2008;9 1 doi:10.1186/gb-2008-9-1-r7.

73. Shumate A, Wong B, Pertea G and Pertea M. Improved transcriptome assembly using a hybrid of long and short reads with StringTie. Plos Computational Biology. 2022;18 6 doi:10.1371/journal.pcbi.1009730.

74. Stanke M, Keller O, Gunduz I, Hayes A, Waack S and Morgenstern B. AUGUSTUS: ab initio prediction of alternative transcripts. Nucleic acids research. 2006;34 suppl_2:W435-W9. doi:10.1093/nar/gkl200 %J Nucleic Acids Research.

75. Hoff KJ, Lomsadze A, Borodovsky M and Stanke M. Whole-Genome Annotation with BRAKER. Methods in molecular biology (Clifton, NJ). 2019;1962:65-95. doi:10.1007/978-1-4939-9173-0_5.

76. Tatusova T, DiCuccio M, Badretdin A, Chetvernin V, Nawrocki EP, Zaslavsky L, et al. NCBI prokaryotic genome annotation pipeline. Nucleic acids research. 2016;44 14:6614-24. doi:10.1093/nar/gkw569.

77. Karin EL, Mirdita M and Soding J. MetaEuk-sensitive, high-throughput gene discovery, and annotation for large-scale eukaryotic metagenomics. Microbiome. 2020;8 1

doi:10.1186/s40168-020-00808-x.

78. Haas BJ, Salzberg SL, Zhu W, Pertea M, Allen JE, Orvis J, et al. Automated eukaryotic gene structure annotation using EVidenceModeler and the Program to Assemble Spliced Alignments. Genome Biology. 2008;9 1:R7. doi:10.1186/gb-2008-9-1-r7.

79. Consortium TU. UniProt: the Universal Protein Knowledgebase in 2023. Nucleic acids research. 2022;51 D1:D523-D31. doi:10.1093/nar/gkac1052 %J Nucleic Acids Research.

80. Mistry J, Chuguransky S, Williams L, Qureshi M, Salazar Gustavo A, Sonnhammer ELL, et al. Pfam: The protein families database in 2021. Nucleic acids research. 2020;49 D1:D412-D9. doi:10.1093/nar/gkaa913 %J Nucleic Acids Research.

81. Huerta-Cepas J, Szklarczyk D, Heller D, Hernández-Plaza A, Forslund SK, Cook H, et al. eggNOG 5.0: a hierarchical, functionally and phylogenetically annotated orthology resource based on 5090 organisms and 2502 viruses. Nucleic acids research. 2018;47 D1:D309-D14. doi:10.1093/nar/gky1085 %J Nucleic Acids Research.

82. Rawlings ND, Barrett AJ, Thomas PD, Huang X, Bateman A and Finn RD. The MEROPS database of proteolytic enzymes, their substrates and inhibitors in 2017 and a comparison with peptidases in the PANTHER database. Nucleic acids research. 2017;46 D1:D624-D32. doi:10.1093/nar/gkx1134 %J Nucleic Acids Research.

83. Drula E, Garron M-L, Dogan S, Lombard V, Henrissat B and Terrapon N. The carbohydrate-active enzyme database: functions and literature. Nucleic acids research. 2021;50 D1:D571-D7. doi:10.1093/nar/gkab1045 %J Nucleic Acids Research.

84. Jones P, Binns D, Chang H-Y, Fraser M, Li W, McAnulla C, et al. InterProScan 5:

genome-scale protein function classification. Bioinformatics. 2014;30 9:1236-40. doi:10.1093/bioinformatics/btu031 %J Bioinformatics.

85. Buchfink B, Reuter K and Drost H-G. Sensitive protein alignments at tree-of-life scale using DIAMOND. Nature Methods. 2021;18 4:366-8. doi:10.1038/s41592-021-01101-x.

86. Emms DM and Kelly S. OrthoFinder: phylogenetic orthology inference for comparative genomics. Genome Biol. 2019;20 1:238. doi:10.1186/s13059-019-1832-y.

87. Katoh K and Standley DM. MAFFT multiple sequence alignment software version 7: improvements in performance and usability. Mol Biol Evol. 2013;30 4:772-80. doi:10.1093/molbev/mst010.

88. Castresana J. Selection of Conserved Blocks from Multiple Alignments for Their Use in Phylogenetic Analysis. Molecular Biology and Evolution. 2000;17 4:540-52. doi:10.1093/oxfordjournals.molbev.a026334 %J Molecular Biology and Evolution.

89. Nguyen L-T, Schmidt HA, von Haeseler A and Minh BQ. IQ-TREE: A Fast and Effective Stochastic Algorithm for Estimating Maximum-Likelihood Phylogenies. Molecular Biology and Evolution. 2014;32 1:268-74. doi:10.1093/molbev/msu300 %J Molecular Biology and Evolution.

90. Yang Z. PAML 4: Phylogenetic Analysis by Maximum Likelihood. Molecular Biology and Evolution. 2007;24 8:1586-91. doi:10.1093/molbev/msm088 %J Molecular Biology and Evolution.

91. Sun J, Mu H, Ip JCH, Li R, Xu T, Accorsi A, et al. Signatures of Divergence, Invasiveness, and Terrestrialization Revealed by Four Apple Snail Genomes. Mol Biol

Evol. 2019;36 7:1507-20. doi:10.1093/molbev/msz084.

92. Huang Z, Huang W, Liu X, Han Z, Liu G, Boamah GA, et al. Genomic insights into the adaptation and evolution of the nautilus, an ancient but evolving "living fossil". Mol Ecol Resour. 2022;22 1:15-27. doi:10.1111/1755-0998.13439.

93. Mendes FK, Vanderpool D, Fulton B and Hahn MW. CAFE 5 models variation in evolutionary rates among gene families. Bioinformatics. 2021;36 22-23:5516-8. doi:10.1093/bioinformatics/btaa1022.

94. Han MV, Thomas GWC, Lugo-Martinez J and Hahn MW. Estimating Gene Gain and Loss Rates in the Presence of Error in Genome Assembly and Annotation Using CAFE 3. Molecular Biology and Evolution. 2013;30 8:1987-97. doi:10.1093/molbev/mst100 %J Molecular Biology and Evolution.

95. Alexa A and Rahnenfuhrer J. topGO: Enrichment Analysis for Gene Ontology. R package version 2.54.0. 2023; doi:doi:10.18129/B9.bioc.topGO.

96. Wang Y, Tang H, Debarry JD, Tan X, Li J, Wang X, et al. MCScanX: a toolkit for detection and evolutionary analysis of gene synteny and collinearity. Nucleic acids research. 2012;40 7:e49. doi:10.1093/nar/gkr1293.

97. Stelzer G, Rosen N, Plaschkes I, Zimmerman S, Twik M, Fishilevich S, et al. The GeneCards Suite: From Gene Data Mining to Disease Genome Sequence Analyses. Curr Protoc Bioinformatics. 2016;54:1.30.1-1..3. doi:10.1002/cpbi.5.

98. Meng J, Zhu Q, Zhang L, Li C, Li L, She Z, et al. Genome and transcriptome analyses provide insight into the euryhaline adaptation mechanism of Crassostrea gigas. PLoS One. 2013;8 3:e58563. doi:10.1371/journal.pone.0058563.

99. Sokolova IM, Frederich M, Bagwe R, Lannig G and Sukhotin AA. Energy homeostasis as an integrative tool for assessing limits of environmental stress tolerance in aquatic invertebrates. Mar Environ Res. 2012;79:1-15. doi:10.1016/j.marenvres.2012.04.003.

100. Ivanina AV, Froelich B, Williams T, Sokolov EP, Oliver JD and Sokolova IM. Interactive effects of cadmium and hypoxia on metabolic responses and bacterial loads of eastern oysters Crassostrea virginica Gmelin. Chemosphere. 2011;82 3:377-89. doi:10.1016/j.chemosphere.2010.09.075.

101. Hoebe K, Janssen E and Beutler B. The interface between innate and adaptive immunity. Nature immunology. 2004;5 10:971-4. doi:10.1038/ni1004-971.

102. Dheilly NM, Duval D, Mouahid G, Emans R, Allienne J-F, Galinier R, et al. A family of variable immunoglobulin and lectin domain containing molecules in the snail Biomphalaria glabrata. Developmental & Comparative Immunology. 2015;48 1:234-43. doi:10.1016/j.dci.2014.10.009.

103. Li L, Li A, Song K, Meng J, Guo X, Li S, et al. Divergence and plasticity shape adaptive potential of the Pacific oyster. Nature Ecology & Evolution. 2018;2 11:1751-60. doi:10.1038/s41559-018-0668-2.

104. Zhang L, Li L, Guo X, Litman GW, Dishaw LJ and Zhang G. Massive expansion and functional divergence of innate immune genes in a protostome. Sci Rep. 2015;5:8693. doi:10.1038/srep08693.

105. Hanington PC and Zhang S-M. The Primary Role of Fibrinogen-Related Proteins in Invertebrates Is Defense, Not Coagulation. Journal of Innate Immunity. 2011;3 1:17-27. doi:10.1159/000321882.

106. Liu L, Yang J, Qiu L, Wang L, Zhang H, Wang M, et al. A novel scavenger receptor-cysteine-rich (SRCR) domain containing scavenger receptor identified from mollusk mediated PAMP recognition and binding. Developmental & Comparative Immunology. 2011;35 2:227-39. doi:10.1016/j.dci.2010.09.010.

107. Zhao Z, Gan H, Lin X, Wang L, Yao Y, Li L, et al. Genome-wide association screening and MassARRAY for detection of high-temperature resistance-related SNPs and genes in a hybrid abalone (Haliotis discus hannai ♀ × H. fulgens ♂) based on super genotyping-by-sequencing. Aquaculture. 2023;573 doi:10.1016/j.aquaculture.2023.739576.

108. Sokolova IM, Frederich M, Bagwe R, Lannig G and Sukhotin AA. Energy homeostasis as an integrative tool for assessing limits of environmental stress tolerance in aquatic invertebrates. Marine Environmental Research. 2012;79:1-15. doi:https://doi.org/10.1016/j.marenvres.2012.04.003.

109. Schlenk D and Buhler DR. Xenobiotic biotransformation in the Pacific oyster (Crassostrea gigas). Comparative biochemistry and physiology C, Comparative pharmacology and toxicology. 1989;94 2:469-75. doi:10.1016/0742-8413(89)90100-x.

110. Rodrigues-Silva C, Flores-Nunes F, Vernal JI, Cargnin-Ferreira E and Bainy AC. Expression and immunohistochemical localization of the cytochrome P450 isoform 356A1 (CYP356A1) in oyster Crassostrea gigas. Aquatic toxicology (Amsterdam, Netherlands). 2015;159:267-75. doi:10.1016/j.aquatox.2014.12.021.

111. Cheng J, Hui M and Sha Z. Transcriptomic analysis reveals insights into deep-sea adaptations of the dominant species, Shinkaia crosnieri (Crustacea: Decapoda:

Anomura), inhabiting both hydrothermal vents and cold seeps. BMC Genomics. 2019;20 1:388. doi:10.1186/s12864-019-5753-7.

112. Anderson JF, Siller E and Barral JM. The sacsin repeating region (SRR): a novel Hsp90-related supra-domain associated with neurodegeneration. Journal of molecular biology. 2010;400 4:665-74. doi:10.1016/j.jmb.2010.05.023.

113. Perna L, Castelli M, Frasnetti E, Romano LEL, Colombo G, Prodromou C, et al. AlphaFold predicted structure of the Hsp90-like domains of the neurodegeneration linked protein sacsin reveals key residues for ATPase activity. Front Mol Biosci. 2022;9:1074714. doi:10.3389/fmolb.2022.1074714.

114. Anantharaman V, Makarova KS, Burroughs AM, Koonin EV and Aravind L. Comprehensive analysis of the HEPN superfamily: identification of novel roles in intra-genomic conflicts, defense, pathogenesis and RNA processing. Biology direct. 2013;8:15. doi:10.1186/1745-6150-8-15.

115. Sanni B, Williams K, Sokolov EP and Sokolova IM. Effects of acclimation temperature and cadmium exposure on mitochondrial aconitase and LON protease from a model marine ectotherm, Crassostrea virginica. Comp Biochem Physiol C Toxicol Pharmacol. 2008;147 1:101-12. doi:10.1016/j.cbpc.2007.08.005.

116. Bragantini B, Tiotiu D, Rothé B, Saliou JM, Marty H, Cianférani S, et al. Functional and Structural Insights of the Zinc-Finger HIT protein family members Involved in Box C/D snoRNP Biogenesis. Journal of molecular biology. 2016;428 11:2488-506. doi:10.1016/j.jmb.2016.04.028.

117. Waseem M, Aslam MM and Shaheen I. The DUF221 domain-containing (DDP) genes

identification and expression analysis in tomato under abiotic and phytohormone stress. GM crops & food. 2021;12 1:586-99. doi:10.1080/21645698.2021.1962207.

118. Wolmarans A, Lee B, Spyracopoulos L and LaPointe P. The Mechanism of Hsp90 ATPase Stimulation by Aha1. Sci Rep. 2016;6:33179. doi:10.1038/srep33179.

119. Ohtsuka K and Hata M. Mammalian HSP40/DNAJ homologs: cloning of novel cDNAs and a proposal for their classification and nomenclature. Cell stress & chaperones. 2000;5 2:98-112. doi:10.1379/1466-1268(2000)005<0098:mhdhco>2.0.co;2.

120. Zhang G, Fang X, Guo X, Li L, Luo R, Xu F, et al. The oyster genome reveals stress adaptation and complexity of shell formation. Nature. 2012;490 7418:49-54. doi:10.1038/nature11413.

121. Shay JE and Celeste Simon M. Hypoxia-inducible factors: crosstalk between inflammation and metabolism. Seminars in cell & developmental biology. 2012;23 4:389-94. doi:10.1016/j.semcdb.2012.04.004.

Tables and Figures

Figure 1. Maximum likelihood phylogenetic tree constructed by MCMCTree with divergence time estimated among species. Numbers next to the nodes represented the estimated divergence time (million years ago [Ma]). Divergences used for the recalibration of time estimation are indicated with red dots.

Figure 2. Dot plot of genome macrosynteny between littorinids and *P. yessoensis* chromosomes. Each dot represents a common single-copy gene.

Figure 3. Macrosynteny between presumed ancient bilaterian ancestor (ALG), *P. yessoensis* (PY) and littorinid (L) linkage groups, which indirectly shows the chromosome evolution from the 17 ALGs to the 17 Ls.

Table 1 Summary of statistics for the *L. brevicula* and *L. sinensis* genome assembly

	L. brevicula	*L. sinensis*
Main genome scaffold total:	3132	27
Main genome contig total:	3702	935
Main genome scaffold sequence total:	928.20Mb	822.61M
Main genome contig sequence total:	927.93Mb (0.029%gap)	822.51Mb (0.011%gap)
Main genome contig N50	3.43Mb	2.31Mb
Main genome scaffold N50	48.134Mb	32.91Mb

Table 2 Classification of the repetitive elements in *L. brevicula* and *L. sinensis* genome assembly

	Count		Length(bp)		% of genome	
Type	*L. brevicula*	*L. sinensis*	*L. brevicula*	*L. sinensis*	*L. brevicula*	*L. sinensis*
DNA transposons	1,590,204	684,216	204,405,206	98,112,746	22.02	11.93
Retroelements	346,793	302,792	82,326,206	77,246,672	8.87	9.39
Other	1,049,612	1,286,201	91,614,177	119,303,375	9.87	14.5
Unknown	306,488	183,893	60,252,340	43,309,094	6.49	5.27
Total	3,293,097	2,457,102	438,527,929	337,971,887	47.25	41.09

	Gene number	Gene length(bq)	Exon number	Exon length(bp)	CDS number	CDS length(bp)	Intron number	Intron length(bp)
Augustus	34674	295034110	180736	42008685	180736	42008685	146062	284554959
GeneMark	63045	439739035	351385	72352020	351385	72352020	288340	420740249
GlimmerHMM	102779	815976119	442892	84048912	442892	84048912	340113	731927207
snap	106714	433479633	235562	48522306	235562	48522306	128848	408900899
pasa	16814	147049896	75537	21558531	69236	13599948	58723	137473020
BRAKER	62069	412536748	338680	70682528	338582	70657862	276611	427400900
StringTie	22821	352778998	298782	82953013	242022	39915129	275961	936869119
Metaeuk	28140	228895825	122085	30813543	122085	30329379	93945	214937370

Table 3 Statistics of predicted protein-coding genes in the genome assembly of *L. brevicula*

	Gene number	Gene length(bp)	Exon number	Exon length(bp)	CDS number	CDS length(bp)	Intron number	Intron length(bp)
Augustus	35447	259738593	170999	37838537	170999	37838537	135552	250337236
GeneMark	45271	460995069	334280	62885658	334280	62885658	289009	449002676
GlimmerHMM	96017	732805423	433889	80809013	433889	80809013	337872	651996410
snap	89382	103685469	615508	99868582	615508	99868582	526126	102498509
pasa	16432	147973099	77708	24649405	73323	15251451	61276	136974809
BRAKER	16412	223532923	197422	35043360	197422	35043360	181010	390634430
StringTie	23891	377313462	372490	11454963	329559	58392819	348599	127921109
Metaeuk	19668	127670887	64511	18668553	64511	18562146	44843	117829889

Table 4 Statistics of predicted protein-coding genes in the genome assembly of L. sinensis

Click here to access/download;Figure;Figure_1.pdf

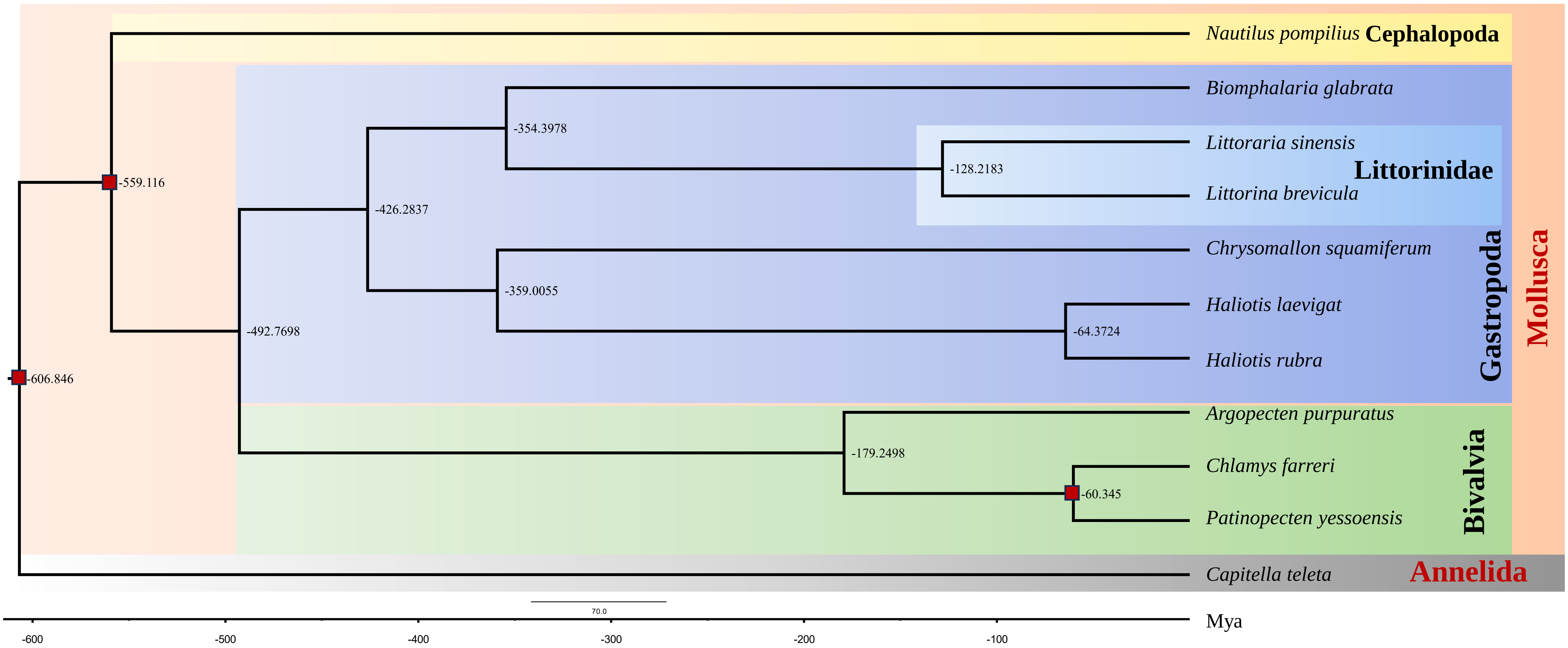

Figure
Click here to access/download;Figure;Figure_2.pdf

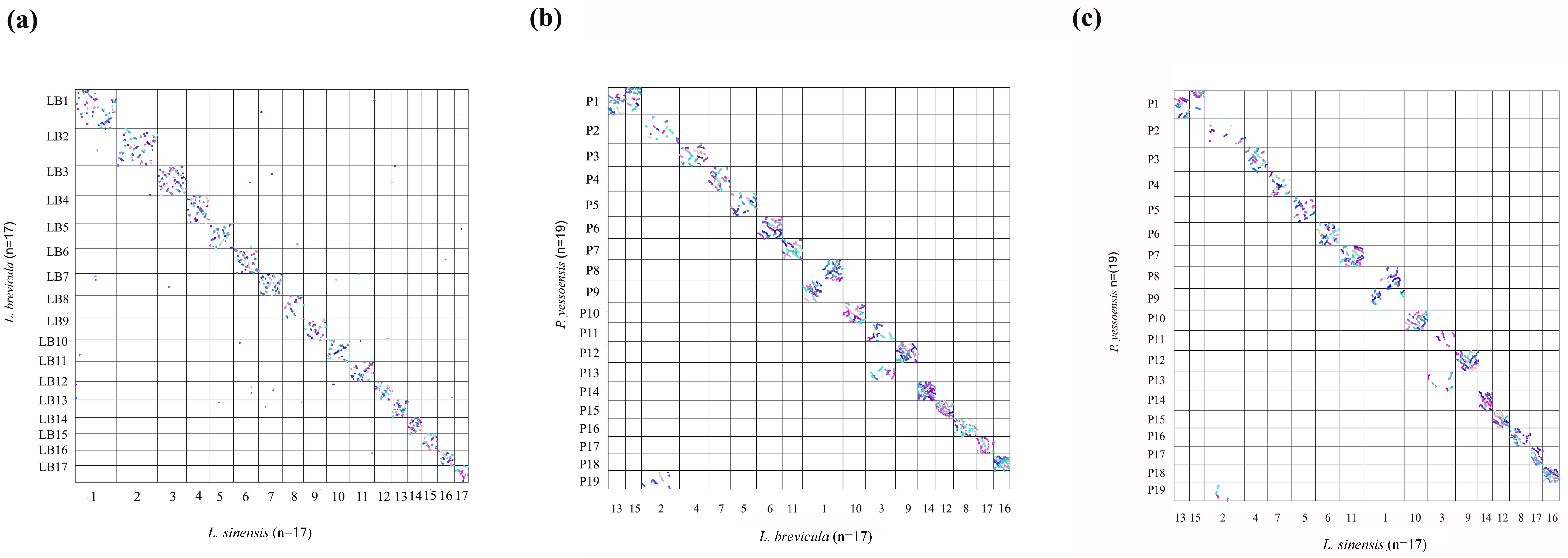

Figure

Click here to access/download;Figure;Figure_3.pdf

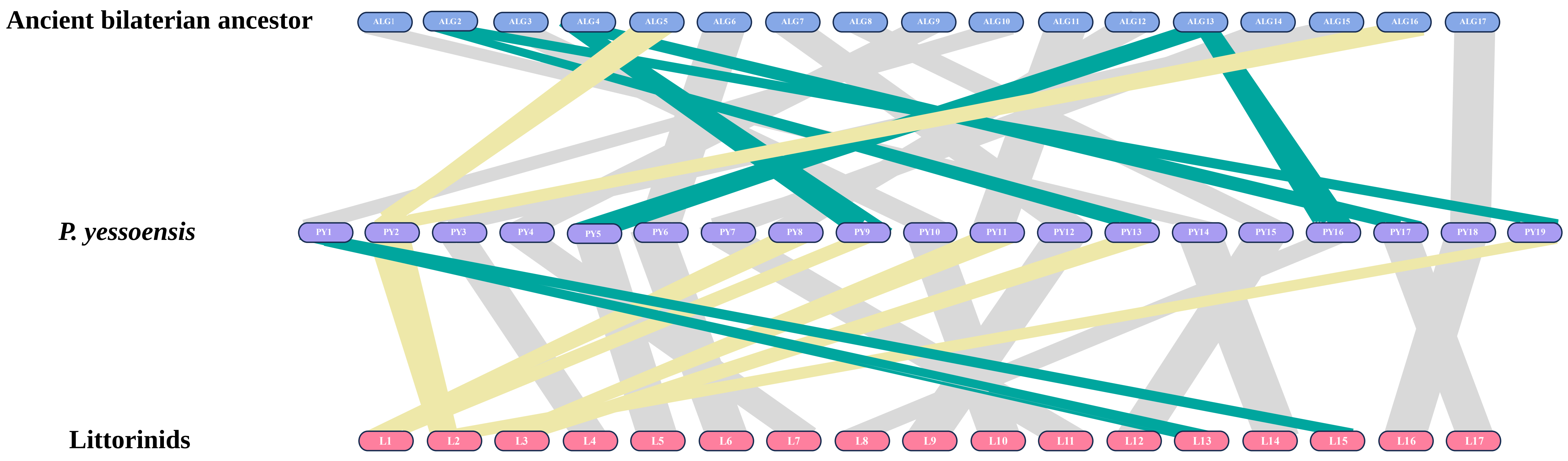

Click here to access/download
Supplementary Material
Supplementary_Material.docx
