# Supplementary material for: Chromosome-level genome assemblies of two littorinid marine snails indicate genetic basis of intertidal adaptation and ancient karyotype evolved from bilaterian ancestors
Source: Gigascience. 2024 Sep 25;13:giae072. doi: 10.1093/gigascience/giae072 (PMC11423352; doi:10.1093/gigascience/giae072)
Supplement: giae072_GIGA-D-24-00090_Revision_1 [file giae072_giga-d-24-00090_revision_1.pdf]

GigaScience

Chromosome-level genome assemblies of two littorinid marine snails indicate genetic basis of intertidal adaptation and ancient karyotype evolved from bilaterian ancestors

--Manuscript Draft--

Manuscript Number:	GIGA-D-24-00090R1
Full Title:	Chromosome-level genome assemblies of two littorinid marine snails indicate genetic basis of intertidal adaptation and ancient karyotype evolved from bilaterian ancestors
Article Type:	Data Note
Funding Information:	National Natural Science Foundation of China (31970488) — Dr. Jin-xian Liu<br>National Natural Science Foundation of China (31972793) — Dr. Dong-Xiu Xue
Abstract:	Living in the intertidal environment with a common karyotype of 17 chromosomes (which is the same with the presumed number of the ancient linkage groups (ALGs) of bilaterian ancestor), littorinid snails are excellent models for understanding the adaptation to harsh fluctuating environments and early evolution of bilaterians. Here, we generated high-quality, chromosome-scale genome assemblies for two littorinid marine snails, Littorina brevicula (927.94Mb) and Littoraria sinensis (882.51Mb) with contig N50 of 3.43Mb and 2.31Mb, respectively. Comparative genomic analyses identified 92 expanded gene families and 85 positively selected genes as potential candidates for intertidal adaptation in the littorinid lineage, which were functionally enriched in stimulus responses, innate immunity and apoptosis processes regulating and might be involved in cellular homeostasis maintenance in the stressful intertidal environments. Genome macrosynteny analyses indicated that 4 fissions and 4 fusions led to the evolution from the 17 presumed bilaterian ancestral chromosomes to the 17 littorinid chromosomes, implying that the littorinid snails have a highly conserved karyotype with the bilaterian ancestor. Three chromosomal fissions and 1 chromosomal fusion from the bilaterian ALGs were shared by the bivalve scallop and gastropoda littorinid snails, indicating that the chromosome-scale ancient gene linkages were generally preserved in the mollusk genomes for over 500 million years. We proposed that, other than the stability of living environments, other evolutionary or developmental constraints could exist on the evolution of genome organization of early bilaterians. The highly conserved karyotype makes the littorinid snail genomes valuable resources for understanding early bilaterian evolution and biology.
Corresponding Author:	Jin-xian Liu<br>Institute of Oceanology Chinese Academy of Sciences<br>Qingdao, CHINA
Corresponding Author Secondary Information:	
Corresponding Author's Institution:	Institute of Oceanology Chinese Academy of Sciences
Corresponding Author's Secondary Institution:	
First Author:	Yan-Shu Wang
First Author Secondary Information:	
Order of Authors:	Yan-Shu Wang
	Meng-Yu Li
	Yu-Long Li
	Yu-Qiang Li
	Dong-Xiu Xue
	Jin-xian Liu

Powered by Editorial Manager® and ProduXion Manager® from Aries Systems Corporation

**Order of Authors Secondary Information:**	
**Response to Reviewers:**	Dear Editor Hongfang Zhang,<br>Thank you for giving us an opportunity to revise our manuscript (GIGA-D-24-00090) to make it acceptable to GigaScience. The comments of the reviewers are all valuable, insightful, and very helpful for revising and improving the manuscript. We have thoroughly revised the manuscript following the recommendations. Please check our responses to the comments as reflected in the revised manuscript and response letter. Our response to the reviewers' comments are listed below.We believe that we have addressed and accommodated the comments to the extent that is reasonable if not exhaustive. Accordingly, we hope that the manuscript can now be accepted for GigaScience.<br>Thank you again for your great editorial efforts.<br>Sincerely yours,<br><br>Jin-Xian Liu<br><br><br>Response to Reviewer 1 Comments<br><br>Response: We would like to thank you for your careful reading, helpful comments, and constructive suggestions, which have significantly improved the presentation of our manuscript.<br>We have carefully considered all comments from the reviewers and revised our manuscript accordingly. The manuscript has also been double-checked, and the typos and grammar errors we found have been corrected. We have simplified the discussion about comparative genomic analysis and revised Fig. 3 to make it clearer to understand. In the following section, we summarize our responses to each comment. We believe that our responses have well addressed all the concerns of the reviewers. We hope our revised manuscript can be accepted for publication.<br><br>P4, bottom - a helpful citation here would be Johannesson et al. 2024 Trends in Genetics<br>Response: We thank the reviewer for providing us with a recent review article supporting the importance that littorinid snails have in speciation and adaption. We have added the citation of the corresponding article to our manuscript at P4 line 6.<br>P5, top - citation 17 does not mention bacteria or viruses and so seems inappropriate here.<br>Response: Thank you for pointing out this problem in the manuscript. We corrected the citation at P4 line 12-13.<br>P5, mid, and P6 - note that a high-quality genome with annotation is now available for Littorina saxatilis: GBE https://doi.org/10.1093/gbe/evae076<br>Response: Thank you for pointing out the issue. Considering the publication of the L. saxatilis genome, we made some revisions in P4 line 20 to P5 line 2.<br>P6, bottom - it is unsafe to claim that gastropods are unchanged over these long time-periods because we know little beyond the form of the shell for early taxa.<br>Response: Thank you for pointing out this problem. We have removed this sentence in the revised manuscript.<br>P8, mid - the papers by Simakov and coworkers show that multiple taxa have karyotypes that can be related to the bilaterian ALGs (indeed, this is how the ALGs were identified). Therefore, the gross similarity of the littorinid LGs with the ALGs does not make littorinids particularly strong models for the study of early bilaterian evolution.<br>Response: Thank you for your suggestion. Macrosynteny analyses showed the karyotype of these two littorinids and bilaterian ancestor might be highly conserved. However, we didn't make a comparation among the correspondence between different taxa and the presumed bilaterian ALGs, so it is improper to consider littorinids as a particularly good model for studying the evolution of early bilaterian ancestor. We have removed this sentence in the revised manuscript.<br>P10, bottom - I would have expected a duplicate removal step in this pipeline although I note that duplicated BUSCOs are not common<br>Response: Thank you for pointing out this issue. After the assembly of two littorinid genomes using Wtdbg2 and NextDenovo, we assessed the genome quality using BUSCO. Just as you mentioned, we found that duplicated BUSCOs were quite scarce in these two genomes (1.2% in L. brevicula and 3.2% in L. sinensis). Therefore we thought that the duplicates might make little difference to the completeness and

Powered by Editorial Manager® and ProduXion Manager® from Aries Systems Corporation

	accuracy for the genome assembly and didn't remove them.<br>P14, top - generic names should be given here in full. Haliotis is the only gastropod in this background set and it is in a different subclass from Littorinidae (common ancestor more than 400Mya). Therefore, there is the opportunity for many differences to have evolved under positive selection that are unrelated to the intertidal environment. The test for positive selection should be adjusted for multiple comparisons since many genes were investigated without a priori predictions.<br>Response: Thank you for the suggestion. We have revised the generic names into full name. Considering the divergence time of the littorinid snails and the closest related lineage was over 400Mya, the genes that was identified to be positively selected may be related with many differences like life-history instead of just intertidal adaptation. So in the revised manuscript, FDR correction was performed on the results ($p < 0.01$) and the positively selected genes were functionally annotated, based on which only 85 genes were considered to be potential candidate genes for intertidal adaptation. The discussion was only about this potential candidate gene set on page 16 line 19 – page 17 line 3.<br><br><br>Table 1 - the meaning of 'Main genome' should be explained in the legend<br>Response: Thank you for pointing out this issue. We directly used the headers from the results generated from the script carelessly. This table shows statistics for the chromosome-level assembly of the two littorinids. We find that the word "main" here is improper and meaningless, so we made a correction and delete it.<br>Fig. 3 - this would be much more legible if LGs were re-ordered, rather than following their (arbitrary) numbering.<br>Response: Thank you for your suggestion. We have re-ordered the bars representing different LGs and made the figure more clear and easier to understand (Figure 3).<br>P16 - either a mutation rate must have been used to infer times, or a calibration point or points, but I did not find the rate or source of calibration in the MS. The split time given here (128My) is much older than in Reid et al 2012 (90-90My).<br>Response: We thank the reviewer for pointing out this issue. Here in our work, 3 calibration points were used to estimate the divergence time for nodes in the phylogenetic tree: the divergence time between Capitella teleta and molluscan species (534.3-654.0 Mya) from citation 75; the divergence time between Nautilus pompilius and Bivalvia and Gastropoda species (527.6-619.1 Mya) from citation 75; the divergence time between Chlamys farreri and Patinopecten yessoensis (46.1-71.7 Mya) from the Timetree database (http://www.timet ree.org/) and citation 76. This part is rewritten in the "Gene family, phylogenetic analysis, and divergence time estimation" section in "Material and Methods" (page 10 line 6-10) in the revised manuscript and three calibration points were marked using red squares in Figure 1. The differences between our estimated divergence time and previous studies were discussed in the paragraph of "Discussion" (page 15 line 17 – page 16 line 9).<br>P17, bottom - 'collinearity' is not a good word here, because of the extensive within-chromosome rearrangements<br>Response: Thank you for pointing out this problem. Considering our macrosynteny analyses didn't show the rearrangements within each chromosome, 'collinearity' seems to be improper here. We have changed the word 'collinearity' into 'correspondence' to describe the synteny between chromosomes, as Wang et al. (2017) used before in citation 41.<br>Figure 3 - a better layout could be found. The current layout risks implying that evolution proceeded from the ALG arrangement, to the PYE arrangement and then to the littorinid arrangement. In reality, there was a step from the ALG to a common molluscan ancestral state and then, independently, to the PYE and littorinid tips. Some features of the ancestral molluscan state can be inferred and so a layout that showed this node would be helpful.<br>Response: Thank you for your suggestion. We have added a new layout representing the common LGs that the scallop and littorinid snails may share in the most recent common ancestor. The evolution is now indicated by arrows while macrosynteny indicating by bold lines (Figure 3).<br>P21, bottom - I don't think that synteny between L. brevicula and L. sinensis is shown at any point (despite mention here).<br>Response: Thank you for pointing out this. Macrosynteny analysis has also been performed between L. brevicula and L. sinensis, of which the result was shown in Figure 2a and detailly described in section "Evolution of littorinid chromosomes from

the ancient bilaterian ancestor” in “Results” (page 14 line 17-19).
P22, top - care is needed here - it is only the gross karyotype that is shown to be 'slow-evolving' and one should not imply slow evolution of any other aspect of gastropod or littorinid biology.
Response: Thank you for pointing out this. On one hand, our macrosynteny analyses didn’t include intra-chromosome rearrangements, on the other hand, it was improper to define the speed of evolution as ‘slow’ or ‘rapid’ without comparation. We have removed this sentence in the revised manuscript.
P22-23 - the Conclusions section largely repeats aspects of the Discussion and is probably not needed.
Response: Thank you for pointing out this. We have deleted this section in the revised manuscript.

Response to Reviewer 2 Comments

Response: We would like to thank you for your careful reading, helpful comments, and constructive suggestions, which have significantly improved the presentation of our manuscript.
We have carefully considered all comments from the reviewers and revised our manuscript accordingly. The manuscript has also been double-checked, and the typos and grammar errors we found have been corrected. The Phred quality scores for ONT long reads were calculated resulting in Q20=74.96% and Q30=74.95%. In the following section, we summarize our responses to each comment. We believe that our responses have well addressed all the concerns of the reviewers. We hope our revised manuscript can be accepted for publication.
- title: I think "reveal" is too strong as the authors have only detected candidate genes and no functional assay has been performed
Response: Thank you for pointing out this. We have changed the word ‘reveal’ into ‘indicate’ since there was no gene functional assay and solid evidence.
- abstract, line 1: delete "evolutionary" before "adaptation"
Response: Thank you for your suggestion. We have removed the word “evolutionary” before “adaptation”.
- abstract, line 1: understanding adaptation to environments and the early evolution of bilaterians are two important aims of evolutionary biology but very different and I am not sure they can be combined in the same sentence.
Response: Thank you for your suggestion. We have changed the beginning of the abstract into ‘Living in the intertidal environment with a common karyotype of 17 chromosomes (which is the same with the presumed number of the ancient linkage groups (ALGs) of bilaterian ancestor), littorinid snails are excellent models for understanding the adaptation to harsh fluctuating environments and early evolution of bilaterians.’ in the revised manuscript page 2 line 2-5.
- abstract, line 8: I find no support in the study to assert that the mentioned gene families "played key roles in adaptation to multiple biotic and abiotic stresses in intertidal environments".
Response: Thank you for pointing out this issue. It is bold to say that these gene families "played key roles in adaptation to multiple biotic and abiotic stresses in intertidal environments" without functional assay. We have changed this sentence into “Comparative genomic analyses identified 92 expanded gene families and 85 potential candidate positively selected genes for intertidal adaptation in the littorinid lineage, which are functionally enriched in stimulus responses, innate immunity and apoptosis processes regulating and may be involved in cellular homeostasis maintenance in stressful intertidal environments.” in page 2 line 8-12.
- Page 3, line 7: "processes" instead of "process"
Response: Thank you for pointing it out. We have changed the word “process” into “processes”.
- Page 3, line 7: "evolve" instead of "acquire"
Response: Thank you for pointing it out. We have changed the word “acquire” into “evolve”.
- Page 3, line12: please sustain better with references that the genetic basis of adaptation to fluctuating environments remains mostly unknown.
Response: Thank you for pointing out this issue. We have rewritten this sentence into “To better understand how they survive in and adapt to fluctuating environments, it is crucial to elucidate the genetic mechanistic basis.” and added two references (citation

	4 and 5) in page 3 line 8-10.<br>- Page 3, line 2 from the end: rhythm? Instead of "rhyme"<br>Response: Thank you for pointing it out. We have changed the word “rhyme” into “rhythm”.<br>- Page 4: This is a data note and in this regard, I find the introduction from this page on rather long and verbose. In page 4, I suggest deleting many sentences and focusing directly on the Littorinidae (line 13).<br>Response: Thank you for pointing out this issue. The manuscript was firstly written as a research article so the introduction is too long for a data note. We have rewritten the beginning of this paragraph and made some simplification about background to focus directly on the littorinid snails at the end of page 3.<br>- Page 5, line 2: rhythm? Instead of "rhyme"<br>Response: Thank you for pointing it out. We have changed the word “rhyme” into “rhythm”.<br>- Page 5, line 6: are really periwinkles the best model to understand adaptation to climate change? Please support this asseveration.<br>Response: Thank you for pointing out this issue. Based on previous reviews and researches, we are not sure if periwinkles are the “best” model for understanding the adaptation to climate change, but considering the steep environmental gradient of intertidal shores, periwinkles are ideal models for elucidating possible responses of organisms to climate, especially temperature changes. Two references were added to support this asseveration in the revised manuscript (page 4 line 9 to 13).<br>- Page 5, line 15 to Page 6, line 7: these sentences can be deleted to maintain the focus of the introduction. Start the paragraph with "Yet, no …"<br>Response: Thank you for pointing out this issue. We have deleted these corresponding sentences and rewritten the paragraph about littorinid genomes since the genome of L. saxatilis was reported in February 2024 (Page 4 line 19 to page 5 line 3).<br>- Page 6, line 4: from the end: I would say that is a key question for evolutionary bioloy not only for the Evo-Devo field.<br>Response: Thank you for this suggestion. We have rewritten this sentence into “Understanding how the enigmatic urbilateria, the last common ancestor of all bilaterians, was constructed is one of the key questions for evolutionary biology.” in page 5 line 4-5.<br>- Page 6, last line: "almost unchanged from their ancestors". This asseveration is simply not true. After hundred million years of evolution, extant gastropods have diverged considerably from their ancestors. The authors may be referring to the external resemblance of shells in extant and fossil snails. Please delete this sentence.<br>Response: Thank you for pointing out this issue. We have removed this sentence in the revised manuscript.<br>- Page 7, line 2: The gastropods are as good as e.g., bivalves, cephalopods, caudofoveates to represent mollusks in comparative studies on the bilaterian ancestor, and mollusks are as good as any other phylum for this purpose. Please delete or rewrite this sentence.<br>Response: Thank you for your suggestion. We have deleted this sentence in the revised manuscript.<br>- Page 7, lines 5-8: These sentences can be deleted to alleviate the introduction.<br>Response: Thank you for your suggestion. We have deleted these sentences in the revised manuscript.<br>- Page 8, line 1: add "including detection of expanding/ contracting gene families and positively selected genes" before "were performed", and the delete sentences from line 2 to 12 (end of introduction).<br>Response: Thank you for your suggestion. We have rewritten the sentence into “To investigate genetic mechanisms associated with adaptation to intertidal harsh and fluctuating environments, comparative genomic analyses including detection of expanding/contracting gene families and positively selected genes were performed. Macrosynteny analysis were also conducted to uncover karyotype evolution from the bilaterian ancestor to mollusks.” in page 6 line 2-6 and made the deletion in the revised manuscript.<br>- Page 8, line 3 from the end: Please go straight to what worked and delete this whole paragraph (that continues in page 9).<br>Response: Thank you for your suggestion. We have removed these corresponding sentences in the first paragraph of “Sampling, genomic DNA extraction and sequencing” and started directly with what worked.<br>- Page 9, line 12: delete "genomic" before "DNA" and add after "from a single

Powered by Editorial Manager® and ProduXion Manager® from Aries Systems Corporation

individual". Please specify the source tissue (foot?)
Response: Thank you for your suggestion. We have rewritten this sentence into “For genomic sequencing of L. brevicula, genomic DNA was extracted using the E.Z.N.A Mollusc DNA Kit from foot muscle tissue of a single individual.” in page 6 line 12-13.
- Page 9, line 13: delete "for genome assembly and correction". Delete "of a Qingdao L. brevicula individual"
Response: Thank you for your suggestion. We have made the deletion and rewritten this sentence into “Genomic DNA was sheared by a g-TUBE device (Catalog No. 520079, Covaris, MA) and then repaired and purified for further PacBio CLR library preparation according to the manufacturer’s protocol (Pacific Biosciences, CA).” in page 6 line 13-16.
- Page 9, line 15: Please specify that you used the CLR and not the HiFi technology of PacBio.
Response: Thank you for your suggestion. We have rewritten this sentence into “Genomic DNA was sheared by a g-TUBE device (Catalog No. 520079, Covaris, MA) and then repaired and purified for further PacBio CLR library preparation according to the manufacturer’s protocol (Pacific Biosciences, CA).” in page 6 line 13-16.
- Page 9, last line and page 10 first paragraph: I suggest first explaining the ONT procedure and then explaining that in order to polish the assemblies, an Illumina sequencing was performed on the DNBSEQ-T7 system. Please specify the source tissue (foot?)
Response: Thank you for your suggestion. We have rewritten this paragraph into “For L. sinensis, genomic DNA from foot muscle tissue of a single snail was extracted using a Genomic-tip 100G (QIANGEN) kit and sheared and size-selected with the aforementioned procedure for the SMRT library. ONT libraries were constructed with these selected fragments using the Ligation Sequencing 1D Kit (Oxford Nanopore, Oxford, UK, p/n SQK-LSK109) according to the manufacturer's instructions. Sequencing was performed on the PromethION (ONT) platform. In order to polish the assembly, genomic DNA was extracted from the foot muscle tissue of the same individual using the E.Z.N.A Mollusc DNA Kit and short-read sequencing of a library with an insert length of ~350bp was performed on the DNBSEQ-T7 system.” on page 6 line 20 to page 7 line 7.
- Page 10, line 10: "The Hi-C reads accounts for ~138X coverage of the L. brevicula genome and~123X coverage…". Perhaps, this sentence should be at the beginning of the results where the authors also mention the coverage of the PacBio and ONT. There, they should also mention the coverage of the Illumina made to polish the ONT assembly.
Response: Thank you for your suggestion. We have removed the description of the coverage of Hi-C sequencing in “Materials and Methods” to section “Genome assembly and annotation of two littorinid snails” in “Results”. The coverage of the DNBSEQ-T7 short reads used for polishing the ONT assembly was also mentioned in “Results” page 12 line 6 (~176-fold coverage).
- Page 11, line 8: Why using ALLHiC for the scaffolding of the L. sinensis genome and not for that L. brevicula?
Response: Thank you for pointing out this issue. The Hi-C reads were firstly anchored to both littorinids using the software 3D-DNA but it didn’t work well with L. sinensis and the resulting assembly was fragmented. Therefore, we used ALLHiC and finally got the satisfying chromosome-level genome assembly for L. sinensis.
- Page 12, line 1: What tissue was the source for the transcriptome?
Response: Thank you for pointing out this issue. The tissue for transcriptome was from the foot muscle tissues of these two littorinids. We have made it clear in the revised manuscript page 8 line 19.
- Page 12, line 10: why not including here any e.g., Lautoconus ventricosus, which also has a chromosome-level genome?
Response: Thank you for pointing out this issue. When we chose the species used in the genome annotation, we directly downloaded data from MolluscDB (http://download.mgbase.qnlm.ac/page/download/downloadall/) which has not included the genomic data of Lautoconus ventricosus. It is regrettable to miss such a well assembled genome.
- Page 12, line 11: "caniculata"
Response: Thank you for pointing out this. We have corrected the name of the apple snail into “Pomacea canaliculata” on page 9 line 8.

- Page 12, line 4 from the end. Why using here a list of species different to that used in

METAEUK? Please explain the rationale behind the selection of species in the different analyses.
Response: Thank you for pointing out this issue. As for homologue-based annotation, we expected to identify as many homologous genes as possible, so the genomic data used here in METAEUK were mainly from gastropoda snails which are supposed to contain more homologous genes with littorinids than those species used for comparative genomic analyses in which we focused on the difference of habitats instead of phylogenetic relationship.
- Page 13, line 9. Three time "estimates" retrieved… were used for "calibration". The Timetree database has estimates from different studies that have performed chronogram inferences based on fossil or geographic data. I would suggest using directly fossils to calibrate the tree. You can check the Paleobiology Database (https://paleobiodb.org/) and see, for example, that there are fossils of Littorina from the middle Triassic of Germany (247 MYA) and of Littoraria from the Danian of Denmark (66 MYA). In any case, if you maintain the estimates from Timetree, please write down the dates used as well as models, priors, and other useful information to interpret the inferred chronogram. Check also (and discuss) the dates inferred in Williams et al. (2003) A molecular phylogeny of the Littorininae (Gastropoda: Littorinidae): unequal evolutionary rates, morphological parallelism, and biogeography of the Southern Ocean. Mol. Phylogenet. Evol. 28: 60-86.
Response: Thank you for pointing out this issue. Here in our work, 3 calibration points were used to estimate the divergence time for nodes in the phylogenetic tree: the divergence time between Capitella teleta and molluscan species (534.3-654.0 Mya) from citation 75; the divergence time between Nautilus pompilius and Bivalvia and Gastropoda species (527.6-619.1 Mya) from citation 75; the divergence time between Chlamys farreri and Patinopecten yessoensis (46.1-71.7 Mya) from the Timetree database (http://www.timet ree.org/) and citation 76 . This part is rewritten in the “Gene family, phylogenetic analysis, and divergence time estimation” section in “Material and Methods” (page10 line 6-10) and three calibration points were marked using red squares in Figure 1. The estimated divergence time of the two littorinids was compared with results from previous studies and discussed in the second paragraph of Discussion.
- Page 14, line 3: It is not clear to me that the comparison of four "submerged" species versus the two periwinkles will be enough to identify genes related to adaptation to the stress associated to low tides. There are too many other life-history differences that may act as confounding factors.
Response: Thank you for pointing out this issue. Considering the divergence time of the littorinid snails and the closest related lineage was over 400Mya, the genes that was identified to be positively selected may be related with many differences like life-history instead of just intertidal adaptation. So in the revised manuscript, FDR correction was performed on the results ($p < 0.01$) and the positively selected genes were functionally annotated, based on which only 85 genes were considered to be potential candidate genes for intertidal adaptation. The discussion was only about this potential candidate gene set on page 16 line 19 – page 17 line 3.
- Page 15. Results are generally well written!
Response: Thank you for your compliment!
- Page 15, line 1: indicate that the PacBio technology is CLR. Add the coverage of the Illumina reads used for polishing the ONT assembly.
Response: Thank you for your suggestion. We have made it clear that the PacBio technology is CLR and added the coverage (~176-fold coverage) of the DNBSEQ-T7 short reads used for polishing the ONT assembly in page 12 line 3-7.
- Page 17, line 1: "might facilitate littorinids to respond to biotic and abiotic stresses and adapt to the intertidal environment" is not a result, should go to discussion.
Response: Thank you for your suggestion. We have deleted this sentence in the “Result” section and rewritten the intertidal environment adaptation part in the “Discussion” section in page 16.
- Page 17, line 7: "as strong candidate genes for intertidal adaptation" to discussion.
Response: Thank you for your suggestion. We have deleted this sentence in the “Results” section and rewritten the intertidal environment adaptation part in the “Discussion” section in page 16.
- Page 18, line 1: "Previous macrosynteny… ALG16 to PY2) to discussion.
Response: Thank you for your suggestion. We have moved this part into Discussion in the revised manuscript.
- Page 18, first line of discussion: rewrite the sentence "Littorinid snails, as candidate

Powered by Editorial Manager® and ProduXion Manager® from Aries Systems Corporation

	ecological and evolutionary models, require urgently to have reference genomes sequenced at high quality".<br>Response: Thank you for your suggestion. We have rewritten this sentence into "As candidate ecological and evolutionary models, high-quality genomes are urgently needed for littorinid snails." in the revised manuscript on page 15 line 5-6.<br>- Page 18, line 3: Any discussion here on the differences between using PacBio vs ONT?<br>Response: Thank you for pointing out this issue. Sequencing of the two littorinid snails was both performed on the PacBio platform while it failed for L. sinensis. DNA library conduction and sequencing of L. sinensis was then performed by multiple flow cells of the ONT platform from which adequate sequencing data was finally generated. We have added the discussion "Considering the higher accuracy of PacBio platform than the ONT platform, long-read sequencing of the two littorinid snails was performed by using the PacBio platform at the beginning. However, PacBio sequencing for L. sinensis failed possibly due to mucopolysaccharides that might block the zero-model waveguides (ZMWs). Genomic library construction and sequencing of L. sinensis was then performed by multiple flow cells of the ONT platform from which adequate sequencing data was finally generated." in the revised manuscript on page 15 line 6-11.<br>- Page 19, line 1: This large paragraph explains the very diverse functions attributed to proteins, whose gene families were found to be expanded in the comparison between submerged and intertidal species. The authors try to show that all these functions might end having a role in counteracting the stress associated to low tides but the paragraph is rather narrative and not very convincing given the design of the comparison and the lack of any direct evidence or functional assay. I think that this paragraph is rather speculative and needs to be toned down.<br>Response: Thank you for pointing out this. This paragraph was too speculative and redundant for a data note. We have simplified the discussion about the expanded gene families of littorinid lineage in page 16.<br>- Page 20, line 14: Again this paragraph is rather speculative and in particular the very last sentence is not sustained by the data, analyses or literature; "the maintenance of cellular homeostasis and repairing of damaged nucleotides and proteins were main strategies for littorinid snails to hinder the cell apoptosis processes caused by environmental stresses and facilitate adaptation to the harsh intertidal environment."<br>Response: Thank you for pointing out this. It was inappropriate to define the main strategies for intertidal environment adaptation based on the results of this manuscript. We have simplified the discussion about the positively selected genes and rewritten this part at the bottom of page 16 and the beginning of page 17.<br>- Page 22, line 7 from the end. "By overcoming technical challenges". I guess no more that those overcome when sequencing the high-quality genomes of e.g., Chrysomalion, Pomacea or Lautoconus. Please delete.<br>Response: Thank you for your suggestion. We have deleted the "Conclusion" section in the revised manuscript according to the suggestion of another reviewer since it largely repeats aspects of the "Discussion".<br>- Page 23, line 1. "and finally facilitated the adaptation to harsh intertidal environments with multiple biotic and abiotic stresses". This is too speculative. Please delete.<br>Response: Thank you for your suggestion. We have deleted the "Conclusion" section in the revised manuscript according to the suggestion of another reviewer since it largely repeats aspects of the "Discussion".<br>- References: I find that for a data note, 121 are too many references. Could the authors try to use less?<br>Response: Thank you for your suggestion. We have made some deletion and only 88 references are reserved in the revised manuscript.<br>- Figure 1: Please add credibility intervals to the date estimates. Please add geological periods in the bottom line.<br>Response: Thank you for your suggestion. We have added credibility intervals to the date estimates and geological periods in the revised Figure 1.<br>- Table 3 &4: These are too technical; perhaps they could be moved to supplementary material<br>Response: Thank you for your suggestion. We have moved these two tables into supplementary material in the revised manuscript.
**Additional Information:**	
**Question**	**Response**

Powered by Editorial Manager® and ProduXion Manager® from Aries Systems Corporation

Are you submitting this manuscript to a special series or article collection?	No
**Experimental design and statistics**<br><br>Full details of the experimental design and statistical methods used should be given in the Methods section, as detailed in our Minimum Standards Reporting Checklist. Information essential to interpreting the data presented should be made available in the figure legends.<br><br>Have you included all the information requested in your manuscript?	Yes
**Resources**<br><br>A description of all resources used, including antibodies, cell lines, animals and software tools, with enough information to allow them to be uniquely identified, should be included in the Methods section. Authors are strongly encouraged to cite Research Resource Identifiers (RRIDs) for antibodies, model organisms and tools, where possible.<br><br>Have you included the information requested as detailed in our Minimum Standards Reporting Checklist?	Yes
**Availability of data and materials**<br><br>All datasets and code on which the conclusions of the paper rely must be either included in your submission or deposited in publicly available repositories (where available and ethically appropriate), referencing such data using a unique identifier in the references and in the "Availability of Data and Materials" section of your manuscript.<br><br>Have you have met the above requirement as detailed in our Minimum	Yes

Standards Reporting Checklist?	

Powered by Editorial Manager® and ProduXion Manager® from Aries Systems Corporation

Manuscript

Click here to access/download;Manuscript;Main_text_GIGA-D-24-00090.docx

Chromosome-level genome assemblies of two littorinid marine snails indicate genetic basis of intertidal adaptation and ancient karyotype evolved from bilaterian ancestors

Yan-Shu Wang[a,b,c,1], Meng-Yu Li[a,b,c,1], Yu-Long Li[a,b,1], Yu-Qiang Li [a,b,c], Dong-Xiu Xue[a,b], Jin-Xian Liu[a,b,2]

[a] CAS Key Laboratory of Marine Ecology and Environmental Sciences, Institute of Oceanology, Chinese Academy of Sciences, Qingdao 266071, China; [b] Laboratory for Marine Ecology and Environmental Science, Qingdao Marine Science and Technology Center, Qingdao 266237, China; [c] University of Chinese Academy of Sciences, Beijing 100049, China.

[1] These authors contributed equally to this work.

[2] To whom correspondence should be addressed.

Email: Jin-Xian Liu jinxianliu@gmail.com

Abstract

Living in the intertidal environment with a common karyotype of 17 chromosomes (which is the same with the presumed number of the ancient linkage groups (ALGs) of bilaterian ancestor), littorinid snails are excellent models for understanding the adaptation to harsh fluctuating environments and early evolution of bilaterians. Here, we generated high-quality, chromosome-scale genome assemblies for two littorinid marine snails, *Littorina brevicula* (927.94Mb) and *Littoraria sinensis* (882.51Mb) with contig N50 of 3.43Mb and 2.31Mb, respectively. Comparative genomic analyses identified 92 expanded gene families and 85 positively selected genes as potential candidates for intertidal adaptation in the littorinid lineage, which were functionally enriched in stimulus responses, innate immunity and apoptosis processes regulating and might be involved in cellular homeostasis maintenance in the stressful intertidal environments. Genome macrosynteny analyses indicated that 4 fissions and 4 fusions led to the evolution from the 17 presumed bilaterian ancestral chromosomes to the 17 littorinid chromosomes, implying that the littorinid snails have a highly conserved karyotype with the bilaterian ancestor. Three chromosomal fissions and 1 chromosomal fusion from the bilaterian ALGs were shared by the bivalve scallop and gastropoda littorinid snails, indicating that the chromosome-scale ancient gene linkages were generally preserved in the mollusk genomes for over 500 million years. We proposed that, other than the stability of living environments, other evolutionary or developmental constraints could exist on the evolution of genome organization of early bilaterians. The highly conserved karyotype makes the littorinid snail genomes valuable resources for understanding early bilaterian evolution and biology.

Keywords: littorinid, chromosomal assembly, intertidal adaptation, karyotype evolution

Introduction

Globally widespread long-term environmental fluctuations result in constant changes to biotic and abiotic conditions (such as climate, nutrition loading, and habitat fragmentation), which act at different spatial scales and can profoundly impact the structure, function, and processes of ecosystems [1-3]. Living organisms that persist in fluctuating environments evolve the ability to tolerate physiological disturbances through a variety of physiological and behavioral responses that allow organisms to maintain homeostasis [4]. To better understand how they survive in and adapt to fluctuating environments, it is crucial to elucidate the genetic mechanistic basis [4, 5].

Interfacing land and sea, rocky intertidal shores are the most common littoral habitats throughout the world [6]. Strongly influenced by both aquatic and terrestrial climatic regimes, the rocky intertidal zone is subject to steep environmental gradients, especially thermal and desiccation stresses that occur at low tide [1, 7, 8], which makes it a natural laboratory for examining relationships between abiotic stresses, biotic interaction and ecological patterns in nature [9-11]. Species in intertidal habitats must adapt to two completely distinct environments because of the daily rhythm of the tides: submersion in the aquatic environment at high tide and emerging into the aerial environment at low tide [12]. From low to high shore levels, environmental pressures become more severe and last longer [7, 13].

The periwinkles or littorinids in the family Littorinidae (Children, 1834) are typical gastropoda organisms inhabiting intertidal environments, which contain at least 18 genera and

200 species [14, 15]. Given their wide distribution and high abundance in rocky intertidal shores with steep environmental gradients, littorinid snails have been established as a model system for studying adaptation, evolution, and speciation [16, 17]. Like those successful and well-known modern model species, the biology, taxonomy, phylogeny, and ecology of littorinid snails have been extensively studied [18-20], establishing a solid foundation for deeper investigation into speciation, sexual selection and adaption to environmental change [21, 22]. *Littorina brevicula* (Philippi, 1844) and *Littoraria sinensis* (Philippi, 1847) are two common littorinid snails widely distributed in the rocky intertidal zone of the northwestern Pacific and are two of the most conspicuous and abundant gastropods in their habitats [23-26]. Regularly exposed to aquatic and desiccative environments due to daily rhythm of the tides, these two high-shore species are under the greatest abiotic stresses such as hyperthermy, desiccation, and hypoxia [6], while biotic stresses from pathogens like bacteria and viruses may also be severe due to herbivory of littorinid snails [27]. Understanding how these littorinid snails adapt to the fluctuating intertidal environments, especially thermal stresses, may be fundamental for understanding how species are likely to respond to climate change [21, 28]. Previous studies have discussed the mechanisms by which littorinid snails adapt to environmental challenges, for example, the tolerance limit of low and high temperature of different littorinid populations and the molecular basis of intertidal adaption from both physiological and genetic aspects [12, 13, 28-30]. High-quality genomes are the base to facilitate littorinid snails to achieve their maximum potential as true ecological and evolutionary models [16]. However, there is only one publicly available high-quality chromosome-level littorinid genome for *Littorina saxatilis* [31]. By using PacBio CLR reads and Hi-C data, Jode et al. (2024) assembled a chromosome-level

L. saxatilis genome spanning 1.35Gb with a scaffold N50 of 67Mb [31], which is much improved than the initial draft genome [32]. More high-quality genomes are still urgently needed for the evolutionary and ecological studies of littorinid snails.

Understanding how the enigmatic urbilateria, the last common ancestor of all bilaterians, was constructed is one of the key questions for evolutionary biology. Gastropods are amongst the oldest known bilaterians to appear in fossil records and the earliest undisputed gastropods date from the Late Cambrian Period, around 500 million years ago [33]. The first unambiguous bilaterian fossil is Kimberella, dating to 555 million years ago, which shows remarkable resemblance to a mollusk [34]. Reconstructing the genome of the urbilaterian ancestors will shed light on our understanding of early bilaterian ancestors and their evolution [35]. Analysis of the evolution of karyotypes has been conducted extensively for bilaterian, metazoan, vertebrate, etc. [35-37]. Cytogenetic analyses and karyotype characterization confirm that the diploid chromosome number of 2n = 34 is common in littorinid snails [15, 38-40], which is the same with the presumed number of the ancient linkage groups (ALGs) of bilaterian ancestor [35], suggesting that the littorinid karyotype may represent the ancient karyotype of bilaterian ancestor to some extent. However, the evolutionary relationships between the 17 littorinid snail chromosomes and the 17 presumed ALGs of bilaterian ancestor are unclear, and the equal chromosome numbers do not necessarily imply 1:1 chromosomal homology. The 19 chromosomes of a bivalve mollusk, the scallop *Patinopecten yessoensis* were confirmed to be highly conserved with the 17 bilaterian ALGs [35, 37, 41]. Macrosynteny analysis between *Patinopecten yessoensis* and littorinid genomes could provide insights into the karyotype evolution from the bilaterian ancestor to mollusks, and evolution of early bilaterian ancestors.

In the present study, we assembled high-quality chromosome-level genomes for two littorinid snails, *Littorina brevicula* and *Littoraria sinensis*. To investigate genetic mechanisms associated with adaptation to intertidal harsh and fluctuating environments, comparative genomic analyses including detection of expanding/contracting gene families and positively selected genes were performed. Macrosynteny analysis were also conducted to uncover karyotype evolution from the bilaterian ancestor to mollusks.

Material and Methods

Sampling, genomic DNA extraction and sequencing

Live specimens of *Littorina brevicula* and *Littoraria sinensis* were collected from the rocky intertidal shore of Huiquan Bay in Qingdao (36°3′26″N,120°20′27″E) in 2019 and 2021 respectively. For genomic sequencing of *L. brevicula*, genomic DNA was extracted using the E.Z.N.A Mollusc DNA Kit from foot muscle tissue of a single individual. Genomic DNA was sheared by a g-TUBE device (Catalog No. 520079, Covaris, MA) and then repaired and purified for further PacBio CLR library preparation according to the manufacturer's protocol (Pacific Biosciences, CA). DNA fragments centered at ~15kb were extracted using BluePippin Systems from Sage Science. Sequencing was performed on the PacBio Sequel II System with the Sequel Sequencing Kit 3.0 following the manufacturer's instructions. Only subreads ≥ 5000bp were included for genome assembly.

For *L. sinensis*, genomic DNA from foot muscle tissue of a single snail was extracted using a Genomic-tip 100G (QIANGEN) kit and sheared and size-selected with the aforementioned

procedure for the SMRT library. ONT libraries were constructed with these selected fragments using the Ligation Sequencing 1D Kit (Oxford Nanopore, Oxford, UK, p/n SQK-LSK109) according to the manufacturer's instructions. Sequencing was performed on the PromethION (ONT) platform. In order to polish the assembly, genomic DNA was extracted from the foot muscle tissue of the same individual using the E.Z.N.A Mollusc DNA Kit and short-read sequencing of a library with an insert length of ~350bp was performed on the DNBSEQ-T7 system.

Genomic DNA was extracted from foot muscle tissue of another individual for both species using E.Z.N.A Mollusc DNA Kit. Hi-C fragment libraries were constructed with insert size ranging from 300bp to 700bp and sequenced on HiSeq X Ten and DNBSEQ-T7 system for *L. brevicula* and *L. sinensis* respectively. Quality control was performed by HiC-Pro v2.8.1 [42]. All the sequencing was performed in the Biomarker Technologies Corporation.

Genome assembly and scaffolding

To assemble the genome of *L. brevicula*, subreads from PacBio sequencing were assembled using Wtdbg2 v2.5 [43] with parameters: "-x sq -g 1g -X 100 -AS2 --node-len 2048 --aln-dovetail 20480". The resulting contigs were polished by GCpp v2.0.2 (https://github.com/PacificBiosciences/gcpp) using PacBio data. Hi-C data were used to anchor contigs onto chromosomes using Juicer v1.6 [44] and 3d-DNA [45]. The chromosomal level genome assembly was further adjusted using Juicebox v1.11.08 [46] and gap-filled with TGS-GapCloser v1.2.0 [47] and then polished again with GCpp v2.02.

The ONT long reads of *L. sinensis* were assembled using NextDenovo v2.4.0

(https://github.com/Nextomics/NextDenovo) with parameters: "read_cutoff = 1k, genome_size = 1g". The assembly was first polished using PEPPER v0.1 (https://github.com/kishwarshafin/pepper/tree/r0.1/models) with ONT long reads. Then DNBSEQ-T7 short reads were aligned to the contigs and single base errors were corrected by FREEBAYES v1.3.4 (https://github.com/freebayes/freebayes/releases/tag/v1.3.4) and PILON v1.2.3 [48]. The genome contigs were scaffolded into chromosomes with Hi-C reads using ALLHiC v0.9.8 [49]. The chromosomal level genome assembly was further adjusted using Juicebox v1.11.08 [46] and gap-filled with TGS-GapCloser v1.2.0 [47] and then polished again with DNBSEQ-T7 reads.

To assess the genome quality, the completeness of the two genomes was assessed by BUSCO v5.2.1 [50] using the metazoan (metazoa_odb10) database which contains 954 highly conserved single-copy core genes.

Genome annotation

The repeat library was constructed by RepeatModeler v2.0.1 [51] and EDTA v2.0.1 [52] while RepeatMasker v4.1.2 [53] was used to identify and mask repetitive elements. Based on the repeat-masked genomes, protein-coding genes were predicted using a combination of three approaches: transcriptome-based, de novo, and homologue-based methods. Firstly, transcripts from the foot muscle tissue of the two snails were assembled for transcriptome-based annotation. Illumina short reads of both littorinid snails and full-length PacBio Iso-Seq reads for *L. brevicula* were assembled using Trinity v2.11.0 [54] and ISOSEQ v3

(https://github.com/PacificBiosciences/IsoSeq) respectively and then mapped to the reference genome using MINIMAP2 v2.17 [55]. PASAPIPELINE v2.4.1 [56], STRINGTIE v2.2.1 [57], and TRANSDECODER v5.5.0 (http://transdecoder.sourceforge.net) were used to predict candidate protein-coding regions. Secondly, de novo gene prediction was performed using AUGUSTUS 3.4.0 [58], BRAKER v2.1.6 [59], and GENEMARK v4.69 [60]. Thirdly, METAEUK [61] was used for homologous gene annotation with protein sequences of the following eight species: *Lottia gigantea*, *Haliotis discus hannai*, *Elysia chlorotica*, *Biomphalaria glabrata*, *Aplysia californica*, *Pomacea canaliculata*, *Octopus bimaculoides*, and *Octopus minor* (Table S1). Finally, the results from the three approaches were integrated using EVidenceModeler v2.0.0 [62] and Funannotate v1.8.15 (https://github.com/nextgenusfs/funannotate). For the prediction of gene function, the predicted protein-coding genes were aligned to the databases of UniProt [63], Pfam-A [64], EggNOG [65], MEROPS [66], CAZYme [67], BUSCO [50], and InterProScan [68].

Gene family, phylogenetic analysis, and divergence time estimation

Protein-coding sequences of *Argopecten purpuratus*, *Biomphalaria glabrata*, *Chlamys farreri*, *Chrysomallon squamiferum*, *Haliotis laevigata*, *Haliotis rubra*, *Nautilus pompilius*, *Patinopecten yessoensis*, *L. brevicula*, *L. sinensis* and *Capitella teleta* (outgroup) (Table S1) were aligned using DIAMOND v2.0.14.152 [69] with a cutoff e-value of 1e-5 and compared using OrthoFinder v2.5.5 [70] to construct gene families.

To infer the phylogenetic relationships, 829 single-copy gene families from all 11 species were extracted to perform multiple alignments using MAFFT v7.429 [71] with default

parameter settings. After transformed back to coding DNA and refined by using Gblocks v0.91b [72], all of the alignments were combined into a supergene. The phylogenetic tree was constructed based on the maximum likelihood method in IQ-TREE v1.6.12 [73] with the GTR+F+I+G4 model. Clade support was assessed using bootstrapping algorithm with 1,000 replicates. The divergence time between each clade was estimated with MCMCTree in PAML v4.9 [74]. Three time calibration points were used to estimate the divergence times in the phylogenetic tree: the divergence time between *Capitella teleta* and molluscan species (534.3-654.0 Mya) [75], the divergence time between *Nautilus pompilius* and Bivalvia and Gastropoda (527.6-619.1 Mya) [75], the divergence time between *Chlamys farreri* and *Patinopecten yessoensis* (46.1-71.7 Mya) [76] (http://www.timet ree.org/).

Expansion and contraction of gene families

The CAFE v5 tool [77] was used to examine gene family expansion and contraction with parameter "-p -k 1". Based on a stochastic birth and death model with the lambda option [78], the size difference of each gene family was checked along each lineage of the phylogenetic tree. A probabilistic graphic model was applied to calculate the probability of transitions in gene family size from parent to child nodes. The corresponding *p*-values were calculated for each lineage based on conditional likelihood. Gene families with a *p*-value ≤ 0.05 were considered to be significantly expanded/contracted and were further subjected to GO functional enrichment analyses using the topGO R package [79].

Identification of positively selected genes potentially related to intertidal adaptation of littorinid snails

To identify genes under positive selection in the common ancestor of two littorinid snails (foreground branch), four submerged molluscan species (*Argopecten purpuratus*, *Chlamys farreri*, *Patinopecten yessoensis,* and *Haliotis laevigate*) were used as background branches. These four species inhabit relatively stable sea bottoms and are vulnerable to environmental fluctuation. Single-copy orthologous gene families were extracted and an unrooted phylogenetic tree was constructed using the methods mentioned above, based on which CODMEL of PAML package v4.9 [74] was used to identify genes under positive selection in the foreground branch using the branch-site model. FDR correction was performed on the results and genes were identified as positively selected according to the adjusted *p*-value ($p < 0.01$) and containing amino acid sites with a BEB higher than 99%.

Macrosynteny analyses

Chromosome-scale synteny analyses were performed pairwisely for *L. brevicula*, *L. sinensis,* and *P. yessoensis*. Protein sequences of single-copy gene families were aligned to each other using DIAMOND v2.0.14.152 with parameter "-k1". The macrosynteny analyses were conducted using the MCScanX [80] package with defaulting parameters. The results were then visualized into dot plot figures using the VGSC Java package (https://dvb.ac.cn/vgsc2/service/home.php).

Results

Genome assembly and annotation for two littorinid snails

The PacBio CLR sequencing and ONT sequencing generated a total of 197.66Gb (~212-fold coverage) and 16.58Gb (~20-fold coverage) clean data for *L. brevicula* and *L. sinensis*, respectively. The accuracy of ONT long reads was estimated with Phred quality scores (Q20=74.96% and Q30=74.95%). The DNBSEQ-T7 system generated 145G (~176-fold coverage) clean short reads for the assembly polish of *L. sinensis*. To construct chromosome-level genome assemblies, 129.31Gb (~138X) and 114.67Gb (~123X) clean Hi-C reads were obtained for *L. brevicula* and *L. sinensis*, with 92.21% and 99.98% assembled sequences of *L. brevicula* and *L. sinensis* anchored onto 17 pseudochromosomes (Table S2), which is consistent with previous karyotype analysis [15]. Finally, chromosome-level genome assemblies spanning 927.94Mb for *L. brevicula* and 822.51Mb for *L. sinensis* were obtained, with contig N50 of 3.43Mb and 2.31Mb (Table 1). The BUSCO results indicated high genome assembly completeness, with 888 (93.1%) and 894 (93.8%) out of 954 metazoan single-copy core genes present in the genome assemblies of *L. brevicula* and *L. sinensis* (Table S3).

Repetitive elements composed 47.25% (438.52Mb) and 41.09% (337.97Mb) of the genome for *L. brevicula* and *L. sinensis* respectively (Table 2). A total of 29,335 and 25,386 genes were predicted for *L. brevicula* and *L. sinensis*. The gene number, gene length, coding sequence (CDS) number, as well as lengths of CDS, intron, and exon were described in Table S5&6. A total of 25,495 (86.91%) and 23,238 (91.54%) genes were functionally annotated for *L. brevicula* and *L. sinensis*.

Gene family, phylogenetic, and divergence analyses

A total of 267,386 (89.99%) genes were assigned to 29,488 orthologous groups, of which 5,017 were shared among all 11 species and 2,950 were specific to the two littorinid snails (Table S7). Functional annotation and GO enrichment analysis showed that these littorinid-specific gene families were involved in 142 GO terms relevant to metabolic processes, antioxidant responses, and innate immunity, etc. (Table S8).

Based on 829 single-copy gene families, the species tree for 10 mollusks was constructed using *Capitella teleta* as the outgroup (Figure 1). The divergence time between *L. brevicula* and *L. sinensis* was estimated to be ~128.2 million years, suggesting a deep divergence between the two littorinid snails, yet they shared highly conserved macrosynteny (see below).

Genetic mechanisms of adaptation to the intertidal environment

A total of 92 significantly expanded gene families (involving 897 genes) and 15 contracted gene families (involving 11 genes) were identified for the common littorinid ancestor of *L. brevicula* and *L. sinensis*. The significantly expanded gene families were mainly involved in innate immunity, metabolic processes, stimulus responses, antioxidant responses, etc. (Table S9). For example, carbohydrate hydrolase and triglyceride-related gene families related with energy metabolism, gene families encoding cytochrome P450 (CYP450) and glutathione S-transferases (GSTs) with known functions in constituting the xenobiotic detoxification system of mollusks [81], and defense gene sets like HEPN domain-containing proteins and Sacsin which contained Hsp90-like domains and recruited Hsp70 [82] were found to be expanded in

the littorinid lineage and might facilitate adaptation to harsh intertidal environments. Moreover, gene families encoding multiple pattern recognition receptors (PRRs) expanded the most among all the 92 littorinid expanded gene families, which contained C-type lectin-related proteins (CREPs), fibrinogen-related proteins (FREPs), scavenger receptor cysteine-rich proteins (SRCRs), G-protein coupled receptors (GPCRs), etc. These PRRs constructed the innate immune system of littorinid snails and might play important roles in pathogen defense.

A total of 501 positively selected genes were identified (p-value ≤ 0.01) for the common ancestor of *L. brevicula* and *L. sinensis*. The functions of these genes were annotated using databases mentioned in section 2.3 and further confirmed using GeneCards database [83]. Compared to the littorinid-specific and expanded gene families, functions of the positively selected genes were more unified, which were mainly associated with nucleotide or protein binding.

Evolution of littorinid chromosomes from the ancient bilaterian ancestor

Genome macrosynteny analyses independent of intra-chromosomal rearrangements were performed pairwisely among the two littorinid snails and the scallop *P. yessoensis* using orthologous single-copy genes. The results showed a near-perfect correspondence between chromosomes of *L. brevicula* and *L. sinensis* with few inter-chromosomal rearrangements (Figure 2a). Meanwhile, the correspondence between littorinids and the scallop indicated that *P. yessoensis* chromosomes PY8 and PY9 were homologous to littorinid chromosome L1; PY2 and PY19 were homologous to L2; PY11 and PY13 were homologous to L3; PY1 was

homologous to L13 and L15, resulting in the difference of chromosome numbers between littorinid snails (n = 17) and *P. yessoensis* (n = 19) (Figure 2b,c).

Discussion

As candidate ecological and evolutionary models, high-quality genomes are urgently needed for littorinid snails. Considering the higher accuracy of PacBio platform than the ONT platform, long-read sequencing of the two littorinid snails was performed by using the PacBio platform at the beginning. However, PacBio sequencing for *L. sinensis* failed possibly due to mucopolysaccharides that might block the zero-model waveguides (ZMWs). Genomic library construction and sequencing of *L. sinensis* was then performed by multiple flow cells of the ONT platform from which adequate sequencing data was finally generated. Here, we generated chromosome-scale genome assemblies for two littorinid marine snails. Assessment and comparation with other published molluscan genomes showed high level of continuity and completeness but moderate level of size and repetitive elements for the two littorinid genomes (Table S4), which ensure the accuracy of comparative genomic analyses in our study and provide qualified genomic materials for further molecular ecology and evolution researches.

The fossil record of littorinid snails is incomplete because of poor conditions for preservation on intertidal rocky shores [14, 84, 85], which leads to difficulty in time calibration for the divergence time estimation. In the present study, the estimated divergence time of these two littorinid snails was about 128.22 My. According to Williams et al. (2003), the estimated age of Littorininae is at least Lower Cretaceous (115-190 Mya) [14]. Therefore, the split of the

genus *Littorina* and *Littoraria* might happen not long after the origin of Littorininae. However, the estimated divergence time between the two littorinid snails was larger than that in Reid et al. (2012) (90-95 Mya) based on combined phylogenetic analysis of 28S rRNA, 12S rRNA and cytochrome oxidase c subunit I genes [84]. The accuracy of phylogenetic divergence time estimation based on the whole genome data was supposed to be higher than fragmented sequences of several loci. However, it has also been suggested that there is methodological bias towards overestimation of time based on molecular divergence [86]. So, more littorinid reference genomes are still needed for accurate divergence time estimation among different littorinid genera.

The intertidal rocky shores are characterized with multiple biotic and abiotic environmental stresses that affect the cellular homeostasis of living organisms. Comparative genomic analysis indicated possible genetic adaptation strategies of littorinid snails to the intertidal environment. The expanded energy metabolism gene families, which may facilitate organisms generating ATP to compensate for extra energy demands, are known as key factors in establishing limits of environmental stress tolerance [87]. Genes like CYP450 and GSTs might help littorinid snails to withstand pollutants by detecting and binding with organic and inorganic toxicants [81]. The expanded genes associated with innate immunity might play a key role in adaptation to severe biotic stresses (e.g. virus, bacteria, and parasites) by recognizing and eliminating pathogen through phagocytosis [88]. A total of 85 positively selected genes were identified (Table S10) as potential candidates for intertidal adaptation, and almost half of which were related to nucleotide and protein binding processes and involved in damaged DNA/RNA/protein repairment or degradation. These results suggested that maintenance of

cellular homeostasis and repairing of damaged nucleotides and proteins might be essential to hinder cell apoptosis processes caused by environmental stresses, which could help littorinid snails to adapt to or even thrive in the harsh intertidal environment.

Previous macrosynteny analyses revealed that *P. yessoensis* possessed a highly conserved 19-chromosome karyotype similar to that of bilaterian ancestors [37, 41] and the 19 scallop chromosomes evolved from the 17 presumed ancient linkage groups (ALGs) of bilaterian ancestors through 3 chromosomal fissions (ALG13 to PY5 and PY16; ALG4 to PY9 and PY17; ALG2 to PY13 and PY19) and 1 fusion (ALG5 and ALG16 to PY2). Therefore, the evolutionary trajectory from the 17 ALGs of bilaterian ancestors to the 17 littorinid chromosomes can be inferred based on the macrosynteny analyses: 1) ALG2 fissioned into ALG2-1and ALG2-2; ALG4 fissioned into ALG4-1and ALG4-2; 2) ALG2-1 fused with ALG5 and ALG16 into L2; ALG2-2 fused with ALG11 into L3; ALG4-1 fused with ALG12 into L1; 3) ALG13 fissioned into L5 and L8; ALG10 fissioned into L13 and L15, which indicated that the 17 chromosomes of littorinids evolved from the 17 ALGs of bilaterian ancestors through 4 chromosomal fusions and 4 fissions regardless of intrachromosomal rearrangements (Figure 3).

Although the 17 littorinid chromosomes did not possess a complete ‘1 to 1’ conserved model with the 17 presumed bilaterian ALGs, our analyses revealed that most littorinid chromosomes (9) directly inherited ancient bilaterian gene linkages while the other 8 chromosomes evolved from 4 chromosomal fissions and 4 fusions (Figure 3). Surprisingly, all of the 3 chromosomal fissions and 1 chromosomal fusion between the bilaterian ancestors and *P. yessoensis* were also found between littorinid snails and the bilaterian ancestors, which implied that they occurred before the bivalve-gastropod split (Figure 3) around 500 million

years ago. Overall, the level of chromosome preservation was comparable for the scallop lineage and the littorinid lineage. Considering the less and simpler chromosome evolutionary events that *P. yessoensis* experienced than littorinid snails, the karyotype of *P. yessoensis* might represent the karyotype of the common ancestor of scallop and littorinid snails. Considering the sister relationship between Bivalvia and Gastropoda, these results demonstrated that the chromosome-scale ancient gene linkages were generally preserved in the mollusk genomes over 500 million years, which added evidence to the conclusion that slow chromosome evolution was widespread among invertebrates [36]. Wang et al. [57] proposed that the remarkable conservation of ancestral features in scallop genome is probably as a consequence of life on cold and stable deep-ocean bottoms. However, although the littorinid snails live in the harsh and highly fluctuating intertidal environments, they still have high level of chromosome preservation with the bilaterian ancestors, which is similar to that of scallop. The results implied that living environments might not be the key driver of karyotype evolution in mollusks, other evolutionary or developmental constraints on the evolution of genome organization could exist.

Acknowledgements

This work was supported by the National Natural Science Foundation of China (Grant Nos. 31970488, 31972793).

Additional Files

Supplementary Table S1. Metazoan genome assemblies and gene models used in this study.

Supplementary Table S2. Statistics of chromosomal level assembly of the two littorinid snails.

Supplementary Table S3. Completeness assessment of the two littorinid snails by BUSCO.

Supplementary Table S4. Genome size and repetitive elements of 46 mollusks.

Supplementary Table S5 Statistics of predicted protein-coding genes in the genome assembly of *L. brevicula*.

Supplementary Table S6 Statistics of predicted protein-coding genes in the genome assembly of *L. sinensis*.

Supplementary Table S7. Statistics of gene families of 11 species in comparative genomic analysis.

Supplementary Table S8. Gene ontology of the littorinid-specific gene families.

Supplementary Table S9. Gene ontology of the expanded gene families of littorinid ancestor.

Supplementary Table S10. Potential candidate intertidal adaptation-related genes under positive selection.

Abbreviations

ALG: ancient linkage group; BEB: Bayesian and empirical Bayes approach; bp: base pairs; BUSCO: Benchmarking Universal Single-Copy Orthologs; Gb: gigabase pairs; GO: Gene Ontology; HEPN: higher eukaryotic and prokaryotic nucleasedomains; HSP: heat shock protein; HiC: high-throughput/resolution chromosome conformation capture; kb: kilobase pairs; Mb:

megabase pairs; Mya: million years ago; NCBI: National Center for Biotechnology Information; ONT: Oxford Nanopore Technologies; Pacbio: Pacific Biosciences.

Author Contributions

J.-X. L. conceived and supervised the study; Y.-S. W., M. -Y. L. performed the research; Y.-S. W., M. -Y. L. and Y.-L. L. analyzed the data; Y.-S. W. and J.-X. L. wrote the manuscript. All authors discussed the results and commented on the manuscript.

Competing Interests

The authors declare that they have no competing interests.

Data Availability

The sequencing data that support the findings of this study are openly available in the NCBI Sequence Read Archive (SRA) under BioProject accession number PRJNA1032305 (*Littorina brevicula*) and PRJNA1032307 (*Littoraria sinensis*). The genome assembly and annotation data of *Littorina brevicula* (https://figshare.com/s/74caa5554fc13b9910bb) and *Littoraria sinensis* (https://figshare.com/s/c0d47d3fb2ed21567698) have been deposited in FigShare.

References

1. Chemello S, Vizzini S and Mazzola A. Regime shifts and alternative stable states in intertidal rocky habitats: State of the art and new trends of research. Estuarine, Coastal

and Shelf Science. 2018;214:57-63. doi:10.1016/j.ecss.2018.09.013.

2. Bernhardt JR, O'Connor MI, Sunday JM and Gonzalez A. Life in fluctuating environments. Philos Trans R Soc Lond B Biol Sci. 2020;375 1814:20190454. doi:10.1098/rstb.2019.0454.

3. Vasseur DA and McCann KS. The Impact of Environmental Variability on Ecological Systems. Springer, Dordrecht; 2007.

4. Blewett TA, Binning SA, Weinrauch AM, Ivy CM, Rossi GS, Borowiec BG, et al. Physiological and behavioural strategies of aquatic animals living in fluctuating environments. J Exp Biol. 2022;225 9 doi:10.1242/jeb.242503.

5. Wang X, Cong R, Li A, Wang W, Zhang G and Li L. Transgenerational effects of intertidal environment on physiological phenotypes and DNA methylation in Pacific oysters. Sci Total Environ. 2023:162112. doi:10.1016/j.scitotenv.2023.162112.

6. Thompson RC, Crowe TP and Hawkins SJ. Rocky intertidal communities: past environmental changes, present status and predictions for the next 25 years. Environmental Conservation. 2002;29 2:168-91. doi:10.1017/s0376892902000115.

7. Raffaelli D and Hawkins S. Intertidal Ecology. 1 ed.: Springer, Dordrecht; 1996.

8. Helmuth B, Mieszkowska N, Moore P and Hawkins SJ. Living on the Edge of Two Changing Worlds: Forecasting the Responses of Rocky Intertidal Ecosystems to Climate Change. Annual Review of Ecology, Evolution, and Systematics. 2006;37 1:373-404. doi:10.1146/annurev.ecolsys.37.091305.110149.

9. Bertness MD, Leonard GH, Levine JM and Bruno JF. Climate-driven interactions among rocky intertidal organisms caught between a rock and a hot place. Oecologia.

1999;120 3:446-50. doi:10.1007/s004420050877.

10. Connell JH. Community Interactions on Marine Rocky Intertidal Shores. 1972;3 1:169-92. doi:10.1146/annurev.es.03.110172.001125.

11. Somero GN. Thermal Physiology and Vertical Zonation of Intertidal Animals: Optima, Limits, and Costs of Living1. Integrative and Comparative Biology. 2002;42 4:780-9. doi:10.1093/icb/42.4.780 %J Integrative and Comparative Biology.

12. Storey KB, Lant B, Anozie OO and Storey JM. Metabolic mechanisms for anoxia tolerance and freezing survival in the intertidal gastropod, Littorina littorea. Comp Biochem Physiol A Mol Integr Physiol. 2013;165 4:448-59. doi:10.1016/j.cbpa.2013.03.009.

13. Sokolova IM and Portner HO. Physiological adaptations to high intertidal life involve improved water conservation abilities and metabolic rate depression in Littorina saxatilis. Mar Ecol Prog Ser. 2001;224:171-86. doi:DOI 10.3354/meps224171.

14. Williams ST, Reid DG and Littlewood DT. A molecular phylogeny of the Littorininae (Gastropoda: Littorinidae): unequal evolutionary rates, morphological parallelism, and biogeography of the Southern Ocean. Mol Phylogenet Evol. 2003;28 1:60-86. doi:10.1016/s1055-7903(03)00038-1.

15. Garcia-Souto D, Alonso-Rubido S, Costa D, Eirin-Lopez JM, Rolan-Alvarez E, Faria R, et al. Karyotype Characterization of Nine Periwinkle Species (Gastropoda, Littorinidae). Genes (Basel). 2018;9 11 doi:10.3390/genes9110517.

16. Rolán-Alvarez E, Austin C and Boulding E. The Contribution of the Genus Littorina to the Field of Evolutionary Ecology. Oceanography and marine biology. 2015;53:157-

214. doi:10.1201/b18733-6.

17. Ng TPT, Lau SLY, Seuront L, Davies MS, Stafford R, Marshall DJ, et al. Linking behaviour and climate change in intertidal ectotherms: insights from littorinid snails. Journal of Experimental Marine Biology and Ecology. 2017;492:121-31. doi:10.1016/j.jembe.2017.01.023.

18. Johannesson K. What can be learnt from a snail? Evolutionary Applications. 2016;9 1:153-65. doi:10.1111/eva.12277.

19. Johannesson K, Panova M, Kemppainen P, Andre C, Rolan-Alvarez E and Butlin RK. Repeated evolution of reproductive isolation in a marine snail: unveiling mechanisms of speciation. Philosophical Transactions of the Royal Society B-Biological Sciences. 2010;365 1547:1735-47. doi:10.1098/rstb.2009.0256.

20. Johannesson K. Evolution in Littorina: ecology matters. Journal of Sea Research. 2003;49 2:107-17. doi:10.1016/s1385-1101(02)00218-6.

21. Ravinet M. Notes from a snail island: Littorinid evolution and adaptation. Mol Ecol. 2018;27 13:2781-9. doi:10.1111/mec.14730.

22. Johannesson K, Faria R, Le Moan A, Rafajlović M, Westram AM, Butlin RK, et al. Diverse pathways to speciation revealed by marine snails. Trends in Genetics. 2024;40 4:337-51. doi:10.1016/j.tig.2024.01.002.

23. Li YQ, Li MY, Xing TF and Liu JX. Resolving the origins of invertebrate colonists in the Yangtze River Estuary with molecular markers: Implications for ecological connectivity. Ecol Evol. 2021;11 20:13898-911. doi:10.1002/ece3.8095.

24. Li M, Li Y, Xing T, Li Y and Liu J. Microsatellite marker development and population

genetic analysis revealed high connectivity between populations of a periwinkle Littoraria sinensis (Philippi, 1847). Journal of Oceanology and Limnology. 2022;40 3:1097-109. doi:10.1007/s00343-021-1079-9.

25. Okutani T. Marine mollusks in Japan. In: 2000.

26. Reid DG. Systematics and evolution of Littorina. London :: Ray Society, 1996.

27. Cortez T, Amaral RV, Sobral-Souza T and Andrade SCS. Genome-wide assessment elucidates connectivity and the evolutionary history of the highly dispersive marine invertebrate Littoraria flava (Littorinidae: Gastropoda). Biological Journal of the Linnean Society. 2021;133 4:999-1015. doi:10.1093/biolinnean/blab055.

28. Dong YW, Liao ML, Han GD and Somero GN. An integrated, multi-level analysis of thermal effects on intertidal molluscs for understanding species distribution patterns. Biol Rev Camb Philos Soc. 2022;97 2:554-81. doi:10.1111/brv.12811.

29. Chiba S, Iida T, Tomioka A, Azuma N, Kurihara T and Tanaka K. Population divergence in cold tolerance of the intertidal gastropod Littorina brevicula explained by habitat-specific lowest air temperature. Journal of Experimental Marine Biology and Ecology. 2016;481:49-56. doi:10.1016/j.jembe.2016.04.009.

30. Stankowski S, Zagrodzka ZB, Garlovsky MD, Pal A, Shipilina D, Castillo DG, et al. The genetic basis of a recent transition to live-bearing in marine snails. 2024;383 6678:114-9. doi:doi:10.1126/science.adi2982.

31. Jode AD, Faria R, Formenti G, Sims Y, Smith TP, Tracey A, et al. Chromosome-scale genome assembly of the rough periwinkle Littorina saxatilis. 2024:2024.02.01.578480. doi:10.1101/2024.02.01.578480 %J bioRxiv.

32. Westram AM, Rafajlović M, Chaube P, Faria R, Larsson T, Panova M, et al. Clines on the seashore: The genomic architecture underlying rapid divergence in the face of gene flow. Evolution Letters. 2018;2 4:297-309. doi:10.1002/evl3.74.

33. Boardman RS, Cheetham, A. H., and Rowell, A. J. Fossil Invertebrates. Boston: Blackwell Scientific Publications; 1987.

34. Fedonkin M and Waggoner B. Fedonkin, M. A. & Waggoner, B. M. The Late Precambrian fossil Kimberella is a mollusc-like bilaterian organism. Nature 388, 868-871. Nature. 1997;388:868-71. doi:10.1038/42242.

35. Simakov O, Marletaz F, Cho SJ, Edsinger-Gonzales E, Havlak P, Hellsten U, et al. Insights into bilaterian evolution from three spiralian genomes. Nature. 2013;493 7433:526-31. doi:10.1038/nature11696.

36. Simakov O, Bredeson J, Berkoff K, Marletaz F, Mitros T, Schultz DT, et al. Deeply conserved synteny and the evolution of metazoan chromosomes. Science Advances. 2022;8 5 doi:ARTN eabi5884

10.1126/sciadv.abi5884.

37. Simakov O, Marletaz F, Yue JX, O'Connell B, Jenkins J, Brandt A, et al. Deeply conserved synteny resolves early events in vertebrate evolution. Nat Ecol Evol. 2020;4 6:820-30. doi:10.1038/s41559-020-1156-z.

38. JANSON K. CHROMOSOME NUMBER IN TWO PHENOTYPICALLY DISTINCT POPULATIONS OF LITTORINA SAXATILIS OLIVI, AND IN SPECIMENS OF THE LITTORINA OBTUSATA (L.) SPECIES-COMPLEX. Journal of Molluscan Studies. 1983;49 3:224-7. doi:10.1093/oxfordjournals.mollus.a065716 %J Journal of

Molluscan Studies.

39. Libertini A, Trisolini R and Edmands S. A cytogenetic study of the periwinkle Littorina keenae Rosewater, 1978 (Gastropoda: Littorinidae). Journal of Molluscan Studies. 2004;70 3:299-301. doi:10.1093/mollus/70.3.299 %J Journal of Molluscan Studies.

40. Vitturi R, Libertini A, Panozzo M and Mezzapelle G. KARYOTYPE ANALYSIS AND GENOME SIZE IN 3 MEDITERRANEAN SPECIES OF PERIWINKLES (PROSOBRANCHIA, MESOGASTROPODA). Malacologia. 1995;37 1:123-32.

41. Wang S, Zhang J, Jiao W, Li J, Xun X, Sun Y, et al. Scallop genome provides insights into evolution of bilaterian karyotype and development. Nat Ecol Evol. 2017;1 5:120. doi:10.1038/s41559-017-0120.

42. Servant N, Varoquaux N, Lajoie BR, Viara E, Chen CJ, Vert JP, et al. HiC-Pro: an optimized and flexible pipeline for Hi-C data processing. Genome Biology. 2015;16 doi:10.1186/s13059-015-0831-x.

43. Ruan J and Li H. Fast and accurate long-read assembly with wtdbg2. Nature Methods. 2020;17 2:155-+. doi:10.1038/s41592-019-0669-3.

44. Durand NC, Shamim MS, Machol I, Rao SSP, Huntley MH, Lander ES, et al. Juicer Provides a One-Click System for Analyzing Loop-Resolution Hi-C Experiments. Cell Systems. 2016;3 1:95-8. doi:10.1016/j.cels.2016.07.002.

45. Dudchenko O, Batra SS, Omer AD, Nyquist SK, Hoeger M, Durand NC, et al. De novo assembly of the Aedes aegypti genome using Hi-C yields chromosome-length scaffolds. Science. 2017;356 6333:92-5. doi:10.1126/science.aal3327.

46. Robinson JT, Turner D, Durand NC, Thorvaldsdottir H, Mesirov JP and Aiden EL.

Juicebox.js Provides a Cloud-Based Visualization System for Hi-C Data. Cell Systems. 2018;6 2:256-+. doi:10.1016/j.cels.2018.01.001.

47. Xu MY, Guo LD, Gu SQ, Wang O, Zhang R, Peters BA, et al. TGS-GapCloser: A fast and accurate gap closer for large genomes with low coverage of error-prone long reads. Gigascience. 2020;9 9 doi:10.1093/gigascience/giaa094.

48. Walker BJ, Abeel T, Shea T, Priest M, Abouelliel A, Sakthikumar S, et al. Pilon: An Integrated Tool for Comprehensive Microbial Variant Detection and Genome Assembly Improvement. Plos One. 2014;9 11 doi:10.1371/journal.pone.0112963.

49. Zhang X, Zhang S, Zhao Q, Ming R and Tang H. Assembly of allele-aware, chromosomal-scale autopolyploid genomes based on Hi-C data. Nature Plants. 2019;5 8:833-45. doi:10.1038/s41477-019-0487-8.

50. Waterhouse RM, Seppey M, Simão FA, Manni M, Ioannidis P, Klioutchnikov G, et al. BUSCO Applications from Quality Assessments to Gene Prediction and Phylogenomics. Molecular Biology and Evolution. 2017;35 3:543-8. doi:10.1093/molbev/msx319 %J Molecular Biology and Evolution.

51. Price AL, Jones NC and Pevzner PA. De novo identification of repeat families in large genomes. Bioinformatics. 2005;21 suppl_1:i351-i8. doi:10.1093/bioinformatics/bti1018 %J Bioinformatics.

52. Su W, Ou S, Hufford MB and Peterson T. A Tutorial of EDTA: Extensive De Novo TE Annotator. Methods in molecular biology (Clifton, NJ). 2021;2250:55-67. doi:10.1007/978-1-0716-1134-0_4.

53. Chen N. Using RepeatMasker to identify repetitive elements in genomic sequences.

Current protocols in bioinformatics. 2004;Chapter 4:Unit 4.10. doi:10.1002/0471250953.bi0410s05.

54. Grabherr MG, Haas BJ, Yassour M, Levin JZ, Thompson DA, Amit I, et al. Full-length transcriptome assembly from RNA-Seq data without a reference genome. Nature Biotechnology. 2011;29 7:644-U130. doi:10.1038/nbt.1883.

55. Li H. Minimap2: pairwise alignment for nucleotide sequences. Bioinformatics. 2018;34 18:3094-100. doi:10.1093/bioinformatics/bty191 %J Bioinformatics.

56. Haas BJ, Salzberg SL, Zhu W, Pertea M, Allen JE, Orvis J, et al. Automated eukaryotic gene structure annotation using EVidenceModeler and the program to assemble spliced alignments. Genome Biology. 2008;9 1 doi:10.1186/gb-2008-9-1-r7.

57. Shumate A, Wong B, Pertea G and Pertea M. Improved transcriptome assembly using a hybrid of long and short reads with StringTie. Plos Computational Biology. 2022;18 6 doi:10.1371/journal.pcbi.1009730.

58. Stanke M, Keller O, Gunduz I, Hayes A, Waack S and Morgenstern B. AUGUSTUS: ab initio prediction of alternative transcripts. Nucleic acids research. 2006;34 suppl_2:W435-W9. doi:10.1093/nar/gkl200 %J Nucleic Acids Research.

59. Hoff KJ, Lomsadze A, Borodovsky M and Stanke M. Whole-Genome Annotation with BRAKER. Methods in molecular biology (Clifton, NJ). 2019;1962:65-95. doi:10.1007/978-1-4939-9173-0_5.

60. Besemer J and Borodovsky M. GeneMark: web software for gene finding in prokaryotes, eukaryotes and viruses. Nucleic acids research. 2005;33 suppl_2:W451-W4. doi:10.1093/nar/gki487 %J Nucleic Acids Research.

61. Karin EL, Mirdita M and Soding J. MetaEuk-sensitive, high-throughput gene discovery, and annotation for large-scale eukaryotic metagenomics. Microbiome. 2020;8 1 doi:10.1186/s40168-020-00808-x.
62. Haas BJ, Salzberg SL, Zhu W, Pertea M, Allen JE, Orvis J, et al. Automated eukaryotic gene structure annotation using EVidenceModeler and the Program to Assemble Spliced Alignments. Genome Biology. 2008;9 1:R7. doi:10.1186/gb-2008-9-1-r7.
63. Consortium TU. UniProt: the Universal Protein Knowledgebase in 2023. Nucleic acids research. 2022;51 D1:D523-D31. doi:10.1093/nar/gkac1052 %J Nucleic Acids Research.
64. Mistry J, Chuguransky S, Williams L, Qureshi M, Salazar Gustavo A, Sonnhammer ELL, et al. Pfam: The protein families database in 2021. Nucleic acids research. 2020;49 D1:D412-D9. doi:10.1093/nar/gkaa913 %J Nucleic Acids Research.
65. Huerta-Cepas J, Szklarczyk D, Heller D, Hernández-Plaza A, Forslund SK, Cook H, et al. eggNOG 5.0: a hierarchical, functionally and phylogenetically annotated orthology resource based on 5090 organisms and 2502 viruses. Nucleic acids research. 2018;47 D1:D309-D14. doi:10.1093/nar/gky1085 %J Nucleic Acids Research.
66. Rawlings ND, Barrett AJ, Thomas PD, Huang X, Bateman A and Finn RD. The MEROPS database of proteolytic enzymes, their substrates and inhibitors in 2017 and a comparison with peptidases in the PANTHER database. Nucleic acids research. 2017;46 D1:D624-D32. doi:10.1093/nar/gkx1134 %J Nucleic Acids Research.
67. Drula E, Garron M-L, Dogan S, Lombard V, Henrissat B and Terrapon N. The carbohydrate-active enzyme database: functions and literature. Nucleic acids research.

2021;50 D1:D571-D7. doi:10.1093/nar/gkab1045 %J Nucleic Acids Research.

68. Jones P, Binns D, Chang H-Y, Fraser M, Li W, McAnulla C, et al. InterProScan 5: genome-scale protein function classification. Bioinformatics. 2014;30 9:1236-40. doi:10.1093/bioinformatics/btu031 %J Bioinformatics.

69. Buchfink B, Reuter K and Drost H-G. Sensitive protein alignments at tree-of-life scale using DIAMOND. Nature Methods. 2021;18 4:366-8. doi:10.1038/s41592-021-01101-x.

70. Emms DM and Kelly S. OrthoFinder: phylogenetic orthology inference for comparative genomics. Genome Biol. 2019;20 1:238. doi:10.1186/s13059-019-1832-y.

71. Katoh K and Standley DM. MAFFT multiple sequence alignment software version 7: improvements in performance and usability. Mol Biol Evol. 2013;30 4:772-80. doi:10.1093/molbev/mst010.

72. Castresana J. Selection of Conserved Blocks from Multiple Alignments for Their Use in Phylogenetic Analysis. Molecular Biology and Evolution. 2000;17 4:540-52. doi:10.1093/oxfordjournals.molbev.a026334 %J Molecular Biology and Evolution.

73. Nguyen L-T, Schmidt HA, von Haeseler A and Minh BQ. IQ-TREE: A Fast and Effective Stochastic Algorithm for Estimating Maximum-Likelihood Phylogenies. Molecular Biology and Evolution. 2014;32 1:268-74. doi:10.1093/molbev/msu300 %J Molecular Biology and Evolution.

74. Yang Z. PAML 4: Phylogenetic Analysis by Maximum Likelihood. Molecular Biology and Evolution. 2007;24 8:1586-91. doi:10.1093/molbev/msm088 %J Molecular Biology and Evolution.

75. Huang Z, Huang W, Liu X, Han Z, Liu G, Boamah GA, et al. Genomic insights into the adaptation and evolution of the nautilus, an ancient but evolving "living fossil". Mol Ecol Resour. 2022;22 1:15-27. doi:10.1111/1755-0998.13439.

76. Sun W and Gao L. Phylogeny and comparative genomic analysis of Pteriomorphia (Mollusca: Bivalvia) based on complete mitochondrial genomes. Marine Biology Research. 2017;13 3:255-68. doi:10.1080/17451000.2016.1257810.

77. Mendes FK, Vanderpool D, Fulton B and Hahn MW. CAFE 5 models variation in evolutionary rates among gene families. Bioinformatics. 2021;36 22-23:5516-8. doi:10.1093/bioinformatics/btaa1022.

78. Han MV, Thomas GWC, Lugo-Martinez J and Hahn MW. Estimating Gene Gain and Loss Rates in the Presence of Error in Genome Assembly and Annotation Using CAFE 3. Molecular Biology and Evolution. 2013;30 8:1987-97. doi:10.1093/molbev/mst100 %J Molecular Biology and Evolution.

79. Alexa A and Rahnenfuhrer J. topGO: Enrichment Analysis for Gene Ontology. R package version 2.54.0. 2023; doi:doi:10.18129/B9.bioc.topGO.

80. Wang Y, Tang H, Debarry JD, Tan X, Li J, Wang X, et al. MCScanX: a toolkit for detection and evolutionary analysis of gene synteny and collinearity. Nucleic acids research. 2012;40 7:e49. doi:10.1093/nar/gkr1293.

81. Rodrigues-Silva C, Flores-Nunes F, Vernal JI, Cargnin-Ferreira E and Bainy AC. Expression and immunohistochemical localization of the cytochrome P450 isoform 356A1 (CYP356A1) in oyster Crassostrea gigas. Aquatic toxicology (Amsterdam, Netherlands). 2015;159:267-75. doi:10.1016/j.aquatox.2014.12.021.

82. Anderson JF, Siller E and Barral JM. The sacsin repeating region (SRR): a novel Hsp90-related supra-domain associated with neurodegeneration. Journal of molecular biology. 2010;400 4:665-74. doi:10.1016/j.jmb.2010.05.023.

83. Stelzer G, Rosen N, Plaschkes I, Zimmerman S, Twik M, Fishilevich S, et al. The GeneCards Suite: From Gene Data Mining to Disease Genome Sequence Analyses. Curr Protoc Bioinformatics. 2016;54:1.30.1-1..3. doi:10.1002/cpbi.5.

84. Reid DG, Dyal P and Williams ST. A global molecular phylogeny of 147 periwinkle species (Gastropoda, Littorininae). Zoologica Scripta. 2012;41 2:125-36. doi:10.1111/j.1463-6409.2011.00505.x.

85. Reid DG. The Comparative Morphology, Phylogeny and Evolution of the Gastropod Family Littorinidae. Philosophical Transactions of the Royal Society B-Biological Sciences. 1989;324 1220:1-110. doi:DOI 10.1098/rstb.1989.0040.

86. Rodríguez-Trelles F, Tarrío R and Ayala FJ. A methodological bias toward overestimation of molecular evolutionary time scales. 2002;99 12:8112-5. doi:doi:10.1073/pnas.122231299.

87. Sokolova IM, Frederich M, Bagwe R, Lannig G and Sukhotin AA. Energy homeostasis as an integrative tool for assessing limits of environmental stress tolerance in aquatic invertebrates. Marine Environmental Research. 2012;79:1-15. doi:https://doi.org/10.1016/j.marenvres.2012.04.003.

88. Li L, Li A, Song K, Meng J, Guo X, Li S, et al. Divergence and plasticity shape adaptive potential of the Pacific oyster. Nature Ecology & Evolution. 2018;2 11:1751-60. doi:10.1038/s41559-018-0668-2.

Tables and Figures

Figure 1. Maximum likelihood phylogenetic tree constructed by MCMCTree with divergence time estimated among species. Numbers next to the nodes represented the estimated divergence time (million years ago [Ma]). Divergences used for the recalibration of time estimation were indicated with red squares. The credibility intervals with 95%HPD of the divergence time were shown in the parentheses.

Figure 2. Dot plot of genome macrosynteny between littorinids and *P. yessoensis* chromosomes. Each dot represents a common single-copy gene.

Figure 3. Chromosome macrosynteny of presumed ancient bilaterian ancestor (ALG), presumed common ancestor of *P. yessoensis* and the littorinids (MRCA of PY&Ls), *P. yessoensis* (PY) and littorinid (L) linkage groups. The karyotype evolution was indicated by arrows and the macrosynteny was indicated by grey bold lines.

Table 1 Summary of statistics for the *L. brevicula* and *L. sinensis* genome assembly

	L. brevicula	*L. sinensis*
Genome scaffold total:	3132	27
Genome contig total:	3702	935
Genome scaffold sequence total:	928.20Mb	822.61M
Genome contig sequence total:	927.93Mb (0.029%gap)	822.51Mb (0.011%gap)
Genome contig N50	3.43Mb	2.31Mb
Genome scaffold N50	48.134Mb	32.91Mb

Table 2 Classification of the repetitive elements in *L. brevicula* and *L. sinensis* genome assembly

	Count		Length(bp)		% of genome	
Type	*L. brevicula*	*L. sinensis*	*L. brevicula*	*L. sinensis*	*L. brevicula*	*L. sinensis*
DNA transposons	1,590,204	684,216	204,405,206	98,112,746	22.02	11.93
Retroelements	346,793	302,792	82,326,206	77,246,672	8.87	9.39
Other	1,049,612	1,286,201	91,614,177	119,303,375	9.87	14.5
Unknown	306,488	183,893	60,252,340	43,309,094	6.49	5.27
Total	3,293,097	2,457,102	438,527,929	337,971,887	47.25	41.09

Figure1

Click here to access/download;Figure;Figure_1.pdf

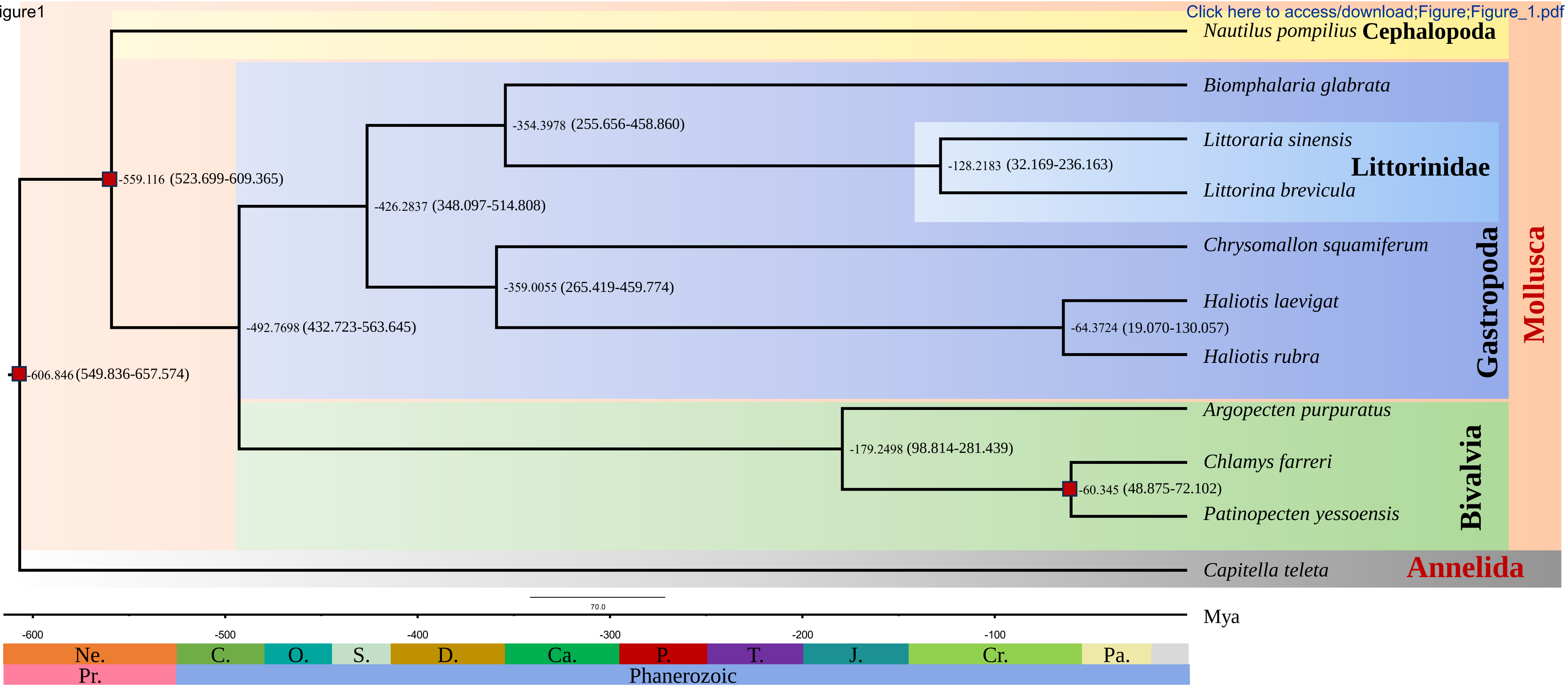

Figure 2

Click here to access/download;Figure;Figure_2.pdf

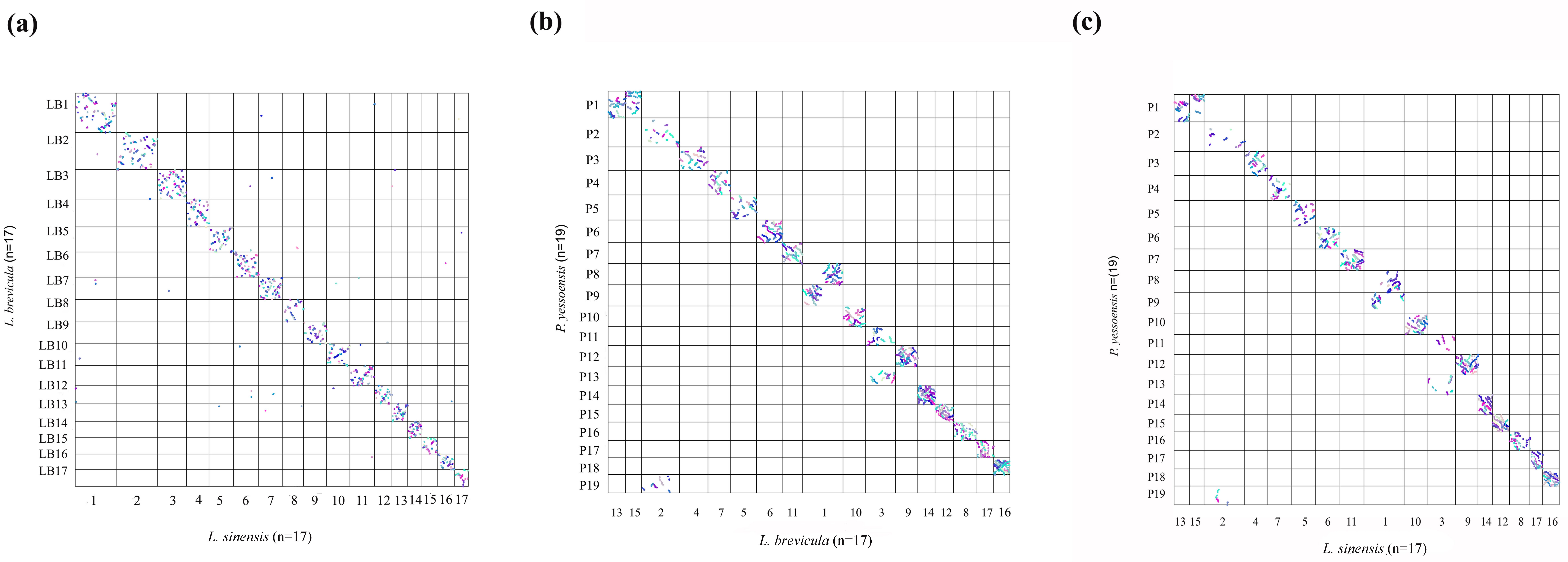

Figure3

Click here to access/download;Figure;Figure_3.pdf

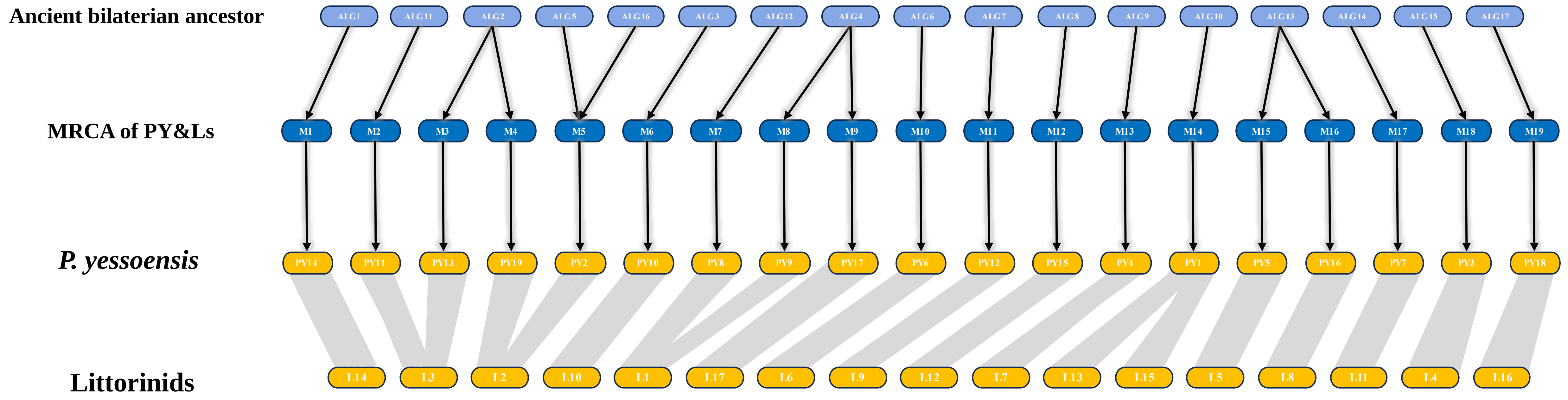

Click here to access/download
Supplementary Material
Supplementary_Material_GIGA-D-24-00090.docx

Cover letter

Click here to access/download;Personal Cover;Cover_Letter_GIGA-D-24-00090.docx

Dear Editor Hongfang Zhang,

Thank you for giving us an opportunity to revise our manuscript (GIGA-D-24-00090) to make it acceptable to GigaScience. The comments of the reviewers are all valuable, insightful, and very helpful for revising and improving the manuscript. We have thoroughly revised the manuscript following the recommendations. Please check our responses to the comments as reflected in the revised manuscript and response letter. We believe that we have addressed and accommodated the comments to the extent that is reasonable if not exhaustive. Accordingly, we hope that the manuscript can now be accepted for GigaScience.

Thank you again for your great editorial efforts.

Sincerely yours,

Jin-Xian Liu
