# Supplementary material for: Chromosome-level genome assemblies of two littorinid marine snails indicate genetic basis of intertidal adaptation and ancient karyotype evolved from bilaterian ancestors
Source: Gigascience. 2024 Sep 25;13:giae072. doi: 10.1093/gigascience/giae072 (PMC11423352; doi:10.1093/gigascience/giae072)
Supplement: giae072_Supplemental_File [file giae072_supplemental_file.docx]

Supplementary Materials for

Chromosome-level genome assemblies of two littorinid marine snails indicate genetic basis of intertidal adaptation and ancient karyotype evolved from bilaterian ancestors

Yan-Shu Wang^a,b,c,1^, Meng-Yu Li^a,b,c,1^, Yu-Long Li^a,b,1^, Yu-Qiang Li ^a,b,c^, Dong-Xiu Xue^a,b^, Jin-Xian Liu^a,b,2^

^a^ CAS Key Laboratory of Marine Ecology and Environmental Sciences, Institute of Oceanology, Chinese Academy of Sciences, Qingdao 266071, China; ^b^ Laboratory for Marine Ecology and Environmental Science, Qingdao Marine Science and Technology Center, Qingdao 266237, China; ^c^ University of Chinese Academy of Sciences, Beijing 100049, China.

^1^ These authors contributed equally to this work.

^2^ To whom correspondence should be addressed.

Correspondence to: jinxianliu@gmail.com

**This PDF file includes:**

Supplementary Tables S1 to S8

**Supplementary Table of Contents**

[Supplementary Table S1 Metazoan genome assemblies and gene models used in this study. 1](#_Toc174698940)

[Supplementary Table S2 Statistics of chromosomal level assembly of the two littorinid snails. 2](#_Toc174698941)

[Supplementary Table S3 Completeness assessment of the two littorinid snails by BUSCO. 3](#_Toc174698942)

[Supplementary Table S4 Genome size and repetitive elements of 46 mollusks. 4](#_Toc174698943)

[Supplementary Table S5 Statistics of predicted protein-coding genes in the genome assembly of *L. brevicula*. 6](#_Toc174698944)

[Supplementary Table S6 Statistics of predicted protein-coding genes in the genome assembly of *L. sinensis*. 7](#_Toc174698945)

[Supplementary Table S7 Statistics of gene families of 11 species in comparative genomic analysis. 8](#_Toc174698946)

[Supplementary Table S8 Potential candidate intertidal adaptation-related genes under positive selection. 9](#_Toc174698947)

# Supplementary Table S1 Metazoan genome assemblies and gene models used in this study.

| **Species** | **Assembly and gene models** |
| --- | --- |
| *Littorina brevicula* | This study |
| *Littoraria sinensis* | This study |
| *Argopecten purpuratus* | http://gigadb.org/dataset/100419 |
| *Biomphalaria glabrata* | http://download.mgbase.qnlm.ac/page/download/downloadall/ |
| *Chlamys farreri* | http://download.mgbase.qnlm.ac/page/download/downloadall/ |
| *Chrysomallon squamiferum* | http://download.mgbase.qnlm.ac/page/download/downloadall/ |
| *Haliotis laevigata* | https://abalonedb.org/genome-resources/data-downloads/ |
| *Halioti rubra* | http://doi.org/10.5281/zenodo.3320876 |
| *Nautilus pompilius* | https://doi.org/10.6084/m9.figsh are.14236208 |
| *Patinopecten yessoensis* | http://download.mgbase.qnlm.ac/page/download/downloadall/ |
| *Capitella teleta* | Ensembl Genomes 57 |
| *Lottia gigantea* | http://download.mgbase.qnlm.ac/home/work/molluscDB_download/Gastropoda/Lottia_gigantea/Lottia_gigantea.pep.fa.gz |
| *Haliotis discus* *hannai* | http://download.mgbase.qnlm.ac/home/work/molluscDB_download/Gastropoda/Haliotis_discus_hannai/Haliotis_discus_hannai.pep.fa.gz |
| *Elysia chlorotica* | http://download.mgbase.qnlm.ac/home/work/molluscDB_download/Gastropoda/Elysia_chlorotica/Elysia_chlorotica.pep.fa.gz |
| *Aplysia californica* | http://download.mgbase.qnlm.ac/home/work/molluscDB_download/Gastropoda/Aplysia_californica/Aplysia_californica.pep.fa.gz |
| *Octopus bimaculoides* | http://download.mgbase.qnlm.ac/home/work/molluscDB_download/Cephalopoda/Octopus_bimaculoides/Octopus_bimaculoides.pep.fa.gz |
| *Octopus minor* | http://download.mgbase.qnlm.ac/home/work/molluscDB_download/Cephalopoda/Octopus_minor/Octopus_minor.pep.fa.gz |
| *Pomacea canaliculata* | http://download.mgbase.qnlm.ac/home/work/molluscDB_download/Gastropoda/Pomacea_canaliculata/Pomacea_canaliculata.pep.fa.gz |

# Supplementary Table S2 Statistics of chromosomal level assembly of the two littorinid snails.

| **Chromosome ID** | **Length (bp)** | | **Percentage** | |
| --- | --- | --- | --- | --- |
|  | *L. brevicula* | *L. sinensis* | *L. brevicula* | *L. sinensis* |
| chr01 | 85,551,113 | 86,065,195 | 9.22% | 10.46% |
| chr02 | 80,887,714 | 86,092,370 | 8.71% | 10.47% |
| chr03 | 64,320,115 | 60,418,366 | 6.93% | 7.34% |
| chr04 | 59,908,826 | 47,132,172 | 6.45% | 5.73% |
| chr05 | 55,543,584 | 51,093,261 | 5.98% | 6.21% |
| chr06 | 54,401,117 | 52,088,366 | 5.86% | 6.33% |
| chr07 | 48,134,183 | 49,935,811 | 5.19% | 6.07% |
| chr08 | 50,381,270 | 44,061,869 | 5.43% | 5.36% |
| chr09 | 47,250,897 | 48,007,041 | 5.09% | 5.84% |
| chr10 | 47,386,676 | 49,032,721 | 5.11% | 5.96% |
| chr11 | 43,280,250 | 51,198,554 | 4.66% | 6.22% |
| chr12 | 39,983,170 | 36,337,722 | 4.31% | 4.42% |
| chr13 | 37,184,335 | 33,492,581 | 4.01% | 4.07% |
| chr14 | 35,937,534 | 30,920,885 | 3.87% | 3.76% |
| chr15 | 35,236,845 | 32,905,188 | 3.80% | 4.00% |
| chr16 | 34,464,715 | 35,012,720 | 3.71% | 4.26% |
| chr17 | 36,063,020 | 28,644,308 | 3.89% | 3.48% |
| Unplaced | 72,289,073 | 165,549 | 7.79% | 0.02% |

# Supplementary Table S3 Completeness assessment of the two littorinid snails by BUSCO.

| **BUSCO feature** | **Statistic** | |
| --- | --- | --- |
|  | *L. brevicula* | *L. sinensis* |
| Complete BUSCOs(C) | 93.1%(888) | 93.8%(895) |
| Complete and single-copy BUSCOs (S) | 91.9%(877) | 90.6%(864) |
| Complete and duplicated BUSCOs(D) | 1.2%(11) | 3.2%(31) |
| Fragmented BUSCOs (F) | 3.7%(35) | 3.4%(32) |
| Missing BUSCOs (M) | 3.2%(31) | 2.8%(27) |
| total lineage BUSCOs | 954 | 954 |

# Supplementary Table S4 Genome size and repetitive elements of 46 mollusks.

| **Species** | **Group** | **Genome size (Mb)** | **Contig N50 (Kb)** | **Repetitive elements (%)** |
| --- | --- | --- | --- | --- |
| *Littorina brevicula* | gastropoda | 928 | 3.4 | 47.25 |
| *Littoraria sinensis* | gastropoda | 823 | 2.3 | 41.09 |
| *Pomacea canaliculata* | gastropoda | 440 | 1.1 | 20.53 |
| *Lottia gigantea* | gastropoda | 360 | 0.096 | 21 |
| *Pomacea maculata* | gastropoda | 432 | 0.091 | 21.25 |
| *Chrysomallon squamiferum* | gastropoda | 444 | 1.9 | 25.2 |
| *Lanistes nyassanus* | gastropoda | 510 | 0.034 | 28.87 |
| *Aplysia californica* | gastropoda | 927 | 0.01 | 30 |
| *Marisa cornuarietis* | gastropoda | 536 | 4.4 | 30.82 |
| *Elysia chlorotica* | gastropoda | 557 | 0.029 | 32.6 |
| *Conus betulinus* | gastropoda | 3512 | 0.17 | 38.56 |
| *Radix auricularia* | gastropoda | 910 | 0.58 | 41.66 |
| *Biomphalaria glabrata* | gastropoda | 916 | 0.073 | 44.8 |
| *Lautoconus ventricosus* | gastropoda | 3676 | 0.19 | 53.36 |
| *Crassostrea ariakensis* | gastropoda | 614 | 6.97 | 54.14 |
| *Achatina immaculata* | gastropoda | 1653 | 3.8 | 54.5 |
| *Candidula unifasciata* | gastropoda | 1321 | 0.25 | 61.1 |
| *Bathyacmaea lactea* | gastropoda | 754 | 1.57 | 61.4 |
| *Achatina fulica* | gastropoda | 1976 | 0.66 | 71 |
| *Arion vulgaris* | gastropoda | 1577 | 8.6 | 75 |
| *Cepaea nemoralis* | gastropoda | 3584 | 0.33 | 76.7 |
| *Patinopecten yessoensis* | bivalve | 988 | 0.038 | 27.83 |
| *Dreissena rostriformis* | bivalve | 1270 | 0.13 | 31.9 |
| *Chlamys farreri* | bivalve | 780 | 0.022 | 32.1 |
| *Argopecten purpuratus* | bivalve | 725 | 0.08 | 33.74 |
| *Crassostrea gigas* | bivalve | 648 | 1.8 | 36 |
| *Sinonovacula constricta* | bivalve | 1332 | 0.68 | 39.79 |
| *Cyclina sinensis* | bivalve | 903 | 2.6 | 43.14 |
| *Scapharca kagoshimensis* | bivalve | 1114 | 2 | 44.97 |
| *Crassostrea hongkongensis* | bivalve | 610 | 2.6 | 45 |
| *Saccostrea glomerata* | bivalve | 788 | 0.04 | 45.03 |
| *Scapharca broughtonii* | bivalve | 885 | 1.8 | 46.1 |
| *Pinctada fucata* | bivalve | 1024 | 0.021 | 47.09 |
| *Mytilus coruscus* | bivalve | 1946 | 0.09 | 47.4 |
| *Bathymodiolus platifrons* | bivalve | 1660 | 0.013 | 49.08 |
| *Mercenaria mercenaria* | bivalve | 1833 | 1.77 | 49.11 |
| *Tegillarca granosa* | bivalve | 813 | 0.6 | 53.56 |
| *Modiolus philippinarum* | bivalve | 2630 | 0.02 | 61.08 |
| *Nautilus pompilius* | cephalopoda | 785 | 2.5 | 30 |
| *Octopus sinensis* | cephalopoda | 2785 | 0.49 | 42.26 |
| *Octopus bimaculoides* | cephalopoda | 2867 | 0.0054 | 43 |
| *Octopus minor* | cephalopoda | 5212 | 0.2 | 44.43 |

# Supplementary Table S5 Statistics of predicted protein-coding genes in the genome assembly of *L. brevicula*.

|  | Gene number | Gene | Exon | Exon | CDS | CDS | Intron | Intron |
| --- | --- | --- | --- | --- | --- | --- | --- | --- |
|  |  | length(bq) | number | length(bp) | number | length(bp) | number | length(bp) |
| Augustus | 34674 | 295034110 | 180736 | 42008685 | 180736 | 42008685 | 146062 | 284554959 |
| GeneMark | 63045 | 439739035 | 351385 | 72352020 | 351385 | 72352020 | 288340 | 420740249 |
| GlimmerHMM | 102779 | 815976119 | 442892 | 84048912 | 442892 | 84048912 | 340113 | 731927207 |
| snap | 106714 | 433479633 | 235562 | 48522306 | 235562 | 48522306 | 128848 | 408900899 |
| pasa | 16814 | 147049896 | 75537 | 21558531 | 69236 | 13599948 | 58723 | 137473020 |
| BRAKER | 62069 | 412536748 | 338680 | 70682528 | 338582 | 70657862 | 276611 | 427400900 |
| StringTie | 22821 | 352778998 | 298782 | 82953013 | 242022 | 39915129 | 275961 | 936869119 |
| Metaeuk | 28140 | 228895825 | 122085 | 30813543 | 122085 | 30329379 | 93945 | 214937370 |

# Supplementary Table S6 Statistics of predicted protein-coding genes in the genome assembly of *L. sinensis*.

|  | Gene number | Gene | Exon | Exon | CDS | CDS | Intron | Intron |
| --- | --- | --- | --- | --- | --- | --- | --- | --- |
|  |  | length(bq) | number | length(bp) | number | length(bp) | number | length(bp) |
| Augustus | 35447 | 259738593 | 170999 | 37838537 | 170999 | 37838537 | 135552 | 250337236 |
| GeneMark | 45271 | 460995069 | 334280 | 62885658 | 334280 | 62885658 | 289009 | 449002676 |
| GlimmerHMM | 96017 | 732805423 | 433889 | 80809013 | 433889 | 80809013 | 337872 | 651996410 |
| snap | 89382 | 103685469 | 615508 | 99868582 | 615508 | 99868582 | 526126 | 102498509 |
| pasa | 16432 | 147973099 | 77708 | 24649405 | 73323 | 15251451 | 61276 | 136974809 |
| BRAKER | 16412 | 223532923 | 197422 | 35043360 | 197422 | 35043360 | 181010 | 390634430 |
| StringTie | 23891 | 377313462 | 372490 | 11454963 | 329559 | 58392819 | 348599 | 127921109 |
| Metaeuk | 19668 | 127670887 | 64511 | 18668553 | 64511 | 18562146 | 44843 | 117829889 |

# Supplementary Table S7 Statistics of gene families of 11 species in comparative genomic analysis.

| **Species** | **Genes number** | **Clustered genes** | **Unclustered genes** | **Family number** | **Unique families** | **Average genes per family** |
| --- | --- | --- | --- | --- | --- | --- |
| *Littorina brevicula* | 29,335 | 26,804 | 2,531 | 15,013 | 522 | 1.79 |
| *Littoraria sinensis* | 25,386 | 23,707 | 1,679 | 14,876 | 344 | 1.59 |
| *Argopecten purpuratus* | 26,256 | 23,176 | 3,080 | 14,823 | 204 | 1.56 |
| *Biomphalaria glabrata* | 36,428 | 32,460 | 3,968 | 12,602 | 1,716 | 2.58 |
| *Chlamys farreri* | 27,977 | 23,994 | 3,983 | 15,330 | 229 | 1.57 |
| *Chrysomallon squamiferum* | 16,917 | 15,156 | 1,761 | 10,229 | 305 | 1.48 |
| *Haliotis laevigata* | 16,269 | 15,343 | 926 | 11,038 | 53 | 1.39 |
| *Halioti rubra* | 42,940 | 40,245 | 2,695 | 14,348 | 1,520 | 2.80 |
| *Nautilus pompilius* | 15,096 | 12,645 | 2,451 | 9,910 | 93 | 1.28 |
| *Patinopecten yessoensis* | 28,341 | 26,135 | 2,206 | 15,826 | 360 | 1.65 |
| *Capitella teleta* | 32,175 | 27,721 | 4,454 | 11,817 | 1,504 | 2.35 |

# Supplementary Table S8 Potential candidate intertidal adaptation-related genes under positive selection.

| **Orthogroup_ID** | **Name** | **Product** |
| --- | --- | --- |
| OG0005689 | DPP3 | Dipeptidyl peptidase III |
| OG0005730 | PPP4R4 | Serine/threonine-protein phosphatase 4 regulatory subunit 4 |
| OG0005802 | CDYL2_1 | Enoyl-CoA hydratase 1 |
| OG0005869 | ERLEC1 | Endoplasmic reticulum lectin 1 |
| OG0005906 | ATF4 | BZIP domain-containing protein |
| OG0005925 | TRAFD1 | TRAF-type domain-containing protein |
| OG0005991 | TPX2 | GPI ethanolamine phosphate transferase 1 |
| OG0006072 | GLIS2 | Zinc finger protein GLIS2 |
| OG0006087 | CHD7 | DNA helicase |
| OG0006119 | PPIL4 | Peptidyl-prolyl cis-trans isomerase |
| OG0006127 | TTC27 | TPR_REGION domain-containing protein |
| OG0006141 | RBBP6 | E3 ubiquitin-protein ligase RBBP6 |
| OG0006146 | IRAK1 | Interleukin-1 receptor-associated kinase 1-like protein |
| OG0006214 | FBF1 | Fas-binding factor 1 |
| OG0006224 | UTP11L | putative U3 small nucleolar RNA-associated protein 11 |
| OG0006274 | PPRC1 | RRM domain-containing protein |
| OG0006388 | AK7_2 | Nucleoside-diphosphate kinase |
| OG0006400 | ZCWPW1 | Zinc finger CW-type PWWP domain protein 1 |
| OG0006530 | CAMKK2 | Protein kinase domain-containing protein |
| OG0006535 | CASZ1 | DNA helicase |
| OG0006631 | IGF2R | Cation-independent mannose-6-phosphate receptor-like protein |
| OG0006641 | MCM3 | DNA replication licensing factor MCM3 |
| OG0006644 | lat-2_2 | Latrophilin Cirl |
| OG0006745 | USP54 | USP domain-containing protein |
| OG0006759 | UFSP2 | Ufm1-specific protease 2 |
| OG0006840 | KDM3B | JmjC domain-containing protein |
| OG0006939 | ABCE1_1 | ATP-binding cassette sub-family E member 1 |
| OG0006969 | ATG4A | Cysteine protease |
| OG0007043 | SNRNP48 | U11/U12 small nuclear ribonucleoprotein 48 kDa protein |
| OG0007045 | FBXW8 | F-box domain-containing protein |
| OG0007087 | UBA1 | Ubiquitin-activating enzyme E1 |
| OG0007098 | RBMX2 | RRM domain-containing protein |
| OG0007149 | Surf2 | Surfeit locus protein 2 |
| OG0007168 | RAI14 | ANK_REP_REGION domain-containing protein |
| OG0007203 | RPN1 | Dolichyl-diphosphooligosaccharide--protein glycosyltransferase subunit 1 |
| OG0007277 | RPA1_3 | Replication protein A subunit |
| OG0007379 | HSPA12A | Beta/gamma crystallin domain-containing protein 1 |
| OG0007538 | FOXRED1 | FAD-dependent oxidoreductase domain-containing protein 1 |
| OG0007593 | SMARCA4 | ATP-dependent helicase brm |
| OG0007824 | TNFRSF9 | TNFR-Cys domain-containing protein |
| OG0007906 | CELSR2_2 | Cadherin EGF LAG seven-pass G-type receptor 2 |
| OG0008000 | CWC27 | PPIase cyclophilin-type domain-containing protein |
| OG0008014 | ENO4 | 2-phospho-D-glycerate hydro-lyase |
| OG0008040 | Hr4 | Nuclear receptor domain-containing protein |
| OG0008063 | G6PD | Glucose-6-phosphate 1-dehydrogenase |
| OG0008081 | COX15 | Cytochrome c oxidase assembly protein COX15 homolog |
| OG0008113 | RILPL1 | RILP-like protein 1 |
| OG0008127 | MKI67 | FHA domain-containing protein |
| OG0008333 | ZNHIT2 | Zinc finger HIT domain-containing protein 2 |
| OG0008394 | ech-8 | Enoyl-CoA hydratase |
| OG0008425 | PGM2 | Phosphoglucomutase-2 |
| OG0008535 | LAMP1_2 | Lysosome-associated membrane glycoprotein 1 |
| OG0008565 | P4HB | Protein disulfide-isomerase |
| OG0008617 | FBXO7 | F-box domain-containing protein |
| OG0008714 | AHSA1 | Aha1_N domain-containing protein |
| OG0008851 | MTHFD1 | C-1-tetrahydrofolate synthase, cytoplasmic |
| OG0008902 | WDR6 | WD repeat-containing protein 6 |
| OG0009032 | MDH1B | Ofus.G11310 protein |
| OG0009072 | PUF60 | Poly(U)-binding-splicing factor PUF60 |
| OG0009095 | MRPS27 | Mitochondrial 28S ribosomal protein S27 |
| OG0009107 | DNAJC2 | DnaJ homolog subfamily C member 2 |
| OG0009121 | HMGB2 | High mobility group protein B2 |
| OG0009141 | SIK2 | Non-specific serine/threonine protein kinase |
| OG0009160 | PSMB5 | Proteasome subunit beta |
| OG0009168 | DEPDC5 | DEPDC5_CTD domain-containing protein |
| OG0009194 | TOP2B | DNA topoisomerase 2 |
| OG0009206 | REV1_1 | DNA repair protein REV1 |
| OG0009230 | NSMCE2 | Non-structural maintenance of chromosomes element 2 homolog |
| OG0009264 | CHD1 | DNA helicase |
| OG0009340 | MPHOSPH10 | U3 small nucleolar ribonucleoprotein MPP10 |
| OG0009428 | SMC2_1 | SMC hinge domain-containing protein |
| OG0009494 | USP5 | Ubiquitin carboxyl-terminal hydrolase |
| OG0009568 | SLU7 | Pre-mRNA-splicing factor SLU7 |
| OG0009782 | AHCTF1 | Protein ELYS |
|  |  |  |
| OG0009860 | LONP1 | Lon protease homolog |
| OG0009913 | ENTPD3 | Ectonucleoside triphosphate diphosphohydrolase 1 |
| OG0010064 | TXNDC11 | Thioredoxin domain-containing protein |
| OG0010068 | GBA2 | Non-lysosomal glucosylceramidase |
| OG0010078 | POLD1 | DNA polymerase |
| OG0010104 | STOML2 | PHB domain-containing protein |
| OG0010216 | GPN1 | GPN-loop GTPase |
| OG0010354 | PGM1 | Phosphoglucomutase (alpha-D-glucose-1,6-bisphosphate-dependent) |
| OG0010361 | TOP1 | DNA topoisomerase 1 |
| OG0010394 | BRAT1 | DUF4704 domain-containing protein |
| OG0010442 | ANKZF1 | C2H2-type domain-containing protein |

Product: Proteins generated from these positively selected genes.
