# Supplementary material for: Chromosome-level genome assemblies of two littorinid marine snails indicate genetic basis of intertidal adaptation and ancient karyotype evolved from bilaterian ancestors
Source: Gigascience. 2024 Sep 25;13:giae072. doi: 10.1093/gigascience/giae072 (PMC11423352; doi:10.1093/gigascience/giae072)
Supplement: giae072_Response_to_Reviewer_Comments_Original_Submission [file giae072_response_to_reviewer_comments_original_submission.pdf]

Author's Response To Reviewer Comments

Dear Editor Hongfang Zhang,
Thank you for giving us an opportunity to revise our manuscript (GIGA-D-24-00090) to make it acceptable to GigaScience. The comments of the reviewers are all valuable, insightful, and very helpful for revising and improving the manuscript. We have thoroughly revised the manuscript following the recommendations. Please check our responses to the comments as reflected in the revised manuscript and response letter. Our response to the reviewers’ comments are listed below.We believe that we have addressed and accommodated the comments to the extent that is reasonable if not exhaustive. Accordingly, we hope that the manuscript can now be accepted for GigaScience.
Thank you again for your great editorial efforts.
Sincerely yours,

Jin-Xian Liu

Response to Reviewer 1 Comments

Response: We would like to thank you for your careful reading, helpful comments, and constructive suggestions, which have significantly improved the presentation of our manuscript.
We have carefully considered all comments from the reviewers and revised our manuscript accordingly. The manuscript has also been double-checked, and the typos and grammar errors we found have been corrected. We have simplified the discussion about comparative genomic analysis and revised Fig. 3 to make it clearer to understand. In the following section, we summarize our responses to each comment. We believe that our responses have well addressed all the concerns of the reviewers. We hope our revised manuscript can be accepted for publication.

P4, bottom - a helpful citation here would be Johannesson et al. 2024 Trends in Genetics
Response: We thank the reviewer for providing us with a recent review article supporting the importance that littorinid snails have in speciation and adaption. We have added the citation of the corresponding article to our manuscript at P4 line 6.
P5, top - citation 17 does not mention bacteria or viruses and so seems inappropriate here.
Response: Thank you for pointing out this problem in the manuscript. We corrected the citation at P4 line 12-13.
P5, mid, and P6 - note that a high-quality genome with annotation is now available for Littorina saxatilis: GBE https://doi.org/10.1093/gbe/evae076
Response: Thank you for pointing out the issue. Considering the publication of the L. saxatilis genome, we made some revisions in P4 line 20 to P5 line 2.
P6, bottom - it is unsafe to claim that gastropods are unchanged over these long time-periods because we know little beyond the form of the shell for early taxa.
Response: Thank you for pointing out this problem. We have removed this sentence in the revised manuscript.
P8, mid - the papers by Simakov and coworkers show that multiple taxa have karyotypes that can be related to the bilaterian ALGs (indeed, this is how the ALGs were identified). Therefore, the gross similarity of the littorinid LGs with the ALGs does not make littorinids particularly strong models for the study of early bilaterian evolution.
Response: Thank you for your suggestion. Macrosynteny analyses showed the karyotype of these two littorinids and bilaterian ancestor might be highly conserved. However, we didn’t make a comparation among the correspondence between different taxa and the presumed bilaterian ALGs, so it is improper to consider littorinids as a particularly good model for studying the evolution of early bilaterian ancestor. We have removed this sentence in the revised manuscript.
P10, bottom - I would have expected a duplicate removal step in this pipeline although I note that duplicated BUSCOs are not common
Response: Thank you for pointing out this issue. After the assembly of two littorinid genomes using Wtdbg2 and NextDenovo, we assessed the genome quality using BUSCO. Just as you mentioned, we found that

duplicated BUSCOs were quite scarce in these two genomes (1.2% in L. brevicula and 3.2% in L. sinensis). Therefore we thought that the duplicates might make little difference to the completeness and accuracy for the genome assembly and didn’t remove them.
P14, top - generic names should be given here in full. Haliotis is the only gastropod in this background set and it is in a different subclass from Littorinidae (common ancestor more than 400Mya). Therefore, there is the opportunity for many differences to have evolved under positive selection that are unrelated to the intertidal environment. The test for positive selection should be adjusted for multiple comparisons since many genes were investigated without a priori predictions.
Response: Thank you for the suggestion. We have revised the generic names into full name. Considering the divergence time of the littorinid snails and the closest related lineage was over 400Mya, the genes that was identified to be positively selected may be related with many differences like life-history instead of just intertidal adaptation. So in the revised manuscript, FDR correction was performed on the results ($p < 0.01$) and the positively selected genes were functionally annotated, based on which only 85 genes were considered to be potential candidate genes for intertidal adaptation. The discussion was only about this potential candidate gene set on page 16 line 19 – page 17 line 3.

Table 1 - the meaning of 'Main genome' should be explained in the legend
Response: Thank you for pointing out this issue. We directly used the headers from the results generated from the script carelessly. This table shows statistics for the chromosome-level assembly of the two littorinids. We find that the word “main” here is improper and meaningless, so we made a correction and delete it.
Fig. 3 - this would be much more legible if LGs were re-ordered, rather than following their (arbitrary) numbering.
Response: Thank you for your suggestion. We have re-ordered the bars representing different LGs and made the figure more clear and easier to understand (Figure 3).
P16 - either a mutation rate must have been used to infer times, or a calibration point or points, but I did not find the rate or source of calibration in the MS. The split time given here (128My) is much older than in Reid et al 2012 (90-90My).
Response: We thank the reviewer for pointing out this issue. Here in our work, 3 calibration points were used to estimate the divergence time for nodes in the phylogenetic tree: the divergence time between Capitella teleta and molluscan species (534.3-654.0 Mya) from citation 75; the divergence time between Nautilus pompilius and Bivalvia and Gastropoda species (527.6-619.1 Mya) from citation 75; the divergence time between Chlamys farreri and Patinopecten yessoensis (46.1-71.7 Mya) from the Timetree database (http://www.timet ree.org/) and citation 76. This part is rewritten in the “Gene family, phylogenetic analysis, and divergence time estimation” section in “Material and Methods” (page 10 line 6-10) in the revised manuscript and three calibration points were marked using red squares in Figure 1. The differences between our estimated divergence time and previous studies were discussed in the paragraph of “Discussion” (page 15 line 17 – page 16 line 9).
P17, bottom - 'collinearity' is not a good word here, because of the extensive within-chromosome rearrangements
Response: Thank you for pointing out this problem. Considering our macrosynteny analyses didn’t show the rearrangements within each chromosome, ‘collinearity’ seems to be improper here. We have changed the word ‘collinearity’ into ‘correspondence’ to describe the synteny between chromosomes, as Wang et al. (2017) used before in citation 41.
Figure 3 - a better layout could be found. The current layout risks implying that evolution proceeded from the ALG arrangement, to the PYE arrangement and then to the littorinid arrangement. In reality, there was a step from the ALG to a common molluscan ancestral state and then, independently, to the PYE and littorinid tips. Some features of the ancestral molluscan state can be inferred and so a layout that showed this node would be helpful.
Response: Thank you for your suggestion. We have added a new layout representing the common LGs that the scallop and littorinid snails may share in the most recent common ancestor. The evolution is now indicated by arrows while macrosynteny indicating by bold lines (Figure 3).
P21, bottom - I don't think that synteny between L. brevicula and L. sinensis is shown at any point (despite mention here).
Response: Thank you for pointing out this. Macrosynteny analysis has also been performed between L. brevicula and L. sinensis, of which the result was shown in Figure 2a and detailly described in section

“Evolution of littorinid chromosomes from the ancient bilaterian ancestor” in “Results” (page 14 line 17-19).
P22, top - care is needed here - it is only the gross karyotype that is shown to be 'slow-evolving' and one should not imply slow evolution of any other aspect of gastropod or littorinid biology.
Response: Thank you for pointing out this. On one hand, our macrosynteny analyses didn’t include intra-chromosome rearrangements, on the other hand, it was improper to define the speed of evolution as ‘slow’ or ‘rapid’ without comparation. We have removed this sentence in the revised manuscript.
P22-23 - the Conclusions section largely repeats aspects of the Discussion and is probably not needed.
Response: Thank you for pointing out this. We have deleted this section in the revised manuscript.

Response to Reviewer 2 Comments

Response: We would like to thank you for your careful reading, helpful comments, and constructive suggestions, which have significantly improved the presentation of our manuscript.
We have carefully considered all comments from the reviewers and revised our manuscript accordingly. The manuscript has also been double-checked, and the typos and grammar errors we found have been corrected. The Phred quality scores for ONT long reads were calculated resulting in Q20=74.96% and Q30=74.95%. In the following section, we summarize our responses to each comment. We believe that our responses have well addressed all the concerns of the reviewers. We hope our revised manuscript can be accepted for publication.
- title: I think "reveal" is too strong as the authors have only detected candidate genes and no functional assay has been performed
Response: Thank you for pointing out this. We have changed the word ‘reveal’ into ‘indicate’ since there was no gene functional assay and solid evidence.
- abstract, line 1: delete "evolutionary" before "adaptation"
Response: Thank you for your suggestion. We have removed the word “evolutionary” before “adaptation”.
- abstract, line 1: understanding adaptation to environments and the early evolution of bilaterians are two important aims of evolutionary biology but very different and I am not sure they can be combined in the same sentence.
Response: Thank you for your suggestion. We have changed the beginning of the abstract into ‘Living in the intertidal environment with a common karyotype of 17 chromosomes (which is the same with the presumed number of the ancient linkage groups (ALGs) of bilaterian ancestor), littorinid snails are excellent models for understanding the adaptation to harsh fluctuating environments and early evolution of bilaterians.’ in the revised manuscript page 2 line 2-5.
- abstract, line 8: I find no support in the study to assert that the mentioned gene families "played key roles in adaptation to multiple biotic and abiotic stresses in intertidal environments".
Response: Thank you for pointing out this issue. It is bold to say that these gene families "played key roles in adaptation to multiple biotic and abiotic stresses in intertidal environments" without functional assay. We have changed this sentence into “Comparative genomic analyses identified 92 expanded gene families and 85 potential candidate positively selected genes for intertidal adaptation in the littorinid lineage, which are functionally enriched in stimulus responses, innate immunity and apoptosis processes regulating and may be involved in cellular homeostasis maintenance in stressful intertidal environments.” in page 2 line 8-12.
- Page 3, line 7: "processes" instead of "process"
Response: Thank you for pointing it out. We have changed the word “process” into “processes”.
- Page 3, line 7: "evolve" instead of "acquire"
Response: Thank you for pointing it out. We have changed the word “acquire” into “evolve”.
- Page 3, line12: please sustain better with references that the genetic basis of adaptation to fluctuating environments remains mostly unknown.
Response: Thank you for pointing out this issue. We have rewritten this sentence into “To better understand how they survive in and adapt to fluctuating environments, it is crucial to elucidate the genetic mechanistic basis.” and added two references (citation 4 and 5) in page 3 line 8-10.
- Page 3, line 2 from the end: rhythm? Instead of "rhyme"
Response: Thank you for pointing it out. We have changed the word “rhyme” into “rhythm”.
- Page 4: This is a data note and in this regard, I find the introduction from this page on rather long and verbose. In page 4, I suggest deleting many sentences and focusing directly on the Littorinidae (line 13).
Response: Thank you for pointing out this issue. The manuscript was firstly written as a research article so

the introduction is too long for a data note. We have rewritten the beginning of this paragraph and made some simplification about background to focus directly on the littorinid snails at the end of page 3.
- Page 5, line 2: rhythm? Instead of "rhyme"
Response: Thank you for pointing it out. We have changed the word "rhyme" into "rhythm".
- Page 5, line 6: are really periwinkles the best model to understand adaptation to climate change? Please support this asseveration.
Response: Thank you for pointing out this issue. Based on previous reviews and researches, we are not sure if periwinkles are the "best" model for understanding the adaptation to climate change, but considering the steep environmental gradient of intertidal shores, periwinkles are ideal models for elucidating possible responses of organisms to climate, especially temperature changes. Two references were added to support this asseveration in the revised manuscript (page 4 line 9 to 13).
- Page 5, line 15 to Page 6, line 7: these sentences can be deleted to maintain the focus of the introduction. Start the paragraph with "Yet, no …"
Response: Thank you for pointing out this issue. We have deleted these corresponding sentences and rewritten the paragraph about littorinid genomes since the genome of L. saxatilis was reported in February 2024 (Page 4 line 19 to page 5 line 3).
- Page 6, line 4: from the end: I would say that is a key question for evolutionary bioloy not only for the Evo-Devo field.
Response: Thank you for this suggestion. We have rewritten this sentence into "Understanding how the enigmatic urbilateria, the last common ancestor of all bilaterians, was constructed is one of the key questions for evolutionary biology." in page 5 line 4-5.
- Page 6, last line: "almost unchanged from their ancestors". This asseveration is simply not true. After hundred million years of evolution, extant gastropods have diverged considerably from their ancestors. The authors may be referring to the external resemblance of shells in extant and fossil snails. Please delete this sentence.
Response: Thank you for pointing out this issue. We have removed this sentence in the revised manuscript.
- Page 7, line 2: The gastropods are as good as e.g., bivalves, cephalopods, caudofoveates to represent mollusks in comparative studies on the bilaterian ancestor, and mollusks are as good as any other phylum for this purpose. Please delete or rewrite this sentence.
Response: Thank you for your suggestion. We have deleted this sentence in the revised manuscript.
- Page 7, lines 5-8: These sentences can be deleted to alleviate the introduction.
Response: Thank you for your suggestion. We have deleted these sentences in the revised manuscript.
- Page 8, line 1: add "including detection of expanding/ contracting gene families and positively selected genes" before "were performed", and the delete sentences from line 2 to 12 (end of introduction).
Response: Thank you for your suggestion. We have rewritten the sentence into "To investigate genetic mechanisms associated with adaptation to intertidal harsh and fluctuating environments, comparative genomic analyses including detection of expanding/contracting gene families and positively selected genes were performed. Macrosynteny analysis were also conducted to uncover karyotype evolution from the bilaterian ancestor to mollusks." in page 6 line 2-6 and made the deletion in the revised manuscript.
- Page 8, line 3 from the end: Please go straight to what worked and delete this whole paragraph (that continues in page 9).
Response: Thank you for your suggestion. We have removed these corresponding sentences in the first paragraph of "Sampling, genomic DNA extraction and sequencing" and started directly with what worked.
- Page 9, line 12: delete "genomic" before "DNA" and add after "from a single individual". Please specify the source tissue (foot?)
Response: Thank you for your suggestion. We have rewritten this sentence into "For genomic sequencing of L. brevicula, genomic DNA was extracted using the E.Z.N.A Mollusc DNA Kit from foot muscle tissue of a single individual." in page 6 line 12-13.
- Page 9, line 13: delete "for genome assembly and correction". Delete "of a Qingdao L. brevicula individual"
Response: Thank you for your suggestion. We have made the deletion and rewritten this sentence into "Genomic DNA was sheared by a g-TUBE device (Catalog No. 520079, Covaris, MA) and then repaired and purified for further PacBio CLR library preparation according to the manufacturer's protocol (Pacific Biosciences, CA)." in page 6 line 13-16.
- Page 9, line 15: Please specify that you used the CLR and not the HiFi technology of PacBio.
Response: Thank you for your suggestion. We have rewritten this sentence into "Genomic DNA was

sheared by a g-TUBE device (Catalog No. 520079, Covaris, MA) and then repaired and purified for further PacBio CLR library preparation according to the manufacturer's protocol (Pacific Biosciences, CA)." in page 6 line 13-16.
- Page 9, last line and page 10 first paragraph: I suggest first explaining the ONT procedure and then explaining that in order to polish the assemblies, an Illumina sequencing was performed on the DNBSEQ-T7 system. Please specify the source tissue (foot?)
Response: Thank you for your suggestion. We have rewritten this paragraph into "For L. sinensis, genomic DNA from foot muscle tissue of a single snail was extracted using a Genomic-tip 100G (QIANGEN) kit and sheared and size-selected with the aforementioned procedure for the SMRT library. ONT libraries were constructed with these selected fragments using the Ligation Sequencing 1D Kit (Oxford Nanopore, Oxford, UK, p/n SQK-LSK109) according to the manufacturer's instructions. Sequencing was performed on the PromethION (ONT) platform. In order to polish the assembly, genomic DNA was extracted from the foot muscle tissue of the same individual using the E.Z.N.A Mollusc DNA Kit and short-read sequencing of a library with an insert length of ~350bp was performed on the DNBSEQ-T7 system." on page 6 line 20 to page 7 line 7.
- Page 10, line 10: "The Hi-C reads accounts for ~138X coverage of the L. brevicula genome and~123X coverage…". Perhaps, this sentence should be at the beginning of the results where the authors also mention the coverage of the PacBio and ONT. There, they should also mention the coverage of the Illumina made to polish the ONT assembly.
Response: Thank you for your suggestion. We have removed the description of the coverage of Hi-C sequencing in "Materials and Methods" to section "Genome assembly and annotation of two littorinid snails" in "Results". The coverage of the DNBSEQ-T7 short reads used for polishing the ONT assembly was also mentioned in "Results" page 12 line 6 (~176-fold coverage).
- Page 11, line 8: Why using ALLHiC for the scaffolding of the L. sinensis genome and not for that L. brevicula?
Response: Thank you for pointing out this issue. The Hi-C reads were firstly anchored to both littorinids using the software 3D-DNA but it didn't work well with L. sinensis and the resulting assembly was fragmented. Therefore, we used ALLHiC and finally got the satisfying chromosome-level genome assembly for L. sinensis.
- Page 12, line 1: What tissue was the source for the transcriptome?
Response: Thank you for pointing out this issue. The tissue for transcriptome was from the foot muscle tissues of these two littorinids. We have made it clear in the revised manuscript page 8 line 19.
- Page 12, line 10: why not including here any e.g., Lautoconus ventricosus, which also has a chromosome-level genome?
Response: Thank you for pointing out this issue. When we chose the species used in the genome annotation, we directly downloaded data from MolluscDB (http://download.mgbase.qnlm.ac/page/download/downloadall/) which has not included the genomic data of Lautoconus ventricosus. It is regrettable to miss such a well assembled genome.
- Page 12, line 11: "caniculata"
Response: Thank you for pointing out this. We have corrected the name of the apple snail into "Pomacea canaliculata" on page 9 line 8.

- Page 12, line 4 from the end. Why using here a list of species different to that used in METAEUK? Please explain the rationale behind the selection of species in the different analyses.
Response: Thank you for pointing out this issue. As for homologue-based annotation, we expected to identify as many homologous genes as possible, so the genomic data used here in METAEUK were mainly from gastropoda snails which are supposed to contain more homologous genes with littorinids than those species used for comparative genomic analyses in which we focused on the difference of habitats instead of phylogenetic relationship.
- Page 13, line 9. Three time "estimates" retrieved… were used for "calibration". The Timetree database has estimates from different studies that have performed chronogram inferences based on fossil or geographic data. I would suggest using directly fossils to calibrate the tree. You can check the Paleobiology Database (https://paleobiodb.org/) and see, for example, that there are fossils of Littorina from the middle Triassic of Germany (247 MYA) and of Littoraria from the Danian of Denmark (66 MYA). In any case, if you maintain the estimates from Timetree, please write down the dates used as well as models, priors, and other useful information to interpret the inferred chronogram. Check also (and discuss) the dates inferred in Williams et al. (2003) A molecular phylogeny of the Littorininae (Gastropoda: Littorinidae): unequal

evolutionary rates, morphological parallelism, and biogeography of the Southern Ocean. Mol. Phylogenet. Evol. 28: 60-86.
Response: Thank you for pointing out this issue. Here in our work, 3 calibration points were used to estimate the divergence time for nodes in the phylogenetic tree: the divergence time between Capitella teleta and molluscan species (534.3-654.0 Mya) from citation 75; the divergence time between Nautilus pompilius and Bivalvia and Gastropoda species (527.6-619.1 Mya) from citation 75; the divergence time between Chlamys farreri and Patinopecten yessoensis (46.1-71.7 Mya) from the Timetree database (http://www.timet ree.org/) and citation 76 . This part is rewritten in the “Gene family, phylogenetic analysis, and divergence time estimation” section in “Material and Methods” (page10 line 6-10) and three calibration points were marked using red squares in Figure 1. The estimated divergence time of the two littorinids was compared with results from previous studies and discussed in the second paragraph of Discussion.
- Page 14, line 3: It is not clear to me that the comparison of four "submerged" species versus the two periwinkles will be enough to identify genes related to adaptation to the stress associated to low tides. There are too many other life-history differences that may act as confounding factors.
Response: Thank you for pointing out this issue. Considering the divergence time of the littorinid snails and the closest related lineage was over 400Mya, the genes that was identified to be positively selected may be related with many differences like life-history instead of just intertidal adaptation. So in the revised manuscript, FDR correction was performed on the results ($p < 0.01$) and the positively selected genes were functionally annotated, based on which only 85 genes were considered to be potential candidate genes for intertidal adaptation. The discussion was only about this potential candidate gene set on page 16 line 19 – page 17 line 3.
- Page 15. Results are generally well written!
Response: Thank you for your compliment!
- Page 15, line 1: indicate that the PacBio technology is CLR. Add the coverage of the Illumina reads used for polishing the ONT assembly.
Response: Thank you for your suggestion. We have made it clear that the PacBio technology is CLR and added the coverage (~176-fold coverage) of the DNBSEQ-T7 short reads used for polishing the ONT assembly in page 12 line 3-7.
- Page 17, line 1: "might facilitate littorinids to respond to biotic and abiotic stresses and adapt to the intertidal environment" is not a result, should go to discussion.
Response: Thank you for your suggestion. We have deleted this sentence in the “Result” section and rewritten the intertidal environment adaptation part in the “Discussion” section in page 16.
- Page 17, line 7: "as strong candidate genes for intertidal adaptation" to discussion.
Response: Thank you for your suggestion. We have deleted this sentence in the “Results” section and rewritten the intertidal environment adaptation part in the “Discussion” section in page 16.
- Page 18, line 1: "Previous macrosynteny… ALG16 to PY2) to discussion.
Response: Thank you for your suggestion. We have moved this part into Discussion in the revised manuscript.
- Page 18, first line of discussion: rewrite the sentence "Littorinid snails, as candidate ecological and evolutionary models, require urgently to have reference genomes sequenced at high quality".
Response: Thank you for your suggestion. We have rewritten this sentence into “As candidate ecological and evolutionary models, high-quality genomes are urgently needed for littorinid snails.” in the revised manuscript on page 15 line 5-6.
- Page 18, line 3: Any discussion here on the differences between using PacBio vs ONT?
Response: Thank you for pointing out this issue. Sequencing of the two littorinid snails was both performed on the PacBio platform while it failed for L. sinensis. DNA library conduction and sequencing of L. sinensis was then performed by multiple flow cells of the ONT platform from which adequate sequencing data was finally generated. We have added the discussion “Considering the higher accuracy of PacBio platform than the ONT platform, long-read sequencing of the two littorinid snails was performed by using the PacBio platform at the beginning. However, PacBio sequencing for L. sinensis failed possibly due to mucopolysaccharides that might block the zero-model waveguides (ZMWs). Genomic library construction and sequencing of L. sinensis was then performed by multiple flow cells of the ONT platform from which adequate sequencing data was finally generated.” in the revised manuscript on page 15 line 6-11.
- Page 19, line 1: This large paragraph explains the very diverse functions attributed to proteins, whose gene families were found to be expanded in the comparison between submerged and intertidal species. The authors try to show that all these functions might end having a role in counteracting the stress associated

to low tides but the paragraph is rather narrative and not very convincing given the design of the comparison and the lack of any direct evidence or functional assay. I think that this paragraph is rather speculative and needs to be toned down.
Response: Thank you for pointing out this. This paragraph was too speculative and redundant for a data note. We have simplified the discussion about the expanded gene families of littorinid lineage in page 16.
- Page 20, line 14: Again this paragraph is rather speculative and in particular the very last sentence is not sustained by the data, analyses or literature; "the maintenance of cellular homeostasis and repairing of damaged nucleotides and proteins were main strategies for littorinid snails to hinder the cell apoptosis processes caused by environmental stresses and facilitate adaptation to the harsh intertidal environment."
Response: Thank you for pointing out this. It was inappropriate to define the main strategies for intertidal environment adaptation based on the results of this manuscript. We have simplified the discussion about the positively selected genes and rewritten this part at the bottom of page 16 and the beginning of page 17.
- Page 22, line 7 from the end. "By overcoming technical challenges". I guess no more that those overcome when sequencing the high-quality genomes of e.g., Chrysomalion, Pomacea or Lautoconus. Please delete.
Response: Thank you for your suggestion. We have deleted the “Conclusion” section in the revised manuscript according to the suggestion of another reviewer since it largely repeats aspects of the “Discussion”.
- Page 23, line 1. "and finally facilitated the adaptation to harsh intertidal environments with multiple biotic and abiotic stresses". This is too speculative. Please delete.
Response: Thank you for your suggestion. We have deleted the “Conclusion” section in the revised manuscript according to the suggestion of another reviewer since it largely repeats aspects of the “Discussion”.
- References: I find that for a data note, 121 are too many references. Could the authors try to use less?
Response: Thank you for your suggestion. We have made some deletion and only 88 references are reserved in the revised manuscript.
- Figure 1: Please add credibility intervals to the date estimates. Please add geological periods in the bottom line.
Response: Thank you for your suggestion. We have added credibility intervals to the date estimates and geological periods in the revised Figure 1.
- Table 3 &4: These are too technical; perhaps they could be moved to supplementary material
Response: Thank you for your suggestion. We have moved these two tables into supplementary material in the revised manuscript.
