# Supplementary material for: Chromosome-level genome assemblies of two littorinid marine snails indicate genetic basis of intertidal adaptation and ancient karyotype evolved from bilaterian ancestors
Source: Gigascience. 2024 Sep 25;13:giae072. doi: 10.1093/gigascience/giae072 (PMC11423352; doi:10.1093/gigascience/giae072)
Supplement: giae072_Response_to_Reviewer_Comments_Revision_1 [file giae072_response_to_reviewer_comments_revision_1.pdf]

Author's Response To Reviewer Comments

Response to Reviewer 1 Comments
Reviewer #1 general comments: The authors have made many constructive changes to this MS in line with comments on the original version. The contribution represented by these two new genomes is certainly a valuable one and the level of justification and interpretation is now more appropriate for the type of manuscript. However, two issues raised by me and by the other reviewer still need some attention.
First, positively selected genes in littorinids are, indeed, potential candidates for intertidal adaptation, but they are also potential candidates for many other adaptive responses - singling out the intertidal environment remains a problem. See specific comments below. I think that only a few small changes are needed to acknowledge this limitation.
Second, Figure 3 is improved but I think the authors need to justify their conclusion that the scallop-littorinid ancestral karyotype was the same as the P. yessoensis karyotype and acknowledge that this is only one possible interpretation.
The abstract needs to be adjusted to ensure that it also reflects appropriate caution on both of these points. In line with Rev. 2's previous comment, it would be better to separate the two issues in the first sentence into separate sentences.

Response: We would like to thank you for your careful reading, helpful comments, and constructive suggestions, which have significantly improved the presentation of our manuscript.
We have carefully considered all comments from the reviewers and revised our manuscript accordingly. The manuscript has also been double-checked, and the typos and grammar errors we found have been corrected. We have discussed the limitation of the comparative genomic analyses and acknowledged that the molluscan ancestor karyotype layout in Fig. 3 was just the most parsimonious possible state and revised Fig. 3. The abstract has been adjusted and the two main topics in the manuscript were separated in the revised abstract. In the following section, we summarize our responses to each comment. The rewritten text in the revised manuscript were highlighted with yellow marks. We believe that our responses have well addressed all the concerns of the reviewers. We hope our revised manuscript can be accepted for publication.

Specific comments by Page:
Reviewer comment: line 3:9-10 - replace 'genetic mechanistic basis' with something like 'mechanistic basis at the genetic level'
Response: Thank you for your suggestion. We have rewritten this sentence into "To better understand how they survive in and adapt to fluctuating environments, it is crucial to elucidate the mechanistic basis at genetic level." on page 3 line 8-10.
Reviewer comment:4:22 - 'De Jode et al.' rather than 'Jode et al.'
Response: Thank you for your correction. We are very sorry for making a mistake on the author's name and we have made the correction on page 4 line 22.
Reviewer comment:6:2-3 - needs to be re-worded, at least, to be clear that adaptation to the intertidal is just one of many possible drivers of gene family and sequence evolution.
Response: Thank you for pointing out this issue. The original paragraph may mislead readers into considering all genetic signals discovered by comparative genomic analyses as genetic mechanisms associated with adaptation to intertidal. We have rewritten this sentence into "Comparative genomic analyses were performed to investigate genetic mechanisms potentially associated with adaptation to intertidal harsh and fluctuating environments, which is one of many possible drivers of gene family and sequence evolution in the littorinid lineage." on page 6 line 2 to 5.
Reviewer comment:11:1-2 - this section should just be called 'Identification of positively selected genes' because all genes with signatures of positive selection are extracted, with no way to limit this to genes related to the colonisation of the intertidal.
Response: Thank you for your suggestion. We have changed the title of this section into "Identification of positively selected genes".
Reviewer comment:Fig. 1 - note that 'laevigata' is missing the final 'a'
Response: Thank you for pointing out this issue. We have now made a correction for Haliotis laevigata in both the MS and Fig. 1.

Reviewer comment:13:11 - a revised title would also be appropriate here because the section describes expanded gene families in the basal littorinid branch, and evidence for positive selection in that branch, with no way to distinguish the specific effects of the intertidal environment.
Response: Thank you for your suggestion. We have changed the title of this section into "Expanded gene families and positively selected genes".
Reviewer comment:16:10 - 17:3 - it is essential that this paragraph includes some recognition of the fact that changes on the relevant branch of the phylogenetic tree may have been driven by factors other than adaptation to the intertidal environment. The branch covers >200My, with no information on the timing of intertidal colonisation within that time-span and also no information on the timing of positive selection or gene-family expansion relative to colonisation.
Response: Thank you for pointing out this issue. We have added the discussion about hypotheticals of our result in the revised manuscript on page 17 line 3-9.
Reviewer comment:17:8-15 - the authors have not fully accounted for my previous comments here. The text and Fig. 3 still imply a particular sequence of events, which equates the P. yessoensis karyotype with the karyotype of the common ancestor of scallops and littorinids. Overall, this implies 4 fissions and 4 fusions. Is this the most parsimonious reconstruction? Does it remain the most parsimonious history if fusions are considered more likely than fissions? Fig. 3 still tends to imply, incorrectly, that the littorinid karyotype was derived from the scallop karyotype (and that no change occurred on the branch from the common ancestor to P. yessoensis).
Response: Thank you for pointing out this issue. It is barely possible to infer the real karyotype of the common ancestor of scallops and littorinids just using three genomes. After considering different possible chromosome evolution trajectories, we suggested that the karyotype that was the same as that of P. yessoensis was the most parsimonious karyotype of the common ancestor of scallops and littorinids. So in the revised version, we added this part into the manuscript on page 18 line 3-11 and we put the chromosomes of the scallop and littorinids into the same layout to make it clear that the littorinid karyotype is NOT evolved directly from that of the scallop, but from the common ancestor of scallops and littorinids (Figure 3).
Reviewer comment:17:19-22 - this is true for the reconstruction shown in Fig. 3, which may be the most parsimonious, but is not the known history.
Response: Thank you for pointing out this issue. Although the 3 chromosomal fissions and 1 chromosomal fusion between the bilaterian ancestors and P. yessoensis were also found between littorinid snails and the bilaterian ancestors, it is still just a possible speculation based on the most parsimonious reconstruction of the karyotype for the common ancestor. We have written this sentence in the revised manuscript on page 18 line 3-11.
Reviewer comment:18:1-4 - yes, but again based on one possible reconstruction
Response: Thank you for pointing out this issue. We have rewritten this sentence in the revised manuscript on page 18 line 3-11.
