# Supplementary material for: Chromosome-level genome assemblies of two littorinid marine snails indicate genetic basis of intertidal adaptation and ancient karyotype evolved from bilaterian ancestors
Source: Gigascience. 2024 Sep 25;13:giae072. doi: 10.1093/gigascience/giae072 (PMC11423352; doi:10.1093/gigascience/giae072)
Supplement: giae072_Reviewer_1_Report_Original_Submission — Roger Butlin -- 4/18/2024 Reviewed [file giae072_reviewer_1_report_original_submission.pdf]

Reviewer Report

Title: Chromosome-level genome assemblies of two littorinid marine snails indicate genetic basis of intertidal adaptation and ancient karyotype evolved from bilaterian ancestors

Version: Original Submission **Date:** 4/18/2024

Reviewer name: Roger Butlin

Reviewer Comments to Author:

This MS reports genome assemblies at chromosomal scale for two littorinid gastropods. This is an exciting contribution because littorinids are widely-used models in evolutionary ecology but it has been challenging to achieve high-quality genomes due to technical difficulties with DNA extractions. Another littorinid genome has recently been published, at similar level of completeness, but the contribution here remains valuable for comparative purposes and because the annotation in this paper seems better than in the L. saxatilis case.I am not an expert on the assembly and annotation methods and so have few comments on these aspects.Two larger analyses are developed using the new genomes. The first is an attempt to identify genes and gene families implicated in adaptation to the stressful intertidal environment exploited by littorinids. Only one other gastropod is used in this comparison and the branch to littorinids is simply too long to detect a signal that can be attributed to intertidal adaptation. The lists of expanded gene families and genes with evidence of positive selection are interesting for future study but it is premature to speculate about their possible roles in a particular environment.The second issue concerns deep conservation of linkage groups. Largely conserved macrosynteny is observed on the very long scallop-littorinid branch. This is interesting, Extending the deeper patterns found by Simakov and others (including the extensive within-chromosome shuffling).The Discussion contains a lot about expanded gene families and putatively selected genes on the long branch. This should be reduced. On macrosynteny, the Discussion note the main addition, which is to push most of the changes previously reported in the scallop lineage back before the bivalve-gastropod split. Revision of Fig. 3 (see below) would help to make this point.Specific comments follow (Line numbers would have been helpful):P4, bottom - a helpful citation here would be Johannesson et al. 2024 Trends in GeneticsP5, top - citation 17 does not mention bacteria or viruses and so seems inappropriate here.P5, mid, and P6 - note that a high-quality genome with annotation is now available for Littorina saxatilis: GBE https://doi.org/10.1093/gbe/evae076P6, bottom - it is unsafe to claim that gastropods are unchanged over these long time-periods because we know little beyond the form of the shell for early taxa.P8, mid - the papers by Simakov and coworkers show that multiple taxa have karyotypes that can be related to the bilaterian ALGs (indeed, this is how the ALGs were identified). Therefore, the gross similarity of the littorinid LGs with the ALGs does not make littorinids particularly strong models for the study of early bilaterian evolution.P10, bottom - I would have expected a duplicate removal step in this pipeline although I note that duplicated BUSCOs are not commonP14, top - generic names should be given here in full. Haliotis is the only gastropod in this background set and it is in a different subclass from Littorinidae (common ancestor more than 400Mya). Therefore, there is the opportunity for many differences to have evolved under positive selection that are unrelated to the intertidal environment.

The test for positive selection should be adjusted for multiple comparisons since many genes were investigated without a priori predictions.Table 1 - the meaning of 'Main genome' should be explained in the legendFig. 3 - this would be much more legible if LGs were re-ordered, rather than following their (arbitrary) numbering.P16 - either a mutation rate must have been used to infer times, or a calibration point or points, but I did not find the rate or source of calibration in the MS. The split time given here (128My) is much older than in Reid et al 2012 (90-90My).P17, bottom - 'collinearity' is not a good word here, because of the extensive within-chromosome rearrangementsFigure 3 - a better layout could be found. The current layout risks implying that evolution proceeded from the ALG arrangement, to the PYE arrangement and then to the littorinid arrangement. In reality, there was a step from the ALG to a common molluscan ancestral state and then, independently, to the PYE and littorinid tips. Some features of the ancestral molluscan state can be inferred and so a layout that showed this node would be helpful.P21, bottom - I don't think that synteny between L. brevicula and L. sinensis is shown at any point (despite mention here).P22, top - care is needed here - it is only the gross karyotype that is shown to be 'slow-evolving' and one should not imply slow evolution of any other aspect of gastropod or littorinid biology.P22-23 - the Conclusions section largely repeats aspects of the Discussion and is probably not needed.

Level of Interest

Please indicate how interesting you found the manuscript: Choose an item.

Quality of Written English

Please indicate the quality of language in the manuscript: Choose an item.

Declaration of Competing Interests

Please complete a declaration of competing interests, considering the following questions:

- Have you in the past five years received reimbursements, fees, funding, or salary from an organisation that may in any way gain or lose financially from the publication of this manuscript, either now or in the future?
- Do you hold any stocks or shares in an organisation that may in any way gain or lose financially from the publication of this manuscript, either now or in the future?
- Do you hold or are you currently applying for any patents relating to the content of the manuscript?
- Have you received reimbursements, fees, funding, or salary from an organization that holds or has applied for patents relating to the content of the manuscript?
- Do you have any other financial competing interests?
- Do you have any non-financial competing interests in relation to this paper?

If you can answer no to all of the above, write 'I declare that I have no competing interests' below. If your reply is yes to any, please give details below.

I declare that I have no competing interests

I agree to the open peer review policy of the journal. I understand that my name will be included on my report to the authors and, if the manuscript is accepted for publication, my named report including any attachments I upload will be posted on the website along with the authors' responses. I agree for my report to be made available under an Open Access Creative Commons CC-BY license (http://creativecommons.org/licenses/by/4.0/). I understand that any comments which I do not wish to be included in my named report can be included as confidential comments to the editors, which will not be published.

Choose an item.

To further support our reviewers, we have joined with Publons, where you can gain additional credit to further highlight your hard work (see: https://publons.com/journal/530/gigascience). On publication of this paper, your review will be automatically added to Publons, you can then choose whether or not to claim your Publons credit. I understand this statement.

Yes Choose an item.
