# Supplementary material for: Chromosome-level genome assemblies of two littorinid marine snails indicate genetic basis of intertidal adaptation and ancient karyotype evolved from bilaterian ancestors
Source: Gigascience. 2024 Sep 25;13:giae072. doi: 10.1093/gigascience/giae072 (PMC11423352; doi:10.1093/gigascience/giae072)
Supplement: giae072_Reviewer_1_Report_Revision_1 — Roger Butlin -- 6/27/2024 Reviewed [file giae072_reviewer_1_report_revision_1.pdf]

Reviewer Report

Title: Chromosome-level genome assemblies of two littorinid marine snails indicate genetic basis of intertidal adaptation and ancient karyotype evolved from bilaterian ancestors

Version: Revision 1 **Date:** 6/27/2024

Reviewer name: Roger Butlin

Reviewer Comments to Author:

The authors have made many constructive changes to this MS in line with comments on the original version. The contribution represented by these two new genomes is certainly a valuable one and the level of justification and interpretation is now more appropriate for the type of manuscript. However, two issues raised by me and by the other reviewer still need some attention.First, positively selected genes in littorinids are, indeed, potential candidates for intertidal adaptation, but they are also potential candidates for many other adaptive responses - singling out the intertidal environment remains a problem. See specific comments below. I think that only a few small changes are needed to acknowledge this limitation.Second, Figure 3 is improved but I think the authors need to justify their conclusion that the scallop-littorinid ancestral karyotype was the same as the P. yessoensis karyotype and acknowledge that this is only one possible interpretation.The abstract needs to be adjusted to ensure that it also reflects appropriate caution on both of these points. In line with Rev. 2's previous comment, it would be better to separate the two issues in the first sentence into separate sentences.Specific comments by Page:line3:9-10 - replace 'genetic mechanistic basis' with something like 'mechanistic basis at the genetic level'4:22 - 'De Jode et al.' rather than 'Jode et al.'6:2-3 - needs to be re-worded, at least, to be clear that adaptation to the intertidal is just one of many possible drivers of gene family and sequence evolution.11:1-2 - this section should just be called 'Identification of positively selected genes' because all genes with signatures of positive selection are extracted, with no way to limit this to genes related to the colonisation of the intertidal.Fig. 1 - note that 'laevigata' is missing the final 'a'13:11 - a revised title would also be appropriate here because the section describes expanded gene families in the basal littorinid branch, and evidence for positive selection in that branch, with no way to distinguish the specific effects of the intertidal environment.16:10 - 17:3 - it is essential that this paragraph includes some recognition of the fact that changes on the relevant branch of the phylogenetic tree may have been driven by factors other than adaptation to the intertidal environment. The branch covers >200My, with no information on the timing of intertidal colonisation within that time-span and also no information on the timing of positive selection or gene-family expansion relative to colonisation.17:8-15 - the authors have not fully accounted for my previous comments here. The text and Fig. 3 still imply a particular sequence of events, which equates the P. yessoensis karyotype with the karyotype of the common ancestor of scallops and littorinids. Overall, this implies 4 fissions and 4 fusions. Is this the most parsimonious reconstruction? Does it remain the most parsimonious history if fusions are considered more likely than fissions? Fig. 3 still tends to imply, incorrectly, that the littorinid karyotype was derived from the scallop karyotype (and that no change occurred on the branch from the common ancestor to P. yessoensis).17:19-22 - this is true for the reconstruction shown in Fig. 3, which

may be the most parsimonious, but is not the known history.18:1-4 - yes, but again based on one possible reconstruction

Level of Interest

Please indicate how interesting you found the manuscript: Choose an item.

Quality of Written English

Please indicate the quality of language in the manuscript: Choose an item.

Declaration of Competing Interests

Please complete a declaration of competing interests, considering the following questions:

- Have you in the past five years received reimbursements, fees, funding, or salary from an organisation that may in any way gain or lose financially from the publication of this manuscript, either now or in the future?
- Do you hold any stocks or shares in an organisation that may in any way gain or lose financially from the publication of this manuscript, either now or in the future?
- Do you hold or are you currently applying for any patents relating to the content of the manuscript?
- Have you received reimbursements, fees, funding, or salary from an organization that holds or has applied for patents relating to the content of the manuscript?
- Do you have any other financial competing interests?
- Do you have any non-financial competing interests in relation to this paper?

If you can answer no to all of the above, write 'I declare that I have no competing interests' below. If your reply is yes to any, please give details below.

None

I agree to the open peer review policy of the journal. I understand that my name will be included on my report to the authors and, if the manuscript is accepted for publication, my named report including any attachments I upload will be posted on the website along with the authors' responses. I agree for my report to be made available under an Open Access Creative Commons CC-BY license (http://creativecommons.org/licenses/by/4.0/). I understand that any comments which I do not wish to be included in my named report can be included as confidential comments to the editors, which will not be published.

Choose an item.

To further support our reviewers, we have joined with Publons, where you can gain additional credit to further highlight your hard work (see: https://publons.com/journal/530/gigascience). On publication of this paper, your review will be automatically added to Publons, you can then choose whether or not to claim your Publons credit. I understand this statement.

Yes Choose an item.
