# Supplementary material for: Chromosome-level genome assemblies of two littorinid marine snails indicate genetic basis of intertidal adaptation and ancient karyotype evolved from bilaterian ancestors
Source: Gigascience. 2024 Sep 25;13:giae072. doi: 10.1093/gigascience/giae072 (PMC11423352; doi:10.1093/gigascience/giae072)
Supplement: giae072_Reviewer_2_Report_Original_Submission — Rafael Zardoya, PhD -- 4/21/2024 Reviewed [file giae072_reviewer_2_report_original_submission.pdf]

Reviewer Report

Title: Chromosome-level genome assemblies of two littorinid marine snails indicate genetic basis of intertidal adaptation and ancient karyotype evolved from bilaterian ancestors

Version: Original Submission **Date:** 4/21/2024

Reviewer name: Rafael Zardoya, PhD

Reviewer Comments to Author:

The paper entitled "Chromosome-level genome assemblies of two littorinid marine snails reveal the genetic basis of intertidal adaptation and ancient karyotype evolved from bilaterian ancestors" by Wang et al presents the chromosome-level genomes of two periwinkles. One was assembled based on PacBio reads and the other based on ONT sequence data. Both were scaffolded into pseudo-chromosomes based on HiC data. In both cases, final genome statistics (length, N50, BUSCO) are correct, indicating the high quality in terms of contiguity and completeness of the new genomes. However, I miss some information on Phred quality scores (accuracy), particularly in the case of the ONT sequencing, which rendered only 20x coverage. In any case, the new genomes will be very useful and thus the interest of this data note.I have several suggestions that the authors may want to consider when revising the manuscript:- title: I think "reveal" is too strong as the authors have only detected candidate genes and no functional assay has been performed- abstract, line 1: delete "evolutionary" before "adaptation"- abstract, line 1: understanding adaptation to environments and the early evolution of bilaterians are two important aims of evolutionary biology but very different and I am not sure they can be combined in the same sentence.- abstract, line 8: I find no support in the study to assert that the mentioned gene families "played key roles in adaptation to multiple biotic and abiotic stresses in intertidal environments".- Page 3, line 7: "processes" instead of "process"- Page 3, line 7: "evolve" instead of "acquire"- Page 3, line12: please sustain better with references that the genetic basis of adaptation to fluctuating environments remains mostly unknown.- Page 3, line 2 from the end: rhythm? Instead of "rhyme"- Page 4: This is a data note and in this regard, I find the introduction from this page on rather long and verbose. In page 4, I suggest deleting many sentences and focusing directly on the Littorinidae (line 13).- Page 5, line 2: rhythm? Instead of "rhyme"- Page 5, line 6: are really periwinkles the best model to understand adaptation to climate change? Please support this asseveration.- Page 5, line 15 to Page 6, line 7: these sentences can be deleted to maintain the focus of the introduction. Start the paragraph with "Yet, no …"- Page 6, line 4: from the end: I would say that is a key question for evolutionary bioloy not only for the Evo-Devo field.- Page 6, last line: "almost unchanged from their ancestors". This asseveration is simply not true. After hundred million years of evolution, extant gastropods have diverged considerably from their ancestors. The authors may be referring to the external resemblance of shells in extant and fossil snails. Please delete this sentence.- Page 7, line 2: The gastropods are as good as e.g., bivalves, cephalopods, caudofoveates to represent mollusks in comparative studies on the bilaterian ancestor, and mollusks are as good as any other phylum for this purpose. Please delete or rewrite this sentence.- Page 7, lines 5-8: These sentences can be deleted to alleviate the introduction.- Page 8, line 1: add "including detection of expanding/ contracting gene

families and positively selected genes" before "were performed", and the delete sentences from line 2 to 12 (end of introduction).- Page 8, line 3 from the end: Please go straight to what worked and delete this whole paragraph (that continues in page 9).- Page 9, line 12: delete "genomic" before "DNA" and add after "from a single individual". Please specify the source tissue (foot?)- Page 9, line 13: delete "for genome assembly and correction". Delete "of a Qingdao L. brevicula individual"- Page 9, line 15: Please specify that you used the CLR and not the HiFi technology of PacBio.- Page 9, last line and page 10 first paragraph: I suggest first explaining the ONT procedure and then explaining that in order to polish the assemblies, an Illumina sequencing was performed on the DNBSEQ-T7 system. Please specify the source tissue (foot?)- Page 10, line 10: "The Hi-C reads accounts for ~138X coverage of the L. brevicula genome and~123X coverage…". Perhaps, this sentence should be at the beginning of the results where the authors also mention the coverage of the PacBio and ONT. There, they should also mention the coverage of the Illumina made to polish the ONT assembly.- Page 11, line 8: Why using ALLHiC for the scaffolding of the L. sinensis genome and not for that L. brevicula?- Page 12, line 1: What tissue was the source for the transcriptome?- Page 12, line 10: why not including here any e.g., Lautoconus ventricosus, which also has a chromosome-level genome?- Page 12, line 11: "caniculata"- Page 12, line 4 from the end. Why using here a list of species different to that used in METAEUK? Please explain the rationale behind the selection of species in the different analyses.- Page 13, line 9. Three time "estimates" retrieved… were used for "calibration". The Timetree database has estimates from different studies that have performed chronogram inferences based on fossil or geographic data. I would suggest using directly fossils to calibrate the tree. You can check the Paleobiology Database (https://paleobiodb.org/) and see, for example, that there are fossils of Littorina from the middle Triassic of Germany (247 MYA) and of Littoraria from the Danian of Denmark (66 MYA). In any case, if you maintain the estimates from Timetree, please write down the dates used as well as models, priors, and other useful information to interpret the inferred chronogram. Check also (and discuss) the dates inferred in Williams et al. (2003) A molecular phylogeny of the Littorininae (Gastropoda: Littorinidae): unequal evolutionary rates, morphological parallelism, and biogeography of the Southern Ocean. Mol. Phylogenet. Evol. 28: 60-86.- Page 14, line 3: It is not clear to me that the comparison of four "submerged" species versus the two periwinkles will be enough to identify genes related to adaptation to the stress associated to low tides. There are too many other life-history differences that may act as confounding factors.- Page 15. Results are generally well written!- Page 15, line 1: indicate that the PacBio technology is CLR. Add the coverage of the Illumina reads used for polishing the ONT assembly.- Page 17, line 1: "might facilitate littorinids to respond to biotic and abiotic stresses and adapt to the intertidal environment" is not a result, should go to discussion.- Page 17, line 7: "as strong candidate genes for intertidal adaptation" to discussion.- Page 18, line 1: "Previous macrosynteny… ALG16 to PY2) to discussion.- Page 18, first line of discussion: rewrite the sentence "Littorinid snails, as candidate ecological and evolutionary models, require urgently to have reference genomes sequenced at high quality".- Page 18, line 3: Any discussion here on the differences between using PacBio vs ONT?- Page 19, line 1: This large paragraph explains the very diverse functions attributed to proteins, whose gene families were found to be expanded in the comparison between submerged and intertidal species. The authors try to show that all these functions might end having a role in counteracting the stress associated to low tides but the paragraph is rather narrative and not very convincing given the design of the comparison and the lack of any direct evidence or functional assay. I think that this paragraph is

rather speculative and needs to be toned down.- Page 20, line 14: Again this paragraph is rather speculative and in particular the very last sentence is not sustained by the data, analyses or literature; "the maintenance of cellular homeostasis and repairing of damaged nucleotides and proteins were main strategies for littorinid snails to hinder the cell apoptosis processes caused by environmental stresses and facilitate adaptation to the harsh intertidal environment."- Page 22, line 7 from the end. "By overcoming technical challenges". I guess no more that those overcome when sequencing the high-quality genomes of e.g., Chrysomalion, Pomacea or Lautoconus. Please delete.- Page 23, line 1. "and finally facilitated the adaptation to harsh intertidal environments with multiple biotic and abiotic stresses". This is too speculative. Please delete.- References: I find that for a data note, 121 are too many references. Could the authors try to use less?- Figure 1: Please add credibility intervals to the date estimates. Please add geological periods in the bottom line.- Table 3 &4: These are too technical; perhaps they could be moved to supplementary material

Level of Interest

Please indicate how interesting you found the manuscript: Choose an item.

Quality of Written English

Please indicate the quality of language in the manuscript: Choose an item.

Declaration of Competing Interests

Please complete a declaration of competing interests, considering the following questions:

- Have you in the past five years received reimbursements, fees, funding, or salary from an organisation that may in any way gain or lose financially from the publication of this manuscript, either now or in the future?
- Do you hold any stocks or shares in an organisation that may in any way gain or lose financially from the publication of this manuscript, either now or in the future?
- Do you hold or are you currently applying for any patents relating to the content of the manuscript?
- Have you received reimbursements, fees, funding, or salary from an organization that holds or has applied for patents relating to the content of the manuscript?
- Do you have any other financial competing interests?
- Do you have any non-financial competing interests in relation to this paper?

If you can answer no to all of the above, write 'I declare that I have no competing interests' below. If your reply is yes to any, please give details below.

I declare that I have no competing interests

I agree to the open peer review policy of the journal. I understand that my name will be included on my report to the authors and, if the manuscript is accepted for publication, my named report including any attachments I upload will be posted on the website along with the authors' responses. I agree for my report to be made available under an Open Access Creative Commons CC-BY license (http://creativecommons.org/licenses/by/4.0/). I understand that any comments which I do not wish to be included in my named report can be included as confidential comments to the editors, which will not be published.

Choose an item.

To further support our reviewers, we have joined with Publons, where you can gain additional credit to further highlight your hard work (see: https://publons.com/journal/530/gigascience). On publication of this paper, your review will be automatically added to Publons, you can then choose whether or not to claim your Publons credit. I understand this statement.

Yes Choose an item.
